# Ten New Dammarane-Type Saponins with Hypolipidemia Activity from a Functional Herbal Tea—*Gynostemma pentaphyllum*

**DOI:** 10.3390/molecules25163737

**Published:** 2020-08-15

**Authors:** Maojing Yin, Jingjing Zhang, Lizhi Wang, Fangyi Li, Zhenfa Li, Wei Xiang, Songtao Bie, Chunhua Wang, Zheng Li

**Affiliations:** 1 College of Pharmaceutical Engineering of Traditional Chinese Medicine, Tianjin University of Traditional Chinese Medicine, Tianjin 301617, China; yinmaojing11@163.com (M.Y.); zjj20190924@163.com (J.Z.); li_fangyi@hotmail.com (F.L.); li-zhenfa@hotmail.com (Z.L.); 15922162979@163.com (W.X.); biesongtao@126.com (S.B.); 2 Tianjin Key Laboratory of Modern Chinese Medicine, Tianjin University of Traditional Chinese Medicine, Tianjin 301617, China; 3 School of Chinese Materia Medicine, Tianjin University of Traditional Chinese Medicine, Tianjin 301617, China; lzhwang_2009@126.com

**Keywords:** *Gynostemma pentaphyllum*, dammarane-type saponins, chemical constituents, isolation and identification, lipid lowering effect

## Abstract

*Gynostemma pentaphyllum* (thumb.) Makino is a functional herbal tea commonly used in Asian countries and regions to reduce blood lipid levels. *G. pentaphyllum* saponin is the main component, but there are still a large number of components with lipid-lowering activity that have not been found. In this study, 10 novel dammarane-type saponins, (**1**–**10**) and a known one (**11**) were isolated from *G. pentaphyllum*. Ten new compounds were identified and named as yunnangypenosides A-J (**1**–**10**), and another known one (**11**) was also obtained. Their chemical structures were determined by MS, NMR spectroscopic analyses. Moreover, the cytotoxicities on human HepG-2 hepatocellular carcinoma cells of these isolates were evaluated, and the results showed that compounds **1**–**11** had no obvious cytotoxicity. Finally, all these compounds were evaluated for their lipid-lowering effect by means of the oil red O staining method. Ten compounds could significantly reduce lipid levels except of **2**, especially **8** exhibite the strongest hypolipidemia activity.

## 1. Introduction

Nowadays, much attention has been paid to people’s physical health, and healthy eating and physical exercise are ways of maintaining general good health. Meanwhile, high-sugar and high-fat diet habits affect people’s health and have been associated with obesity, hypertension, diabetes, dyslipidemia, and other cardiovascular risk factors. Amongst these factors, dyslipidemia has a great impact on human health. It is the main pathogenicity of atherosclerotic cardiovascular disease and an important risk factor for ischemic stroke. So, how to prevent hyperlipidemia through an effective lifestyle has become a topic of increasing concern [1,2,3].

Tea, with leaves or buds from the plant, constitutes one of the beverages popularly consumed in different parts of the world, such as green tea, oolong tea, or black tea. Recent reports demonstrated that some functional tea drinks may exert a positive effect on lowering blood lipid and cholesterol. For example, green tea such as Longjing tea in China has antioxidant and blood lipid lowering effects [4]. The constituents of these tea, including polyphenols, flavonoids, and volatile oil, etc., have several good bioactivities, such as the ability to lowering blood lipid levels and blood pressure, in addition to anti-oxidant protectives and inhibition of inflammation [5,6].

**Figure 1:**
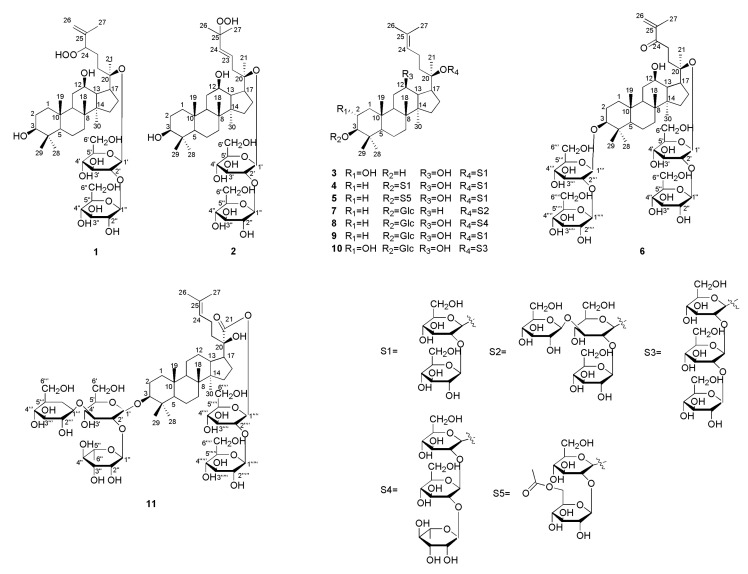
Chemical structures of compounds **1**–**11**.

*Gynostemma pentaphyllum* is a folk medicine and functional herbal tea, which has a good reputation for lowering blood lipid and blood pressure [7,8,9,10,11]. It consists of numerous chemical components, such as saponins, vitamins, polysaccharides, flavonoids, and amino acids [12,13]. Previous studies have shown that *Gypenoside* is a marker component in this plant, and its main chemical components have excellent anti-hyperlipidemia, anti-oxidative, anti-inflammatory, anti-tumor, and other biological activities [11,14,15,16,17]. In view of its good pharmacological activity and edible value, the chemical composition aroused our research group’s interest. Consequently, chemical constituents of *G. pentaphyllum*, especially triterpene saponins, were systematically studied in this paper. The saponins of this plant were isolated and identified by column chromatography (CC) and preparative HPLC methods. As a result, based on the physicochemical properties and spectral data, 11 compounds (Figure 1) were obtained and their structures were determined. The 10 novel compounds were identified and named as yunnangypenosides A–J (**1**–**10**), followed by a known one, 3*β*, 20*S*-dihydroxydammar-24-ene-21-carboxylic acid 3-*O*-{[*α*-l-rhamnopyranosyl-(1→2)]-[*β-*d-glucopyranosyl-(1→3)]-*β*-d-glucopyranosyl}-21-*O*-[*β*-d-glucopyranosyl-(1→2)-*β*-d-glucopyranoside (**11**). Moreover, the toxicities of compounds **1**–**11** were detected using CCK-8 assay (Cell Counting Kit-8), and 11 compounds’ activities in lowering lipid by oil red O staining method in HepG-2 cells were also estimated. The results showed that compounds **1**–**11** had no obvious cytotoxicity, ten compounds (**1**, **3**–**11**) were significant lipid lowering activity, with the exception of **2**, and compound **8** showed the best hypolipidemia activity.

## 2. Results and Discussion

### 2.1. Structural Elucidation

The 60% ethanol extract from the *G. pentaphyllum* was isolated by CC and eluted with different proportions of mobile phase systems to obtain compounds **1**–**10**. Compound **11** was a known one, whose structure could be illustrated by referring the data to those in the literature [12].

**Figure 2:**
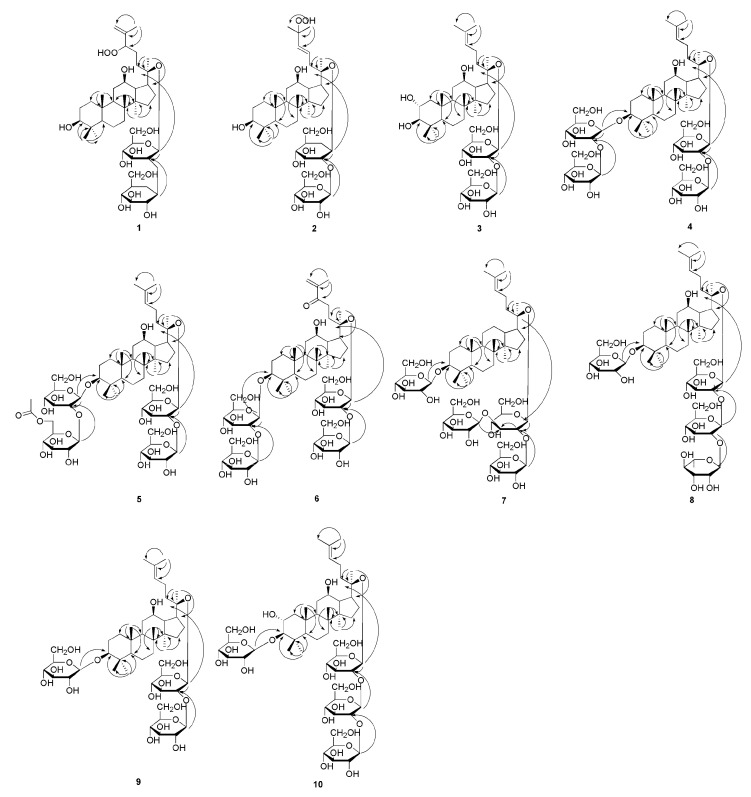
Key HMBC correlations of compounds **1**–**10**.

Compound **1** was light yellow powder and its molecular formula (MF) was C_42_H_72_O_15_, which was inferred by the adduct ion at *m*/*z* 861.4809 [M - H + HCOOH]^−^ (calcd for: 861.4848). Through the NMR spectrum, we could see 42 carbon signals, of which 12 carbons suggested two d-glucopyranosyl units. We analyzed compound **1** by acid hydrolysis and HPLC analysis to determine its sugar units. Subsequently, the *β* configuration for these glucopyranosyl units was identified by the coupling patterns of the anomeric proton signals [δ 5.12 and 5.80] with the same coupling constant (7.8 Hz). The HMBC correlations from δ_H_ 5.12 (Glc’-1) to δ_C_ 84.5 (C-20) and from δ_H_ 5.80 (Glc’’-1) to δ_C_ 80.4 (Glc’-2) suggested that the two *β*-d-glucopyranoyloxy units were linked to C-20 and C-2’, respectively. Besides, we could see seven methyl proton signals at δ 0.87, 0.93, 0.97, 1.03, 1.25, 1.62 and 1.96, along with two olefinic proton signals at δ 5.07 (1H, m) and δ 5.23 (1H, s), were displayed in the ^1^H NMR data (Table 1). The ^13^C NMR (Table 2) and DEPT-135 spectra revealed two anomeric carbon signals at δ 97.0 (Glc’-1) and δ 105.1 (Glc’’-1), two olefinic carbons at δ 113.6 (C-26) and δ 146.4 (C-25), three oxygenated methine signals at δ 71.0 (C-12), δ 78.3 (C-3), and δ 90.3 (C-24), and an oxygenated quaternary carbon at δ 84.5 (C-20). Comparison of the NMR spectrum of **1** with the data of floralquinquenoside D [18] indicated that both of them had the same aglycone moiety. The HMBC data provided the key correlations (Figure 2) between the following atoms: δ 0.97 (H_3_-18) and δ 50.6 (C-9), δ 40.3 (C-8), δ 51.9 (C-14); δ 0.87 (H_3_-19) and δ 50.6 (C-9), δ 39.9 (C-1), δ 37.7 (C-10); δ 1.62 (H_3_-21) and δ 84.5 (C-20), δ 52.9 (C-17), δ 33.2 (C-22); δ 1.96 (H_3_-27) and δ 90.3 (C-24), δ 146.4 (C-25), δ 113.6 (C-26); δ 1.03 (H_3_-29) and δ 78.3 (C-3), δ 56.5 (C-5), δ 29.0 (C-28), δ 39.7 (C-4); δ 0.93 (H_3_-30) and δ 40.3 (C-8), δ 51.9 (C-14), δ 30.8 (C-15). These correlations were characteristic for dammarane-type triterpenoids. The HR-ESI-MS, ^1^H-NMR, ^13^C-NMR, DEPT-135, HMBC, HSQC, and ^1^H-^1^H COSY spectra for compounds **1** can be seen in Figures S1–S7. Thus, the structure of **1** could be elucidated as 3*β*, 12*β*, 20*S*-trihydroxy-24-hydroperoxydammar-25-ene-20-*O*-[*β*-d-glucopyranosyl (1→2)]-*β*-d-glucopyranoside, and named as yunnangypenoside A.

Compound **2** was white powder, with its MF of C_42_H_72_O_15_, which was obtained by adduct ion at *m*/*z* 861.4811 [M - H + HCOOH]^−^ (calcd for, 861.4848). The spectroscopic properties for compound **2** and **1** were similar, with the difference being the peaks on the side chain. The chemical shifts of δ 126.9 (C-23), δ 138.4 (C-24), and δ 81.9 (C-25) revealed that positionC-23, 24had a double bond, while there was an oxygenated quaternary carbon at C-25. The data showed that **2** had 42 carbon atoms, suggesting that there were two d-glucopyranosyl moieties. We analyzed the structure of compound **2** using acid hydrolysis and HPLC analyses, with the same method as used for compound **1** to ascertain the presence of sugar moieties. The *β* configuration for these glucopyranosyl units was proposed using the same method as compound **1**. The HMBC correlations from δ_H_ 5.18 (Glc’-1) to δ_C_ 84.1 (C-20) and from δ_H_ 5.67 (Glc’’-1) to δ_C_ 81.5 (Glc’-2) determined the two *β*-d-glucopyranosyl units located at C-20 and C-2’, respectively. Eight methyl proton signals with chemical shifts at δ 0.93, 0.95, 0.96, 1.05, 1.26, 1.60, 1.62, and 1.62, and two olefinic proton signals at δ 6.02 (1H, d, *J* = 15.8 Hz) and δ 6.23 (1H, m) could be seen in the ^1^H-NMR data of **2** (Table 1). The ^13^C-NMR (Table 2) and DEPT-135 spectra revealed two anomeric carbon signals at δ 97.1 (Glc’-1) and δ 105.9 (Glc’’-1), a pair of olefinic carbons at δ 126.9 (C-23) and δ 138.4 (C-24), two oxygenated methine signals at δ 78.3 (C-3) and δ 71.2 (C-12), and two oxygenated quaternary carbons at δ 84.1 (C-20) and δ 81.9 (C-25). A dammarane skeleton of **2** was identified, for which NMR data were similar to floralginsenoside F [19]. Furthermore, the HMBC data provided the key correlations in Figure 2, which confirmed the above speculative results. The HR-ESI-MS, ^1^H-NMR, ^13^C-NMR, DEPT-135, HMBC, HSQC, and ^1^H-^1^H COSY spectra for compounds **2** can be seen in Figures S8–S14. Hence, compound **2** was elucidated as 3*β*, 12*β*, 20*S*-trihydroxy-25-hydroperoxydammar-23-ene-20-*O*-[*β*-d-glucopyranosyl (1→2)]-*β*-d-glucopyranoside, which was named as yunnangypenoside B.

Compound **3** was a white crystal, according to the adduct ion at *m*/*z* 845.4865 [M - H + HCOOH]^−^ (calcd for, 845.4899), its MF was determined to be C_42_H_72_O_14_, implying seven unsaturation degrees. The triterpenoid moiety was composed of 30 carbon signals, and the remaining 12 carbon atoms constituted two sugar moieties based on the ^13^C-NMR spectrum. Compound **3** yielded d-glucose based on the acid hydrolysis and HPLC analysis compared with the standard sugar, and the coupling constants (both 7.8 Hz) of anomeric protons indicated the *β* configuration for these d-glucopyranosyl units. The HMBC correlations between δ_H_ 5.16 (Glc’-1) and δ_C_ 84.4 (C-20), and between δ_H_ 5.65 (Glc’’-1) and δ_C_ 81.9 (Glc’-2) determined the two *β*-d-glucopyranosyl units located at C-20 and C-2’, respectively. The ^1^H-NMR data of **3** (Table 1) showed that it had eight methyls with chemical shifts at δ 0.94, 0.95, 0.96, 1.09, 1.29, 1.63, 1.64, and 1.66, and an olefinic proton signal at δ 5.26 (1H, t, *J* = 7.0 Hz). Two anomeric carbon signals at δ 97.1 (Glc’-1) and δ 105.8 (Glc’’-1), two olefinic carbons at δ 126.3 (C-24) and δ 131.1 (C-25), three oxygenated methine signals at δ 69.1 (C-2), δ 83.9 (C-3), and δ 71.0 (C-12), and an oxygenated quaternary carbon at δ 84.4 (C-20) were displayed in the ^13^C-NMR (Table 2) and DEPT-135 spectra. The HMBC spectrum showed correlations (Figure 2) between the following atoms: δ 0.96 (H3-18) and δ 35.3 (C-7), δ 40.4 (C-8), δ 51.9 (C-14); δ 0.94 (H_3_-19) and δ 50.5 (C-9), δ 48.5 (C-1), δ 56.7 (C-5), δ 38.9 (C-10); δ 1.64 (H_3_-21) and δ 84.4 (C-20), δ 53.1 (C-17), δ 36.6 (C-22); δ 1.66 (H_3_-27) and δ 126.3 (C-24), δ 131.1 (C-25), δ 26.1 (C-26); δ 1.29 (H_3_-28) and δ 83.9 (C-3), δ 56.7 (C-5), δ 17.7 (C-29), δ 40.2 (C-4); δ 1.09 (H_3_-29) and δ 83.9 (C-3), δ 56.7 (C-5), δ 29.6 (C-28), δ 40.2 (C-4); δ 0.95 (H_3_-30) and δ 40.4 (C-8), δ 51.9 (C-14), δ 31.1 (C-15); δ 69.1 (C-2) and 3.43 (H-3), 2.50 (H-1) 1.37 (H-1); δ 3.43 (H-3) and δ 17.7 (C-29), δ 29.6 (C-28), δ 69.1 (C-2). Through the HBMC key correlations, a dammaranne-type saponin with the same aglycone as that of gypenoside LXXIV [20] was suggested for compound **3**. The HR-ESI-MS, ^1^H-NMR, ^13^C-NMR, DEPT-135, HMBC, HSQC, and ^1^H-^1^H COSY spectra for compounds **3** can be seen in Figures S15–S21. Thus, the structure of **3** could be elucidated as 2*α*, 3*β*, 12*β*, 20*S*-tetrahydroxydammar-24-ene-20-*O*-[*β*-d-glucopyranosyl (1→2)]-*β*-d-glucopyranoside, which was named as yunnangypenoside C.

Compound **4** was a white powder, according to the adduct ion at *m*/*z* 1153.5916 [M - H + HCOOH]^−^ (calcd for, 1153.5947), its MF was determined to be C_54_H_92_O_23_, while its degree of unsaturation was nine. The spectral data were analyzed, and it was determined that compound **4** contained 54 carbons, including 30 carbon atoms belong to the aglycone, with the other 24 ones corresponding to four d-glucopyranosyl moieties. This indicated that the hydroxyl (C-3) in **3** was changed by two d-glucopyranosyl moieties in compound **4** based on the MS and NMR data. Through the coupling constants of anomeric protons, we could confirm that the four glycoside bonds were all *β* configuration. Those sugar units were linked to C-3, C-2’’’, C-20, and C-2’ based on the HMBC correlation from δ_H_ 4.97 (Glc-1’’’) to δ_C_ 89.1 (C-3), from δ_H_ 5.41 (Glc-1’’’’) to δ_C_ 83.6 (C-3), from δ_H_ 5.16 (Glc-1’) to δ_C_ 84.4 (C-20), and from δ_H_ 5.73 (Glc-1’’) to δ_C_ 81.2 (Glc-2’), respectively. In addition to the difference in sugar groups, there was also one hydroxyl group at C-2 of compound **4**. This suggested an olefinic proton signal at δ 5.23 and eight methyl proton signals at δ 0.78, 0.94, 1.00, 1.10, 1.32, 1.61, 1.62, and 1.65 in the ^1^H-NMR spectrum of **4** (Table 1). Moreover, four anomeric carbon signals at δ 105.5 (Glc-1’), δ 97.2 (Glc-1’’), δ 105.2 (Glc-1’’’), and δ 106.3 (Glc-1’’’’), two olefinic carbons at δ 126.3 (C-24) and δ 131.2 (C-25), two oxygenated methine signals at δ 89.1 (C-3) and δ 71.1 (C-12), and an oxygenated quaternary carbon at δ 84.4 (C-20) were proposed based on the ^13^C-NMR (Table 2) and DEPT-135 spectra. From the above data, we found that the aglycone part of compound **4** was similar to that of ginsenoside Rb_1_ [21]. This confirmed the above conjecture through the key correlations based on the HBMC spectrum (Figure 2). The HR-ESI-MS, ^1^H-NMR, ^13^C-NMR, DEPT-135, HMBC, HSQC, and ^1^H-^1^H COSY spectra for compounds **4** can be seen in Figures S22–S28. Therefore, compound **4** was determined as 3-*O*-[*β*-d-glucopyranosyl (1→2)]-*β*-d-glucopyranosyl-3*β*, 12*β*, 20*S*-trihydroxydammar-24-ene-20-*O*-[*β*-d-glucopyranosyl (1→2)]-*β*-d-glucopyranoside, and named as yunnangypenoside D.

Compound **5** was a white crystal, with an MF of C_56_H_94_O_24_, which was inferred from the adduct ion at *m*/*z* 1195.7152 [M - H + HCOOH]^−^ (calcd for, 1195.7144), showing 11 unsaturation degrees. Comparing the NMR spectra between **5** and **4** showed that the key difference was the acetyl group linked to the C-6’’’’ position in compound **5**, which was confirmed by the HMBC correlation from δ_H_ 4.01 (Glc’’’’-6) and δ_H_ 4.08 (Glc’’’’-5) to δ_C_ 171.3. The HR-ESI-MS and NMR data of **5** showed 56 carbon resonances, of which 30 belonged to the aglycone, 26 belonged to the sugar units, and one corresponded to an acetyl group. Acid hydrolysis of **5** gave four d-glucopyranosyl units according to analysis using the HPLC method. The *β* configuration was also determined based on the coupling constants (7.3, 7.9, 7.9, and 7.5 Hz). The HMBC cross-peaks from the anomeric protons between δ_H_ 4.95 (Glc-1’’’) and δ_C_ 89.3 (C-3), between δ_H_ 5.40 (Glc-1’’’’) and δ_C_ 83.7 (Glc-2’’’), between δ_H_ 5.27 (Glc-1’) and δ_C_ 84.1 (C-20), and between δ_H_ 5.29(Glc-1’’) and δ_C_ 84.9 (Glc-2’) indicated that the four d-glucopyranosyl units could be located at C-3, C-2’’’, C-20, and C-2’, respectively. The ^1^H-NMR data of **5** can be seen in Table 1, showing eight methyl proton signals at δ 0.85, 0.98, 1.09, 1.11, 1.32, 1.63, 1.66, and 1.67, an acetyl proton signal at δ 2.06 (3H, s), and an olefinic proton signal at δ 5.25 (1H, m). Four anomeric carbon signals at δ 97.1 (Glc-1’), δ 107.1 (Glc-1’’), δ 105.4 (Glc-1’’’), and δ 106.3 (Glc-1’’’’), two olefinic carbons at δ 126.2 (C-24) and δ 131.2 (C-25), three oxygenated methine signals at δ 27.2 (C-2), δ 89.3 (C-3), and δ 71.2 (C-12), and an oxygenated quaternary carbon at δ 84.1 (C-20) were displayed in the ^13^C-NMR (Table 2) and DEPT-135 spectra. Comparison of the NMR spectra of **5** with 6’’-*O*-acetylginsenoside Rb_1_ [22] indicated that they had the same aglycone moiety. We could further confirm our inference from the HMBC spectrum, for which the key correlations are shown in Figure 2. The HR-ESI-MS, ^1^H-NMR, ^13^C-NMR, DEPT-135, HMBC, HSQC, and ^1^H-^1^H COSY spectra for compounds **5** can be seen in Figures S29–S35. Thus, the structure of **5** could be elucidated as 3-*O*-{[*β*-d-[6-*O*-acetylglucopyranosyl] (1→2)}-*β*-d-glucopyranosyl-3*β*, 12*β*, 20(*S*)-trihydroxydammar-24-ene-20-*O*-[*β*-d-glucopyranosyl (1→2)]-*β*-d-glucpyranoside, and named as yunnangypenoside E.

Compound **6** was a light-yellow powder, and its MF was C_54_H_90_O_24_, which was inferred from the adduct ion at *m*/*z* 1167.6416 [M - H + HCOOH]^−^ (calcd for, 1167.6374). The NMR data of compound **6** were similar to those of **4**, except for the peaks related to the side chain. The data of C-25 (δ 144.9) and C-26 (δ 125.2) showed that there was a double bond at C-25 (26) and a carbonyl group at C-24 in **6**. The data showed that the aglycone portion of compound **6** included 30 carbons, and the remaining 24 carbon signals were assigned to four sugar moieties. The NMR spectra showed that the configuration of the four sugar groups was *β* configuration. The HMBC data showed correlations between δ_H_ 4.90 (Glc-1’’’) and δ_C_ 89.1 (C-3), between δ_H_ 5.40 (Glc-1’’’’) and δ_C_ 83.6 (Glc-2’’’), between δ_H_ 5.10 (Glc-1’) and δ_C_ 84.2 (C-20), and between δ_H_ 5.82 (Glc-1’’) and δ_C_ 79.8 (Glc-2’), indicating that the four sugars could be located at C-3, C-2’’’, C-20, and C-2’, respectively. The one-dimensional (1D) NMR and HMBC (Figure 2) spectra demonstrated that **6** possessed the same dammarane-type triterpene skeleton as that of notoginsenoside-B [23]. The HR-ESI-MS, ^1^H-NMR, ^13^C-NMR, DEPT-135, HMBC, HSQC, and ^1^H-^1^H COSY spectra for compounds **6** can be seen in Figures S36–S42. Thus, the structure of **6** was assigned as 3-*O*-[*β*-glucopyranosy (1→2)-glucopyranosyl]-20-*O*-[*β*-glucopyranosy (1→2)-*β*-glucopyranosyl]-3*β*, 12*β*, 20(*S*)-trihydroxydammar-25-ene-24-one, which was named as yunnangypenoside F.

Compound **7** was a white powder, and its MF of C_54_H_92_O_22_ was inferred from the HR-ESI-MS data. The NMR spectroscopic properties of **7** and **4** were similar, with the differences being the sugar unit and the absence of the hydroxyl (C-12) in **7**. Through analysis of the NMR data, 54 carbons were obtained, of which 30 were allocated to the triterpene skeleton. Acid hydrolysis of **7** gave four d-glucopyranosyl units according to analysis using the HPLC method. The *β* configuration was also determined based on the coupling constants (7.6, 7.6, 7.6, and 7.4 Hz). The HMBC cross-peaks from the anomeric protons δ_H_ 4.96 (Glc-1’) to δ_C_ 89.4 (C-3), δ_H_ 5.13 (Glc-1’’) to δ_C_ 83.5 (C-20), δ_H_ 5.38 (Glc-1’’’) to δ_C_ 83.7 (C-2’’), and δ_H_ 5.36 (Glc-1’’’’) to δ_C_ 83.7 (C-3’’) suggested the locations of those sugar units at C-3, C-20, C-2’’, and C-3’’, respectively. Some characteristic carbon and hydrogen symbols were proposed from the ^13^C-NMR (Table 3) and DEPT-135 spectra. The skeleton of compound **7** was suggested based on the NMR spectral data, and the basic mother nucleus of **7** was the same as that of vina-ginsenoside-R_3_ [24]. This confirmed the above conjecture through key correlations based on the HBMC spectrum (Figure 2). The HR-ESI-MS, ^1^H-NMR, ^13^C-NMR, DEPT-135, HMBC, HSQC, and ^1^H-^1^H COSY spectra for compounds **7** can be seen in Figures S43–S49. Thus, compound **7** could be elucidated as 3*β*, 20(*S*)-dihydroxydammar-24-ene-3-*O*-[*β*-d-glucopyranosyl]-20-*O*-[*β*-d-glucopyranosyl (1→2)] [*β*-d-glucopyranosyl (1→3)]-*β*-d-glucopyranosyl, and named as yunnangypenoside G.

Compound **8** was a light-yellow powder, with its MF of C_54_H_92_O_22_ inferred from the adduct ion. Comparing the NMR spectra between **8** and **4** showed that the key difference was the sugar unit. Acid hydrolysis of **8** gave three *β*-d-glucopyranosyls and one *α*-l-rhamnopyranosyl unit based on analysis using the HPLC method and the NMR spectra. The HMBC correlations from δ_H_ 4.96 (Glc-1’) to δ_C_ 89.1 (C-3), from δ_H_ 5.17 (Glc-1’’) to δ_C_ 84.4 (C-20), from δ_H_ 5.64 (Glc-1’’’) to δ_C_ 81.7 (C-2’’), and from δ_H_ 6.56 (Rha-1’’’’) to δ_C_ 78.3 (C-2’’’) determined that the three *β*-d-glucopyranosyl units were located at C-3, C-20, and C-2’’, while the *α*-L-rhamnopyranosyl unit was located at C-2’’’, respectively. The NMR data of **8** can be seen in Table 4. Comparison of the NMR spectrum of **8** with that of (3*β*, 12*β*, 20*S*)-trihydroxydammar-24-ene-3-*O*-*β*-d-glucopyranosyl-20-*O*-[*α*-rhamnopyranosyl-(1→2)] [*α*-rhamnopyranosyl-(1→3)]-*β*-glucopyranoside [25] indicated that they had the same aglycone moiety. Furthermore, the HMBC data provided the key correlations in Figure 2, which confirmed the above speculative results. The HR-ESI-MS, ^1^H-NMR, ^13^C-NMR, DEPT-135, HMBC, HSQC, and ^1^H-^1^H COSY spectra for compounds **8** can be seen in Figures S50–S56. Thus, the structure of **8** could be elucidated as (3*β*, 12*β*, 20*S*)-trihydroxydammar-24-ene-3-*O*-*β*-d-glucopyranosyl-20-*O*-[*α*-l-rhamnopyranosyl-(1→2)-*β*-d-glucopyranosyl]-(1→2)-*β*-glucopyranoside, which was named as yunnangypenoside H.

Compound **9** was a light-yellow powder, and the MF of C_48_H_82_O_18_ was inferred from the adduct ion at *m*/*z* 991.5416 [M - H+HCOOH]^−^ (calcd for, 991.5419). The NMR, HMBC, and HR-ESI-MS spectroscopic data of **9** could be differentiated from those of **8** by the lack of the rhamnopyranosyl unit. The *β* configuration for these glucopyranosyl units was identified by the coupling patterns of the anomeric proton signals (δ 4.96, 5.16, and 5.73) with the same coupling constant (7.8 Hz). The HMBC correlations from δ_H_ 5.16 (Glc-1’) to δ_C_ 84.4 (C-20), from δ_H_ 5.73 (Glc-1’’) to δ_C_ 81.1 (C-2’), and from δ_H_ 4.96 (Glc-1’’’) to δ_C_ 89.0 (C-3) suggested that the glucopyranosyl units were located at C-20, C-2’, and C-3, respectively. The skeleton of compound **9** was suggested based on the ^1^H-NMR (Table 1), ^13^C-NMR (Table 2), and DEPT-135 spectral data, revealing that the basic mother nucleus of **9** was the same as that of ginsenoside Rd [26], which was also confirmed through the key correlations based on the HMBC spectrum (Figure 2). The HR-ESI-MS, ^1^H-NMR, ^13^C-NMR, DEPT-135, HMBC, HSQC, and ^1^H-^1^H COSY spectra for compounds **9** can be seen in Figures S57–S63. Consequently, compound **9** was deduced to be 3*β*, 12*β*, 20(*S*)-trihydroxydammar-24-ene-3-*O*-[*β*-glucopyranosyl]-20-*O*-[*β*-glucopyranosyl (1→2)]-*β*-glucopyranoside, which was named as yunnangypenoside I.

Compound **10** was a light-yellow powder, and the MF of C_54_H_92_O_24_ was inferred from the adduct ion at *m*/*z* 1169.5951 [M - H + HCOOH]^−^ (calcd for, 1169.5955). Upon comparing the NMR data of **10** and **3**, we found that the major difference was the sugar unit. The NMR data showed that **10** was composed of four units of glucopyranosyl, and the configuration of the sugar units was *β*-linked. The locations of the sugar units were determined at C-3, C-20, C-2’’, and C-2’’’ positions based on the observed HMBC correlations between δ_H_ 4.95 (Glc-1’) and δ_C_ 95.7 (C-3), between δ_H_ 5.18 (Glc-1’’) and δ_C_ 84.4 (C-20), between δ_H_ 5.65 (Glc-1’’’) and δ_C_ 81.9 (C-2’’), and between δ_H_ 5.52 (Glc-1’’’’) and δ_C_ 82.6 (C-2’’’), respectively. Comparison of the NMR spectrum (Table 5) of **10** with 2*α*, 3*β*, 12*β*, 20*S*-tetrahydroxydammar-24-ene-3-*O*-*β*-sophoroisde-20-*O*-*β*-gentiobioside [27] indicated that they had the same aglycone moiety. We could further confirm our inference from the HMBC spectrum, with the key correlations shown in Figure 2. The HR-ESI-MS, ^1^H-NMR, ^13^C-NMR, DEPT-135, HMBC, HSQC, and ^1^H-^1^H COSY spectra for compounds **10** can be seen in Figures S64–S70. Thus, the structure of **10** could be elucidated as 2*α*, 3*β*, 12*β*, 20*S*-tetrahydroxydammar-24-ene-3-*O*-*β*-glucopyranosyl-20-*O*-[*β*-glucopyranosyl(1→2)]-*β*-glucopyranosyl]-(1→2)-*β*-glucopyranoside, which was named as yunnangypenoside J.

### 2.2. Bioactivity Evaluation

The *G. pentaphyllum* is a functional herbal tea, which has a lot of biological effects. In our study, the lipid-lowering activity of 11 components on human HepG-2 hepatocellular carcinoma cells was determined.

#### 2.2.1. Cytotoxic Activity Assay

**Figure 3:**
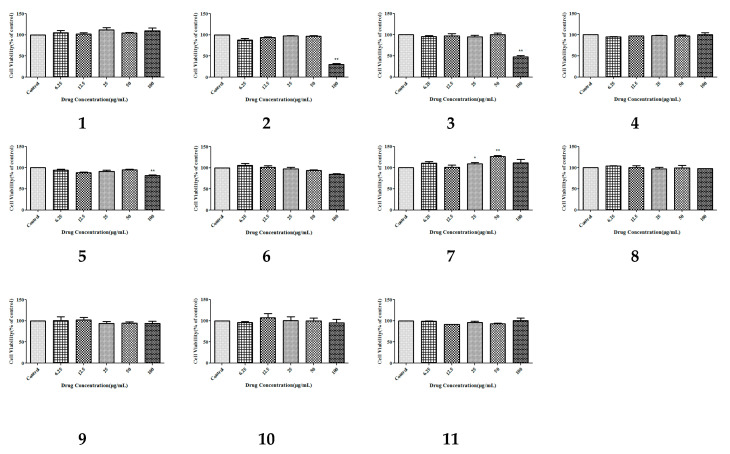
The effect of the isolated compounds on the survival rate of HepG-2 human hepatocellular carcinoma cells. The cells were pretreated with concentrations (6.25, 12.5, 25, 50, and 100 µg/mL) of compounds **1**–**11** for 1 h. The data show the mean ± SD of three independent experiments performed in triplicates.

The CCK-8 assay provides a convenient and robust way of determining cell viability, which uses water-soluble tetrazolium salt (WST-8) to produce an orange formazan dye upon bio-reduction in the presence of an electron carrier by dehydrogenases. Cytotoxicity screening on the isolated compounds **1**–**11** was measured using the CCK-8 assay in HepG-2 cells. The results (Figure 3) of activity experiments showed that these compounds had no significant cytotoxicity at concentrations from 6.25 µg/mL to 100 µg/mL. These cytotoxic results could tell us that, at the appropriate concentration, these compounds were almost non-cytotoxic; so, there was no toxic effect seen during our determination of hypolipidemic activity.

#### 2.2.2. Hypolipidemia Activity Assay

**Figure 4:**
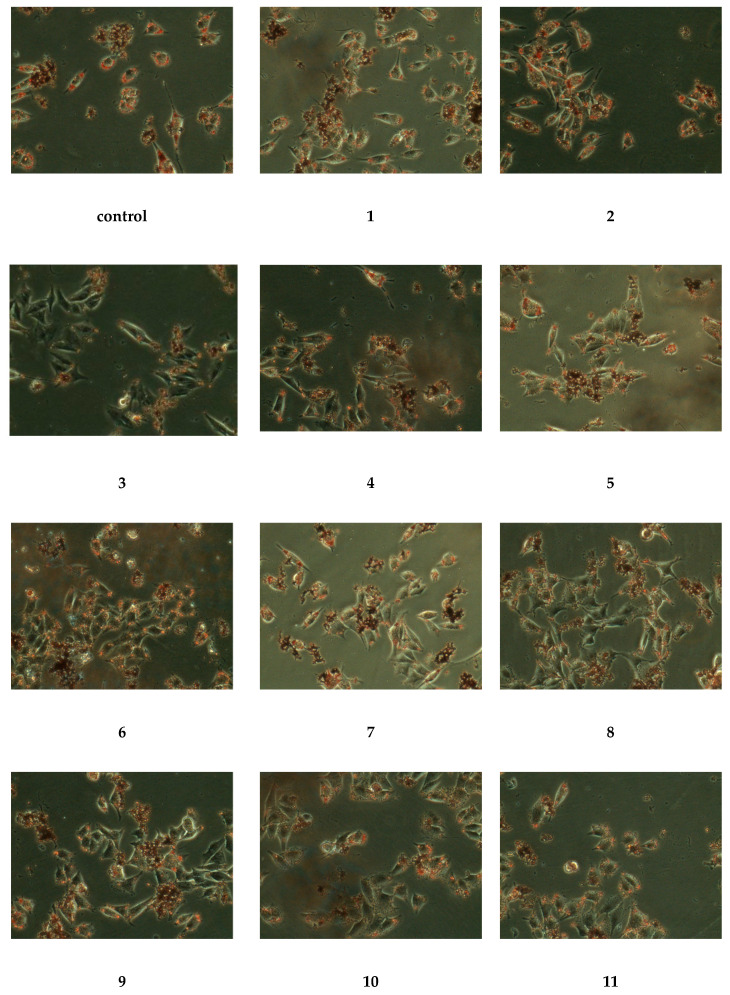
Lipid lowering effects of the isolated compounds in HepG-2 human hepatocellular carcinoma cells. The cells were pretreated with concentration (50 µg/mL) of compounds **1**–**11** for 24 h and then stained with oil red O for 1 h in the dark environment.

To evaluate the inhibitory effects of the isolates on oleic acid-induced lipid accumulation, HepG-2 cells were treated with compounds **1**–**11** (50 µg/mL) in the presence of oleic acid for 24 h. The results were displayed in Figure 4, and HepG-2 cells in the control group contained numerous red lipid droplets fused each other, indicating that the HepG-2 cell lipid accumulation model was set up successfully. The lipid drops in cells treated with compounds **1**, **3**–**11** had light colors and smaller volumes, while compound **8** was the most obvious by comparison with the control group. Through the above phenomena, we could conclude that compounds **1**, **3**–**11** had strong antilipidemic activity, while compound **8** had the strongest antilipidemic activity.

## 3. Materials and Methods

### 3.1. General Experimental Procedures

The high-resolution electrospray ionization mass spectroscopy (HR-ESI-MS) (Waters, Milford, CT, USA) data were detected using an electrostatic field orbital trap mass spectrometer (Waters) with an ESI source. We measured the NMR spectra of all isolated compounds on an AV-400 spectrometer (Bruker, Faellanden, Switzerland) using trimethylsilane (TMS) as an internal standard (IS). Semi-Pr-HPLC (C_18_ column, 250 mm × 10 mm, 5 µm, Cosmosil, Tokyo, Japan) was performed using a Waters 2489 instrument. Analytical HPLC (SunFire RP C_18_) was carried out on an Agilent 1260 instrument (Agilent Technologies, Palo Alto, CA, USA). Ostade-cylsilane (ODS) gel (5–50 µm, Dameng Technology, Chengdu, China), Sephadex LH-20 (Feixiang Biotechnology Co., Ltd., Najing, China), and silica gel (Yantai ocean chemicals, Yantai, China) were used for CC. All other chemical reagents were purchased from Tangshan Xiangyu Technology and Tianjin Lantian Biomedical Technology.

### 3.2. Plant Material

We collected the plants of *G. pentaphyllum* from Yunnan province, China, in October 2016, which were identified by Chunhua Wang, Tianjin University of Traditional Chinese Medicine. One sample (No. TJTCMBH20161006CH) was kept at the College of Pharmaceutical Engineering of TCM, Poyanghu Road, Jinghai, Tianjin, China.

### 3.3. Extraction and Isolation

**Figure 5:**
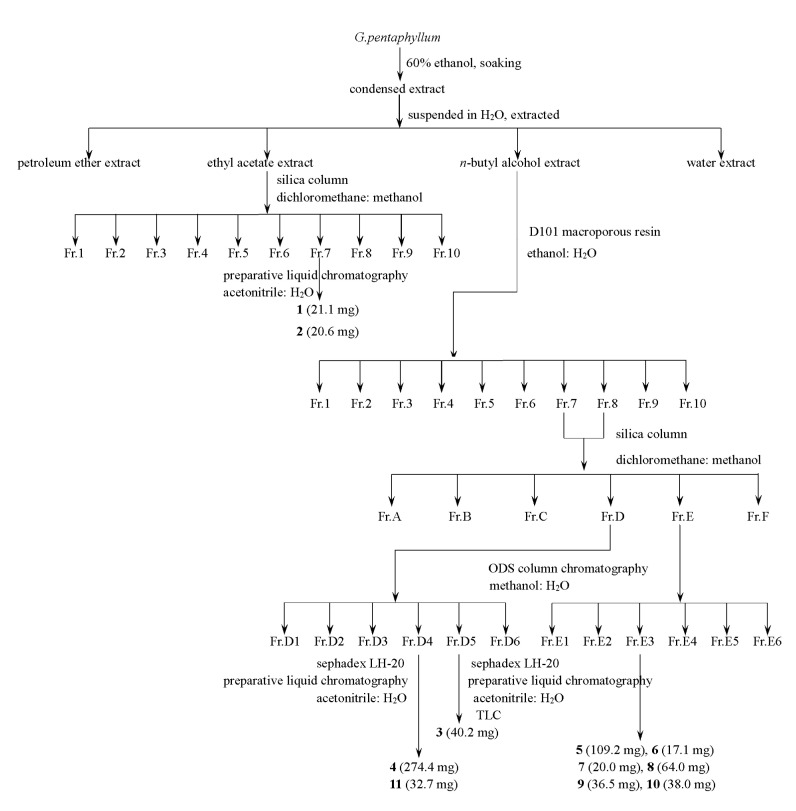
Scheme illustrating the entire isolation process.

We dried the plants at 60 °C in the oven; next, the processed herbs of *G. pentaphyllum* (about 5000 g) were soaked in 60% C_2_H_5_OH/H_2_O solution (*v*/*v*) for three days, three times in total, and then filtered. The extracted solution was mixed and rotated to evaporate until no alcohol was present. We suspended the extract with water, and then extracted it with three different polar solvents (petroleum ether (PE) three times, ethyl acetate (EtOAc) five times, and five times with *n*-butanol). After this step, the EtOAc extract was analyzed using silica gel column chromatography (*v*/*v*, MeOH: CH_2_Cl_2_, 0–75%, gradient elution), giving 10 fractions (Fr.1–Fr.10). Fr.7 was chromatographed using Sephadex LH-20 and RP-HPLC (*v*/*v*, acetonitrile: H_2_O, 0–100%, stepwise) to yield 21.1 mg of **1**, along with 20.6 mg of **2**. Then, the *n*-butyl alcohol extract was chromatographed over D-101 macroporous resin (*v*/*v*, ethanol: H_2_O) to get the 70% ethanol eluate; after drying, 43.46 g of saponins were obtained. The saponin part was isolated using silica gel CC (*v*/*v*, MeOH: CH_2_Cl_2_, 0–80%, gradient elution) to give 10 fractions (Fr.1–Fr.10). After combining Fr.7 with Fr.8, they were separated into six fractions (Fr.A–Fr.F) through chromatography over silica gel (*v*/*v*, CH_3_OH: CH_2_Cl_2_, 0–100%, gradient concentration elution). Fr.D and Fr.E were subjected to RP 18 CC (*v*/*v*, CH_3_OH: H_2_O, 0–100%, stepwise) to yield six fractions named Fr.D1–Fr.D6 and Fr.E1–Fr.E6, respectively. Fr.D4 was isolated using RP-HPLC (*v*/*v*, acetonitrile: H_2_O) and Sephadex LH-20 gel to yield 274.4 mg of **4** and 32.4 mg of **11**. Compound **3** (40.2 mg) was obtained via RP-HPLC (*v*/*v*, acetonitrile: H_2_O), Sephadex LH-20 gel, and thin layer chromatography (TLC) of Fr.D5. Fr.F4 isolated through CC over Sephadex LH-20 gel, RP-HPLC (*v*/*v*, acetonitrile: H_2_O), and TLC to yield 109.2 mg of **5**, 17.1 mg of **6**, 20.0 mg of **7**, 64.0 mg of **8**, 36.5 mg of **9**, and 38.0 mg of **10** (see Figure 5).

#### 3.3.1. Yunnangypenoside A (**1**)

Light yellow powder (MeOH); for ^1^H nuclear magnetic resonance (NMR) (600 MHz, pyridine-*d*_5_) and ^13^C-NMR (150 MHz, pyridine-*d*_5_) data, see Table 1 and Table 2; high-resolution electrospray ionization mass spectroscopy (HR-ESI-MS) *m*/*z* 861.4809 [M − H + HCOOH]^−^ (calcd for C_43_H_73_O_17_, 861.4848).

#### 3.3.2. Yunnangypenoside B (**2**)

White powder (MeOH); for ^1^H-NMR (600 MHz, pyridine-*d*_5_) and ^13^C-NMR (150 MHz, pyridine-*d*_5_) data, see Table 1 and Table 2; HR-ESI-MS *m*/*z* 861.4811 [M − H + HCOOH]^−^ (calcd for C_43_H_73_O_17_, 861.4848).

#### 3.3.3. Yunnangypenoside C (**3**)

White crystals (MeOH); for ^1^H-NMR (600 MHz, pyridine-*d*_5_) and ^13^C-NMR (150 MHz, pyridine-*d*_5_) data, see Table 1 and Table 2; HR-ESI-MS *m*/*z* 845.4865 [M − H + HCOOH]^−^ (calcd for C_43_H_73_O_16_, 845.4899).

#### 3.3.4. Yunnangypenoside D (**4**)

White powder (MeOH); for ^1^H-NMR (600 MHz, pyridine-*d*_5_) and ^13^C-NMR (150 MHz, pyridine-*d*_5_) data, see Table 1 and Table 2; HR-ESI-MS *m*/*z* 1153.5916 [M − H + HCOOH]^−^ (calcd for C_55_H_93_O_25_, 1153.5947).

#### 3.3.5. Yunnangypenoside E (**5**)

White crystals (MeOH); for ^1^H-NMR (600 MHz, pyridine-*d*_5_) and ^13^C-NMR (150 MHz, pyridine-*d*_5_) data, see Table 1 and Table 2; HR-ESI-MS *m*/*z* 1195.7152 [M − H + HCOOH]^−^ (calcd for C_57_H_95_O_26_, 1195.7144).

#### 3.3.6. Yunnangypenoside F (**6**)

Light-yellow powder (MeOH); for ^1^H-NMR (600 MHz, pyridine-*d*_5_) and ^13^C-NMR (150 MHz, pyridine-*d*_5_) data, see Table 1 and Table 2; HR-ESI-MS *m*/*z* 1167.6416 [M − H + HCOOH]^−^ (calcd for C_55_H_91_O_26_, 1167.6374).

#### 3.3.7. Yunnangypenoside G (**7**)

White powder (MeOH); for ^1^H-NMR (600 MHz, pyridine-*d*_5_) and ^13^C-NMR (150 MHz, pyridine-*d*_5_) data, see Table 3; HR-ESI-MS *m*/*z* 1137.6077 [M − H + HCOOH]^−^ (calcd for C_55_H_93_O_24_, 1137.6116).

#### 3.3.8. Yunnangypenoside H (**8**)

Light-yellow powder (MeOH); for ^1^H-NMR (600 MHz, pyridine-*d*_5_) and ^13^C-NMR (150 MHz, pyridine-*d*_5_) data, see Table 4; HR-ESI-MS *m*/*z* 1137.6040 [M − H + HCOOH]^−^ (calcd for C_55_H_93_O_24_, 1137.6057).

#### 3.3.9. Yunnangypenoside I (**9**)

Light-yellow powder (MeOH); for ^1^H-NMR (600 MHz, pyridine-*d*_5_) and ^13^C-NMR (150 MHz, pyridine-*d*_5_) data, see Table 1 and Table 2; HR-ESI-MS *m*/*z* 991.5416 [M − H + HCOOH]^−^ (calcd for C_49_H_83_O_20_, 991.5419).

#### 3.3.10. Yunnangypenoside J (**10**)

Light-yellow powder (MeOH); for ^1^H-NMR (600 MHz, pyridine-*d*_5_)and ^13^C-NMR (150 MHz, pyridine-*d*_5_) data, see Table 5; HR-ESI-MS *m*/*z* 1169.5951 [M − H + HCOOH]^−^ (calcd for C_55_H_93_O_26_, 1169.5955).

### 3.4. Acid Hydrolysis of Dammarane-Type Glycosides

The sugar parts of compounds **1**–**5**, **8** were obtained using acid hydrolysis and HPLC analysis. This approach was based on methods published in the literature [28]. Each isolated compound (4 mg) was treated with 2 mol/L HCl (2 mL) under reflux conditions at 85 °C for 2 h. Each mixture was extracted with ethyl acetate to afford the aglycone portion, and the aqueous layer was desiccated under reduced pressure. Then, pyridine (1 mL), l-cysteine methyl ester (4 mg), and *O*-tolyl isothiocyanate (4 mg) were added to the evaporated filtrate in sequence, and the mixture was stirred at 60 °C for 1 h. Each derivative fraction was subjected to HPLC (LC, column, Symmetry Shield ^TM^RP C18; column temperature, 35 °C; mobile phase, 25% acetonitrile contained 0.1% formic acid; flow rate, 0.8 mL/min; ultraviolet detection wavelength, 254 nm). Under these conditions, the sugars of each reactant were identified by comparison with authentic standard derivatives (d-glucose, l-glucose, and l-rhamnose).

### 3.5. Cytotoxic Bioactivity

#### 3.5.1. Cell Culture and Reagents

HepG-2 cells were obtained from the Binhai Lab of Tianjin University of Traditional Chinese Medicine (Bio-Swamp, MD, USA). The culture process was derived from the literature [29,30].

#### 3.5.2. Cell Viability Assays

The CCK-8 test was used to evaluate the cytotoxic activities of these isolated natural products. The measurements and statistical methods were carried in reference to the literature [28,31].

#### 3.5.3. Hypolipidemia Activity Assay

We tested the lipid-lowering bioactivities of 11 compounds using the oil red O staining method. The cells were grown on six-well plates, where each well (500 µL) contained 2.5 × 10^5^ cells, which were incubated for 24 h. After the culture medium was substituted by medium with the 11 compounds (50 µL/mL), oleic acid (0.5 mmol/L) was added [27]. Cells were washed three times with phosphate buffer saline (PBS) after 24 h. Then, the cells were fixed with 4% paraformaldehyde (1 mL) for 30 min. Then, they were rewashed with PBS, and the cells were infiltrated with 60% isopropyl alcohol (1 mL) for 10 s. Finally, the cells were stained using oil red O for 1 h in a dark environment. For optical microscopy observation, the cells were washed three times with PBS [32,33].

#### 3.5.4. Statistics

The *t*-test was used to analyze the differences between groups of data, with the significance of difference among groups determined using SPSS version 17.0 (International Business Machines Corporation, Armonk, NY, USA) and statistical diagrams generated using GraphPad Prism version 5.0 (GraphPad Software Inc., La Jolla, CA, USA). A *p*-value less than 0.05 (*p < 0.05) was considered statistically significant. A *p*-value less than 0.01 (**p < 0.01) denoted notable statistical significance.

## 4. Conclusions

*G. pentaphyllum*, as a kind of functional tea beverage commonly used by people, attracted the interest of our research group due to its lipid-lowering chemical activity. As far as we know, *G. pentaphyllum* is becoming more and more popular as a food and beverage. Therefore, much chemical analysis work was carried out for *G. pentaphyllum*, and researchers found a large number of *Gynostemma* saponins. However, no detailed chemical composition report was found on the plants we picked in Yunnan province, and some unknown antilipidemic active ingredients were not clarified. Based on the above reasons, we collected some samples from Yunnan Province, China, and carried out a systematic chemical separation with ethanol extract using the method of food chemical analysis. Interestingly, 10 previously undescribed dammaranne-type saponins (**1**–**10**) and one known compound (**11**) were obtained. To study the antilipidemic activity of these compounds, an oil red O staining assay was carried out to determine their bioactivities. Interestingly, these isolated compounds produced hypolipidemia activity in HepG-2 cells except for **2**, while compound **8** exhibited the best hypolipidemia activity through the oil red O staining assay. This study provides some scientific evidence for people drinking *G. pentaphyllum* tea to reduce blood lipid levels, while it also provides new compounds which can be used to enrich the chemical composition of this functional herbal tea. We believe that our research will encourage further studies of the chemical composition and antilipidemic activity of *G. pentaphyllum*, leading to the development of a healthy tea food based on *G. pentaphyllum*.

## Figures and Tables

**Table 1:** ^1^H-NMR data for compounds **1**–**6**, **9** (600 MHz, pyridine-*d*_5_, δ in ppm, *J* in Hz).

No.	1	2	3	4	5	6	9
1	1.81 (m),1.07 (m)	1.81 (m), 1.05 (m)	2.50 (m),1.37 (m)	1.61 (m), 0.83 (m)	1.57 (m), 0.86 (m)	1.62 (m), 0.84 (m)	1.62 (m), 0.88 (m)
2	1.79 (m), 1.67 (m)	1.83 (m), 1.74 (m)	4.11 (m)	2.06 (m), 1.76 (m)	2.23 (m), 2.12 (m)	2.03 (m), 1.75 (m)	1.95 (m), 1.39 (m)
3	3.41 (m)	3.43 (dd, 11.4, 4.4)	3.43 (d, 9.4)	3.25 (dd, 11.7, 4.6)	3.33 (dd, 11.7, 4.5)	3.22 (dd, 11.7, 4.6)	3.35 (dd, 11.8, 4.5)
4	-	-	-	-	-	-	-
5	0.83 (m)	0.85 (m)	0.98 (m)	0.66 (m)	0.73 (m)	0.63 (m)	0.72 (m)
6	1.58 (m), 1.46 (m)	1.61 (m), 1.50 (m)	1.57 (m), 1.48 (m)	1.49 (m), 1.36 (m)	1.51 (m), 1.38 (m)	1.66 (m), 1.47 (m)	1.50 (m), 1.37 (m),
7	1.48 (m), 1.23 (m)	1.51 (m), 1.26 (m)	1.49 (m), 1.24 (m)	1.42 (m), 1.20 (m)	1.48 (m), 1.23 (m)	1.39 (m), 1.18 (m)	1.46 (m), 1.20 (m)
8	-	-	-	-	-	-	-
9	1.51 (m)	1.54 (m)	1.59 (m)	1.43 (m)	1.44 (m)	1.42 (m)	1.45 (m)
10	-	-	-	-	-	-	-
11	2.70 (m), 1.30 (m)	1.61 (m), 1.01 (m)	1.58 (m), 1.02 (m)	2.19 (m), 1.43 (m)	2.04 (m), 1.45 (m)	1.57 (m), 0.99 (m)	1.57 (m), 1.01 (m)
12	4.17 (m)	4.12 (m)	4.12 (m)	4.17 (m)	3.98 (m)	4.18 (m)	4.16 (m)
13	2.05 (m)	2.03 (m)	2.00 (m)	2.00 (m)	2.00 (m)	2.04 (m)	1.99 (m)
14	-	-	-	-	-	-	-
15	2.30 (m), 1.52 (m)	2.28 (m), 1.55 (m)	2.35 (m), 1.34 (m)	1.57 (m), 1.01 (m)	1.57 (m), 1.08 (m)	2.20 (m), 1.42 (m)	2.20 (m), 1.45 (m)
16	2.22 (m), 2.12 (m)	1.92 (m), 1.47 (m)	1.99 (m), 1.41 (m)	1.97 (m), 1.40 (m)	1.86 (m), 1.55 (m)	1.93 (m), 1.44 (m)	1.62 (m), 1.59 (m)
17	2.54 (m)	2.57 (m)	2.57 (m)	2.56 (m)	2.62 (m)	2.53 (m)	2.56 (m)
18	0.97 (s)	1.05 (s)	0.96 (s)	0.94 (s)	0.98 (s)	0.93 (s)	0.93 (s)
19	0.87 (s)	0.93 (s)	0.94 (s)	0.78 (s)	0.85 (s)	0.76 (s)	0.78 (s)
20	-	-	-	-	-	-	-
21	1.62 (s)	1.60 (s)	1.64 (s)	1.62 (s)	1.66 (s)	1.54 (s)	1.60 (s)
22	2.47 (m), 2.21 (m)	3.08 (m), 2.69(m)	2.40 (m), 1.89 (m)	2.38 (m), 1.86 (m)	2.35 (m), 2.03 (m)	2.66 (m), 2.17 (m)	2.37 (m), 1.85 (m)
23	1.90 (m), 1.38 (m)	6.23 (m),	2.47 (m), 2.29 (m)	2.50 (m), 2.28 (m)	2.35 (m)	3.45 (m), 3.17 (m)	2.48 (m), 2.25 (m)
24	4.69 (d, 8.1, 4.9)	6.02 (d, 15.8)	5.26 (t, 7.0)	5.23 (t, 7.0)	5.25 (m)	-	5.21 (m)
25	-	-	-	-	-	-	-
26	5.23 (s), 5.07 (m)	1.62 (s)	1.63 (s)	1.61 (s),	1.63 (s)	6.34 (s), 5.74 (s)	1.62 (s)
27	1.96 (s)	1.62 (s)	1.66 (s)	1.65 (s)	1.67 (s)	1.90 (s)	1.63 (s)
28	1.25 (s)	1.26 (s)	1.29 (s)	1.32 (s)	1.32 (s)	1.31 (s)	1.34 (s)
29	1.03 (s)	0.95 (s)	1.09 (s)	1.10 (s)	1.11 (s)	1.09 (s)	1.00 (s)
30	0.93 (s)	0.96 (s)	0.95 (s)	1.00 (s)	1.09 (s)	0.96 (s)	1.00 (s)
20-*O*-Glc							
1’	5.12 (d, 7.8)	5.18 (d, 7.8)	5.16 (d, 7.8)	5.16 (d, 7.7)	5.27 (d, 7.9)	5.10 (br, s)	5.16 (d, 7.8)
2’	4.28 (m)	4.25 (m)	4.24 (m)	4.28 (m)	4.10 (m)	4.31 (m)	4.27 (m)
3’	3.88 (m)	4.29 (m)	4.40 (m)	4.36 (m)	4.28 (m)	4.29 (m)	4.00 (m)
4’	4.15 (m)	4.10 (m)	4.31 (m)	4.34 (m)	4.17 (m)	4.32 (m)	4.28 (m)
5’	4.35 (m)	3.94 (m)	3.94 (m)	4.28 (m)	4.28 (m)	4.37 (m)	4.00 (m)
6’	4.43 (m)	4.51 (m)	4.45 (m)	4.48 (m)	4.29 (m)	4.72 (m)	4.30 (m)
2’-*O*-Glc							
1’’	5.80 (d, 7.8)	5.67 (d, 7.8)	5.65 (d, 7.8)	5.73 (d, 7.7)	5.29 (d, 7.9)	5.82 (d, 7.8)	5.73 (d, 7.8)
2’’	4.14 (m)	4.13 (m)	4.15 (m)	4.14 (m)	4.14 (m)	4.13 (m)	4.05 (m)
3’’	3.82 (m)	4.30 (m)	3.85 (m)	4.28 (m)	3.94 (m)	4.29 (m)	4.37 (m)
4’’	4.31 (m)	4.29 (m)	4.22 (m)	4.17 (m)	4.35 (m)	4.62 (m)	4.27 (m)
5’’	4.30 (m)	3.94 (m)	4.31 (m)	4.29 (m)	3.85 (m)	4.29 (m)	3.85 (m)
6’’	4.28 (m)	4.30 (m)	4.32 (m)	4.31 (m)	4.38 (m)	4.49 (m)	4.55 (m)
3-*O*-Glc							
1’’’	-	-	-	4.93 (d, 7.6)	4.95 (d, 7.3)	4.90 (d, 7.7)	4.96 (d, 7.8)
2’’’	-	-	-	4.25 (m)	4.26 (m)	4.24 (m)	4.14 (m)
3’’’	-	-	-	4.30 (m)	3.85 (m)	4.32 (m)	4.28 (m)
4’’’	-	-	-	4.18 (m)	4.35 (m)	3.91 (m)	4.27 (m)
5’’’	-	-	-	3.86 (m)	4.28 (m)	4.29 (m)	3.92 (m)
6’’’	-	-	-	4.49 (m)	4.50 (m)	4.49 (m)	4.45 (m)
2’’’-*O*-Glc							
1’’’’	-	-	-	5.41 (d, 7.6)	5.40 (d, 7.5)	5.40 (d, 7.7)	-
2’’’’	-	-	-	4.15 (m)	4.14 (m)	4.13 (m)	-
3’’’’	-	-	-	4.28 (m)	3.85 (m)	3.91 (m)	-
4’’’’	-	-	-	4.18 (m)	4.17 (m)	4.12 (m)	-
5’’’’	-	-	-	3.93 (m)	4.08 (m)	3.91 (m)	-
6’’’’	-	-	-	4.39 (m)	5.01 (m)	4.26 (m)	-
6’’’’-OCOCH_3_	-	-	-	-	-	-	-
CH_3_COO	-	-	-	-	2.06 (s)	-	-

**Table 2:** ^13^C-NMR data for compounds **1**–**6**, **9** (150 MHz, pyridine-*d*_5_, δ in ppm).

No.	1	2	3	4	5	6	9
1	39.9	39.7	48.5	39.3	39.5	39.3	39.3
2	28.4	28.5	69.1	26.9	27.2	26.8	27.4
3	78.3	78.3	83.9	89.1	89.3	89.1	89.0
4	39.7	39.9	40.2	40.0	40.1	40.0	40.0
5	56.5	56.6	56.7	56.6	56.7	56.5	56.6
6	19.1	19.1	19.1	18.8	18.8	18.8	18.8
7	35.4	35.5	35.3	35.4	35.4	35.3	35.3
8	40.3	40.4	40.4	40.3	40.3	40.3	40.3
9	50.6	50.6	50.5	50.4	50.3	50.5	50.4
10	37.7	37.7	38.9	37.3	37.3	37.3	37.3
11	30.3	31.1	31.2	30.9	31.5	31.2	31.2
12	71.0	71.2	71.0	71.1	71.2	71.1	71.1
13	49.5	49.7	49.6	49.5	49.6	49.4	49.5
14	51.9	52.0	51.9	52.0	52.0	52.0	52.0
15	30.8	31.0	31.1	31.3	31.3	30.7	30.9
16	27.0	27.1	27.2	27.4	27.1	27.5	26.9
17	52.9	52.9	53.1	53.0	53.6	53.3	53.0
18	16.3	16.3	16.3	16.2	16.1	16.3	16.2
19	16.6	16.7	17.9	16.6	16.6	16.6	16.6
20	84.5	84.1	84.4	84.4	84.1	84.2	84.4
21	22.3	22.9	22.2	22.4	22.9	22.0	22.3
22	33.2	40.2	36.6	36.6	36.3	29.9	36.6
23	27.2	126.9	23.8	23.8	24.3	33.3	23.8
24	90.3	138.4	126.3	126.3	126.2	202.6	126.3
25	146.4	81.8	131.1	131.2	131.2	144.9	131.2
26	113.6	25.8	26.1	26.1	26.1	125.2	26.1
27	18.1	25.5	18.1	18.2	18.2	18.2	18.1
28	29.0	29.0	29.6	28.5	28.5	28.5	28.6
29	16.6	16.7	17.7	17.0	17.0	17.0	17.2
30	17.8	17.7	17.8	17.8	17.5	17.8	17.8
20-*O*-Glc							
1’	97.0	97.1	97.1	97.2	97.1	97.0	97.2
2’	80.4	81.7	81.9	81.2	84.9	79.8	81.1
3’	78.5	79.0	79.2	79.3	78.2	78.7	78.6
4’	71.7	71.8	72.1	72.0	71.3	71.9	71.8
5’	79.5	78.6	78.7	78.7	78.2	79.7	78.7
6’	63.0	63.2	62.9	63.1	62.6	62.9	62.9
2’-*O*-Glc							
1’’	105.1	105.9	105.8	105.5	107.1	104.8	105.4
2’’	76.8	77.0	77.0	76.9	77.2	77.4	76.1
3’’	78.4	78.8	78.5	78.7	78.4	78.7	79.4
4’’	71.9	72.0	71.8	71.8	72.0	72.0	72.1
5’’	78.7	78.6	78.7	78.7	78.6	78.6	78.5
6’’	62.9	63.0	63.2	63.0	63.1	63.0	63.3
3-*O*-Glc							
1’’’	-	-	-	105.2	105.4	105.1	107.1
2’’’	-	-	-	83.6	83.7	83.6	76.9
3’’’	-	-	-	78.6	78.7	78.6	79.1
4’’’	-	-	-	71.8	71.9	71.7	71.8
5’’’	-	-	-	78.3	78.3	78.6	78.7
6’’’	-	-	-	63.1	63.2	63.1	63.0
2’’’-*O*-Glc							
1’’’’	-	-	-	106.3	106.3	106.3	-
2’’’’	-	-	-	77.4	77.5	76.7	-
3’’’’	-	-	-	78.6	78.7	78.3	-
4’’’’	-	-	-	71.8	71.4	71.7	-
5’’’’	-	-	-	78.3	75.9	78.3	-
6’’’’	-	-	-	62.8	64.8	63.1	-
6’’’’-OCOCH_3_	-	-	-	-	171.3	-	-
CH_3_COO	-	-	-	-	21.2	-	-

**Table 3:** ^1^H-NMR and ^13^C-NMR spectral data of compound **7** (600 MHz and 150 MHz, pyridine-*d*_5_, δ in ppm, *J* in Hz).

Position	^13^C	^1^H	Position	^13^C	^1^H
1	39.7	1.51 (m), 0.80 (m)	3-*O*-Glc		
2	27.2	2.24 (m), 1.87 (m)	1’	105.4	4.96 (d, 7.6)
3	89.4	3.33 (dd,11.7, 4.6)	2’	77.4	4.15 (m)
4	40.1	-	3’	78.6	4.02 (m)
5	56.8	0.74 (m)	4’	72.0	4.36 (m)
6	18.8	1.52 (m), 1.39(m)	5’	78.3	4.28 (m)
7	36.0	1.53 (m), 1.25 (m)	6’	63.2	4.56 (m)
8	41.0	-	20-*O*-Glc		
9	51.3	1.33 (m)	1’’	97.5	5.13 (d, 7.6)
10	37.3	-	2’’	83.7	4.23 (m)
11	22.3	1.33 (m), 1.24 (m)	3’’	83.7	4.34 (m)
12	25.6	2.23 (m), 2.04 (m)	4’’	72.0	4.36 (m)
13	43.2	1.84 (m)	5’’	78.4	3.95 (m)
14	51.1	-	6’’	63.1	4.51 (m)
15	31.8	1.67 (m),1.15 (m)	2’’-*O*-Glc		
16	28.2	2.07 (m),1.44 (m)	1’’’	106.3	5.38 (d, 7.6)
17	48.2	2.34 (m)	2’’’	77.7	4.16 (m)
18	16.2	1.01 (s)	3’’’	78.2	4.29 (m)
19	16.8	0.83 (s)	4’’’	71.9	4.19 (m)
20	83.5	-	5’’’	78.7	4.29 (m)
21	21.8	1.53 (s)	6’’’	63.1	4.34 (m)
22	39.8	2.01 (m), 1.86 (m)	3’’-*O*-Glc		
23	23.6	2.60 (m), 2.35 (m)	1’’’’	106.4	5.36 (d, 7.4)
24	126.7	5.36 (s)	2’’’’	77.9	3.84 (m)
25	130.9	-	3’’’’	79.0	4.03 (m)
26	26.2	1.71 (s)	4’’’’	72.0	4.19 (m)
27	18.4	1.71 (s)	5’’’’	78.7	4.35 (m)
28	28.4	1.31 (s)	6’’’’	63.1	4.34 (m)
29	17.0	1.13 (s)	-	-	-
30	17.0	1.04 (s)	-	-	-

**Table 4:** ^1^H NMR and ^13^C-NMR spectral data of compound **8** (600 MHz and 150 MHz, pyridine-*d*_5_, δ in ppm, *J* in Hz).

Position	^13^C	^1^H	Position	^13^C	^1^H
1	39.7	1.57 (m), 0.89 (m)	3-*O*-Glc		
2	27.4	1.98 (m), 1.41 (m)	1’	105.7	4.96 (d, 7.7)
3	89.1	3.33 (dd, 11.7, 4.4)	2’	77.0	4.13 (m)
4	40.0	-	3’	78.6	3.84 (m)
5	56.9	0.68 (m)	4’	72.5	3.95 (m)
6	18.9	1.48 (m), 1.39 (m)	5’	78.6	3.94 (m)
7	35.4	1.43 (m), 1.20 (m)	6’	63.1	4.50 (m)
8	40.3	-	20-*O*-Glc		
9	50.4	1.41 (m)	1’’	97.2	5.17 (d, 7.7)
10	37.3	-	2’’	81.7	4.24 (m)
11	31.4	1.58 (m), 1.02 (m)	3’’	79.2	4.36 (m)
12	71.2	4.10 (m)	4’’	71.8	4.29 (m)
13	49.5	1.99 (m)	5’’	78.8	4.28 (m)
14	52.1	-	6’’	62.8	4.45 (m)
15	31.0	2.13 (m), 1.42 (m)	2’’-*O*-Glc		
16	27.2	2.16 (m), 1.78 (m)	1’’’	105.8	5.64 (d, 7.7)
17	53.1	2.58 (m)	2’’’	78.3	4.28 (m)
18	17.3	1.18 (s)	3’’’	78.5	4.16 (m)
19	16.7	0.79 (s)	4’’’	71.9	4.17 (m)
20	84.4	-	5’’’	80.2	4.29 (m)
21	22.5	1.61 (s)	6’’’	63.2	4.38 (m)
22	36.6	2.36 (m), 1.87 (m)	2’’’-*O*-Rha		
23	23.9	2.45 (m), 2.27 (m)	1’’’’	102.1	6.56 (s)
24	126.4	5.23 (t)	2’’’’	72.9	4.70 (m)
25	131.2	-	3’’’’	72.8	4.88 (m)
26	26.1	1.61 (s)	4’’’’	74.5	4.35 (m)
27	18.2	1.64 (s)	5’’’’	70.0	4.79 (m)
28	16.2	0.93 (s)	6’’’’	19.1	1.71 (d, 6.2)
29	28.4	1.28 (s)			
30	17.8	1.00 (s)			

**Table 5:** ^1^H-NMR and ^13^C-NMR spectral data of compound **10** (600 MHz and 150 MHz, pyridine-*d*_5_, δ in ppm, *J* in Hz).

Position	^13^C	^1^H	Position	^13^C	^1^H
1	47.8	2.45 (m), 1.14 (m)	3-*O*-Glc		
2	67.2	4.01 (m)	1’	104.8	4.95 (d, 7.8)
3	95.7	3.21 (m)	2’	77.1	4.49 (m)
4	41.3	-	3’	78.5	4.14 (m)
5	56.4	0.80 (m)	4’	71.7	4.29 (m)
6	18.8	1.51 (m), 1.38 (m)	5’	78.5	4.14 (m)
7	35.3	1.46 (m), 1.22 (m)	6’	63.1	4.44 (m)
8	40.3	-	20-*O*-Glc		
9	50.4	1.54 (m)	1’’	97.2	5.18 (d, 7.8)
10	38.2	-	2’’	81.9	4.23 (m)
11	31.2	1.56 (m), 1.02 (m)	3’’	79.2	4.15 (m)
12	71.0	4.15 (m)	4’’	71.5	4.15 (m)
13	49.5	2.00 (m)	5’’	78.6	4.14 (m)
14	51.9	-	6’’	62.8	4.29 (m)
15	31.0	2.31 (m), 1.56 (m)	2’’-*O*-Glc		
16	27.2	1.98 (m), 1.42 (m)	1’’’	105.8	5.65 (d, 7.8)
17	53.1	2.59 (m)	2’’’	82.6	4.30 (1m)
18	16.2	0.95 (s)	3’’’	78.9	3.97 (m)
19	17.7	0.88 (s)	4’’’	71.9	4.29 (m)
20	84.4	-	5’’’	78.5	4.14 (m)
21	22.3	1.62 (s)	6’’’	62.7	4.29 (m)
22	36.5	2.38 (m), 1.89 (m)	2’’’-*O*-Glc		
23	23.8	2.47 (m), 2.27 (m)	1’’’’	105.9	5.52 (d, 7.8)
24	126.3	5.24 (t, 7.0)	2’’’’	77.1	4.49 (m)
25	131.2	-	3’’’’	78.7	3.85 (m)
26	26.1	1.62 (s)	4’’’’	71.3	4.52 (m)
27	18.2	1.65 (s)	5’’’’	78.7	4.30 (m)
28	28.6	1.34 (s)	6’’’’	63.3	4.52 (m)
29	18.0	1.20 (s)			
30	17.7	0.99 (s)			

## Supplementary Materials

The following are available online, The HR-ESI-MS, ^1^H-NMR, ^13^C-NMR, DEPT-135, HMBC, HSQC, and ^1^H-^1^H COSY spectra for compounds **1**–**10** can be seen in Figures S1–S70. The identification of compound **11** can be seen in Figures S71–S74.
