# Supplementary material for: Ten New Dammarane-Type Saponins with Hypolipidemia Activity from a Functional Herbal Tea—Gynostemma pentaphyllum
Source: Molecules. 2020 Aug 15;25(16):3737. doi: 10.3390/molecules25163737 (PMC7464191; doi:10.3390/molecules25163737)
Supplement: Supplementary file 1 [file molecules-25-03737-s001.zip › Supporting Information2020.08.06/Supporting Information.pdf]

Article

Ten new dammarane-type saponins with hypolipidemia activity from a functional herbal tea-*Gynostemma pentaphyllum*

Maojing Yin [1,2,†], Jingjing Zhang [1,2,†], Lizhi Wang [3], Fangyi Li [1,2], Zhenfa Li [1,2], Wei Xiang [1,2], Songtao Bie [1,2], Chunhua Wang [1,2,*], Zheng Li [1,2,*]

[1] College of Pharmaceutical Engineering of Traditional Chinese Medicine, Tianjin University of Traditional Chinese Medicine, Tianjin 301617, China; yinmaojing11@163.com (M.Y.); zjj20190924@163.com (J.Z.); li_fangyi@hotmail.com (F.L.); li-zhenfa@hotmail.com (Z.L.); 15922162979@163.com (W.X.); biesongtao@126.com (S.B.)

[2] Tianjin Key Laboratory of Modern Chinese Medicine, Tianjin University of Traditional Chinese Medicine, Tianjin 301617, China;

[3] School of Chinese Materia Medicine, Tianjin University of Traditional Chinese Medicine, Tianjin 301617, China; lzhwang_2009@126.com (L.W.)

* Correspondence: pharmwch@126.com (C.W.) or lizheng@tjutcm.edu.cn (Z.L.); Tel.: +86-22-5976-1813 (C.W.)

† These authors contributed equally to this work.

Table of contents

Figure S1. High-resolution electrospray ionization mass spectrum of compound **1**

Figure S2. ^{1}H NMR spectrum of compound **1**

Figure S3. ^{13}C NMR spectrum of compound **1**

Figure S4. DEPT-135 spectrum of compound **1**

Figure S5. HMBC spectrum of compound **1**

Figure S6. HSQC spectrum of compound **1**

Figure S7. ^{1}H-^{1}H COSY spectrum of compound **1**

Figure S8. High-resolution electrospray ionization mass spectrum of compound **2**

Figure S9. ^{1}H NMR spectrum of compound **2**

Figure S10. ^{13}C NMR spectrum of compound **2**

Figure S11. DEPT-135 spectrum of compound **2**

Figure S12. HMBC spectrum of compound **2**

Figure S13. HSQC spectrum of compound **2**

Figure S14. ^{1}H-^{1}H COSY spectrum of compound **2**

Figure S15. High-resolution electrospray ionization mass spectrum of compound **3**

Figure S16. ^{1}H NMR spectrum of compound **3**

Figure S17. ^{13}C NMR spectrum of compound **3**

Figure S18. DEPT-135 spectrum of compound **3**

Figure S19. HMBC spectrum of compound **3**

Figure S20. HSQC spectrum of compound **3**

Figure S21. ^{1}H-^{1}H COSY spectrum of compound **3**

Figure S22. High-resolution electrospray ionization mass spectrum of compound **4**

Figure S23. ^{1}H NMR spectrum of compound **4**

Figure S24. ^{13}C NMR spectrum of compound **4**

Figure S25. DEPT-135 spectrum of compound **4**

Figure S26. HMBC spectrum of compound **4**

Figure S27. HSQC spectrum of compound **4**

Figure S28. ^{1}H-^{1}H COSY spectrum of compound **4**

Figure S29. High-resolution electrospray ionization mass spectrum of compound **5**

Figure S30. ^{1}H NMR spectrum of compound **5**

Figure S31. ^{13}C NMR spectrum of compound **5**

Figure S32 DEPT-135 spectrum of compound **5**

Figure S33. HMBC spectrum of compound **5**

Figure S34. HSQC spectrum of compound **5**

Figure S35. ^{1}H-^{1}H COSY spectrum of compound **5**

Figure S36. High-resolution electrospray ionization mass spectrum of compound **6**

Figure S37. ^{1}H NMR spectrum of compound **6**

Figure S38. ^{13}C NMR spectrum of compound **6**

Figure S39. DEPT-135 spectrum of compound **6**

Figure S40. HMBC spectrum of compound **6**

Figure S41. HSQC spectrum of compound **6**

Figure S42. ^{1}H-^{1}H COSY spectrum of compound **6**

Figure S43. High-resolution electrospray ionization mass spectrum of compound **7**

Figure S44. ^{1}H NMR spectrum of compound **7**

Figure S45. ^{13}C NMR spectrum of compound **7**

Figure S46. DEPT-135 spectrum of compound **7**

Figure S47. HMBC spectrum of compound **7**

Figure S48. HSQC spectrum of compound **7**

Figure S49. ^{1}H-^{1}H COSY spectrum of compound **7**

Figure S50. High-resolution electrospray ionization mass spectrum of compound **8**

Figure S51. ^{1}H NMR spectrum of compound **8**

Figure S52. ^{13}C NMR spectrum of compound **8**

Figure S53. DEPT-135 spectrum of compound **8**

Figure S54. HMBC spectrum of compound **8**

Figure S55. HSQC spectrum of compound **8**

Figure S56. ^{1}H-^{1}H COSY spectrum of compound **8**

Figure S57. High-resolution electrospray ionization mass spectrum of compound **9**

Figure S58. ^{1}H NMR spectrum of compound **9**

Figure S59. ^{13}C NMR spectrum of compound **9**

Figure S60. DEPT-135 spectrum of compound **9**

Figure S61. HMBC spectrum of compound **9**

Figure S62. HSQC spectrum of compound **9**

Figure S63. ^{1}H-^{1}H COSY spectrum of compound **9**

Figure S64. High-resolution electrospray ionization mass spectrum of compound **10**

Figure S65. ^{1}H NMR spectrum of compound **10**

Figure S66. ^{13}C NMR spectrum of compound **10**

Figure S67. DEPT-135 spectrum of compound **10**

Figure S68. HMBC spectrum of compound **10**

Figure S69. HSQC spectrum of compound **10**

Figure S70. ^{1}H-^{1}H COSY spectrum of compound **10**

Figure S71. High-resolution electrospray ionization mass spectrum of compound **11**

Figure S72. ^{1}H NMR spectrum of compound **11**

Figure S73. ^{13}C NMR spectrum of compound **11**

Figure S74. DEPT-135 spectrum of compound **11**

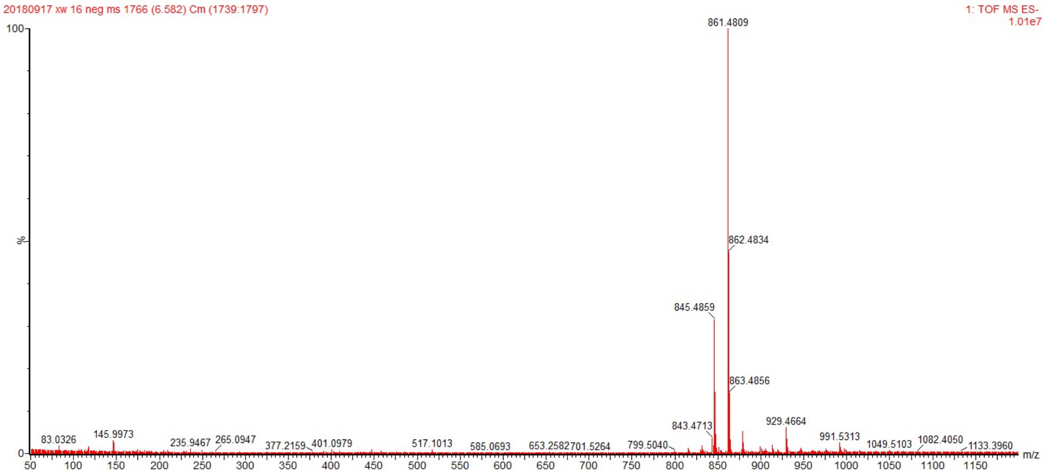

Figure S1. High-resolution electrospray ionization mass spectrum of compound **1**

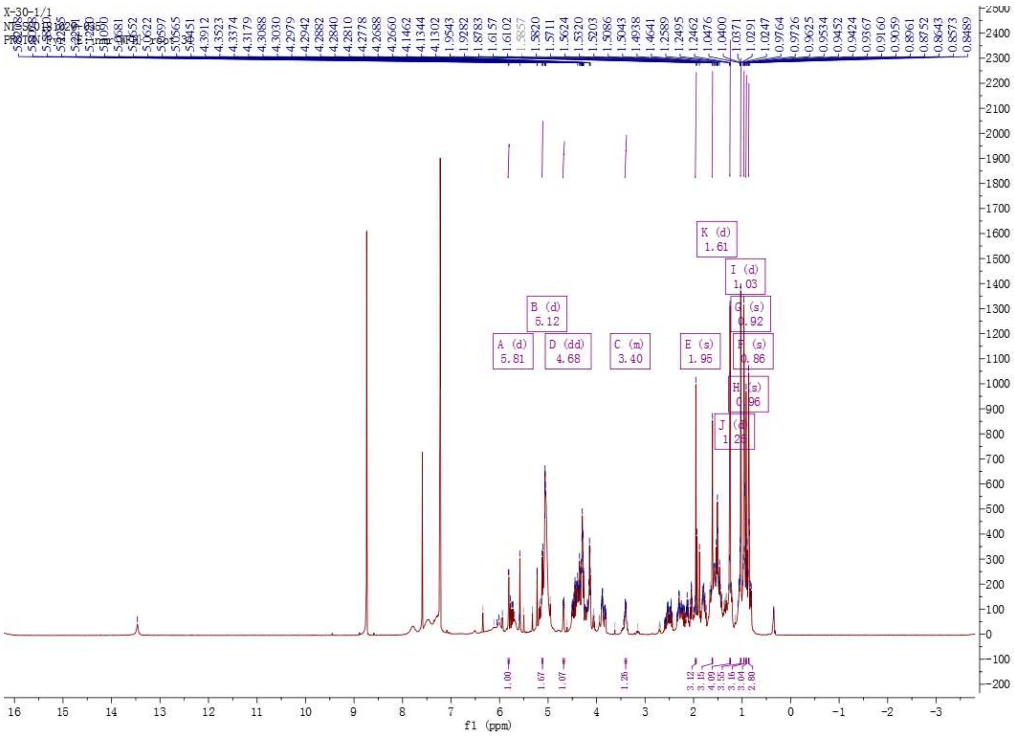

Figure S2. ^{1}H NMR spectrum of compound **1**

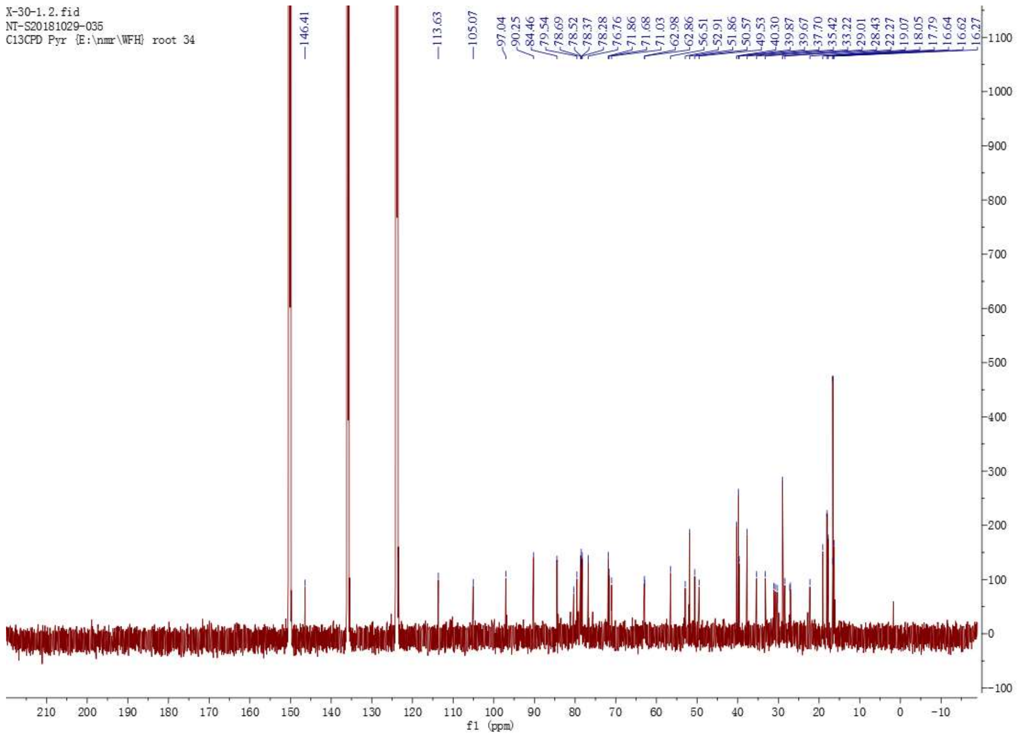

Figure S3. ^{13}C NMR spectrum of compound **1**

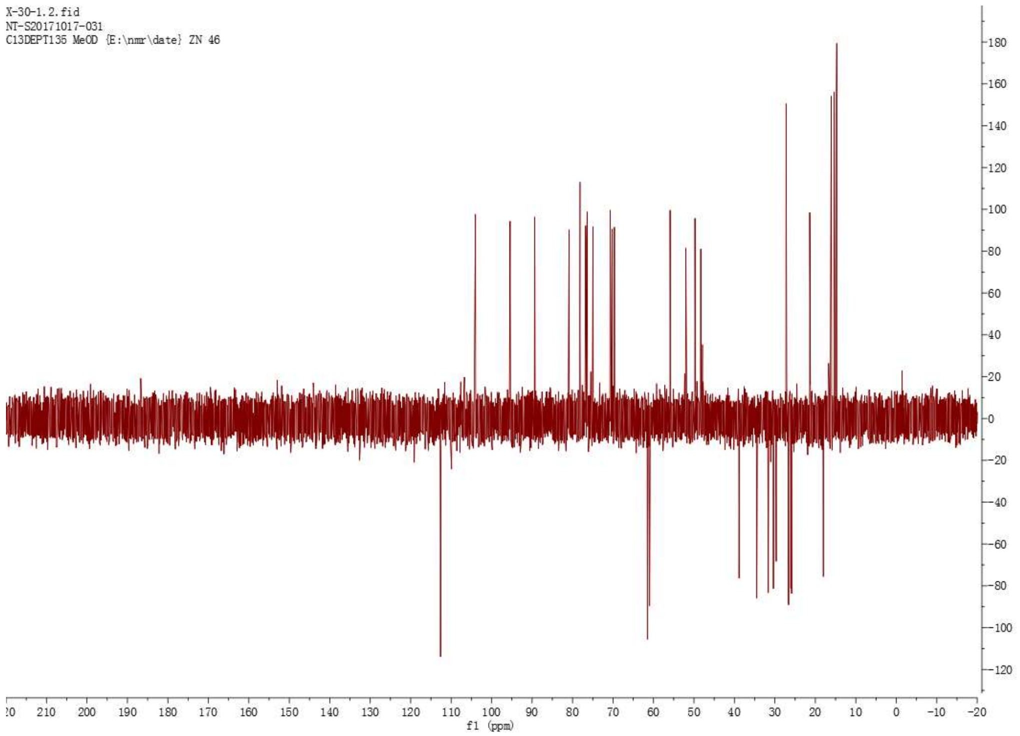

Figure S4. DEPT-135 spectrum of compound **1**

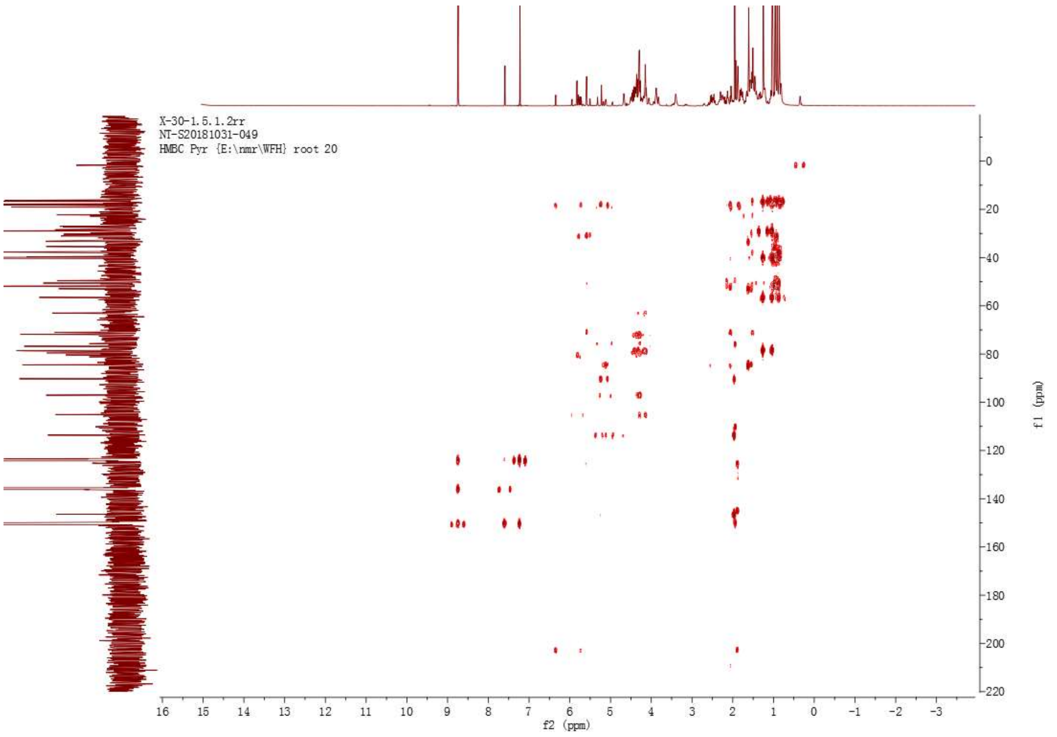

Figure S5. HMBC spectrum of compound **1**

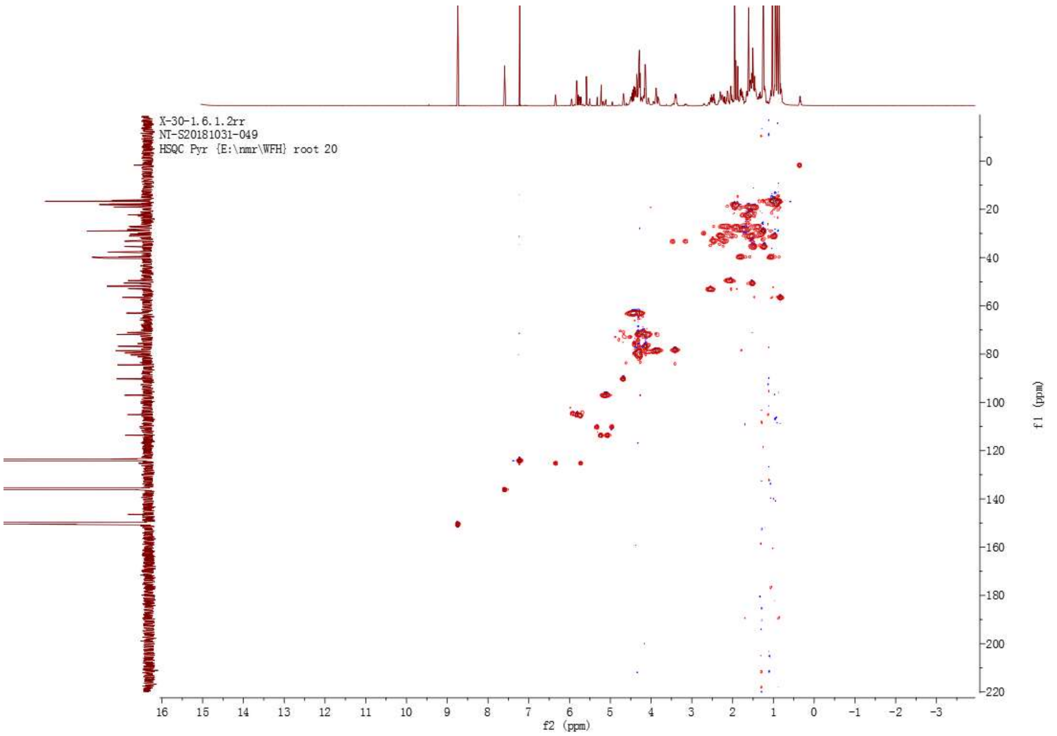

Figure S6. HSQC spectrum of compound **1**

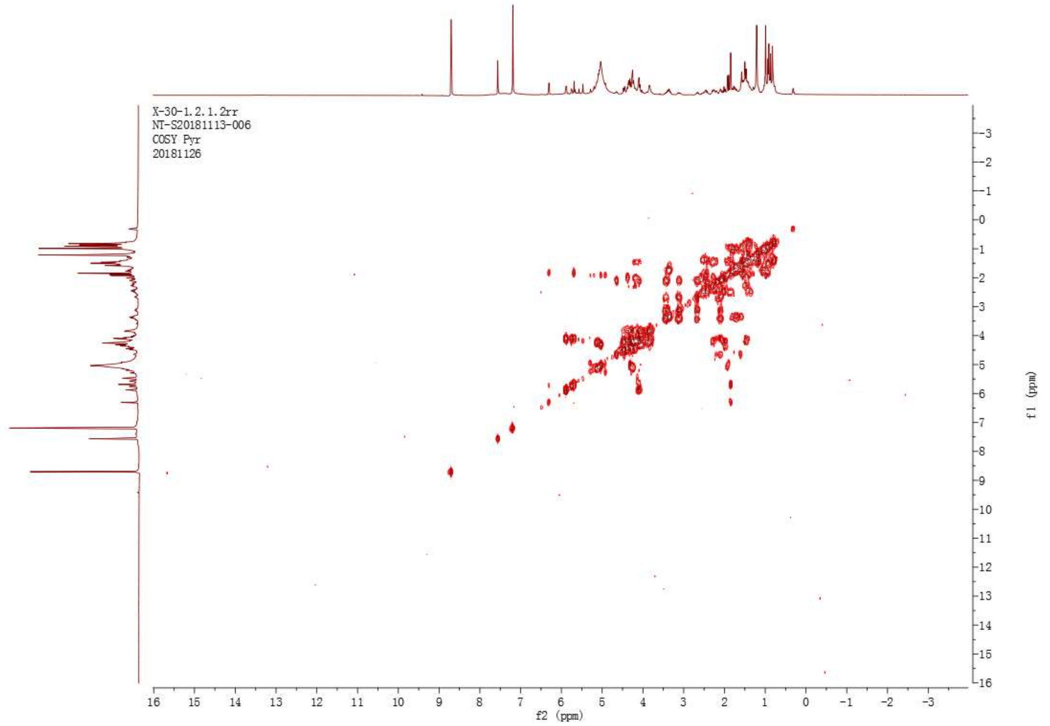

Figure S7. ^{1}H-^{1}H COSY spectrum of compound **1**

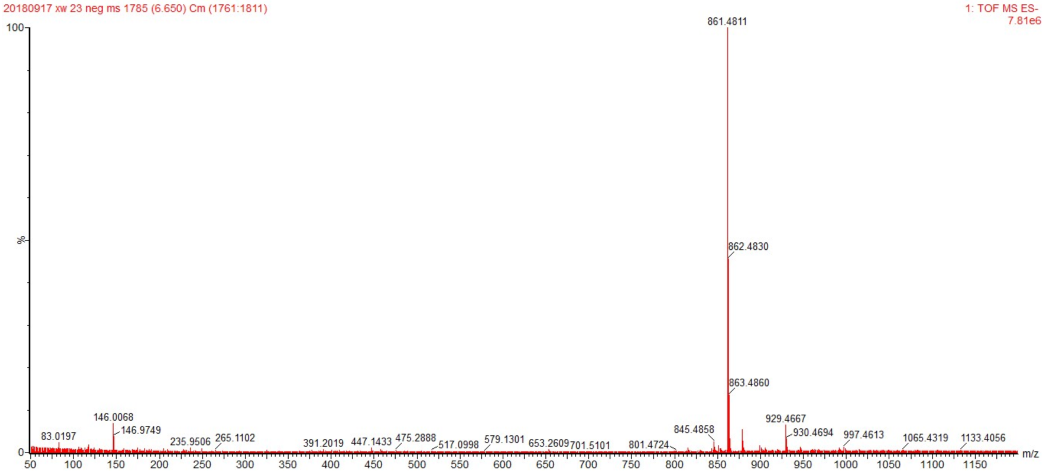

Figure S8. High-resolution electrospray ionization mass spectrum of compound **2**

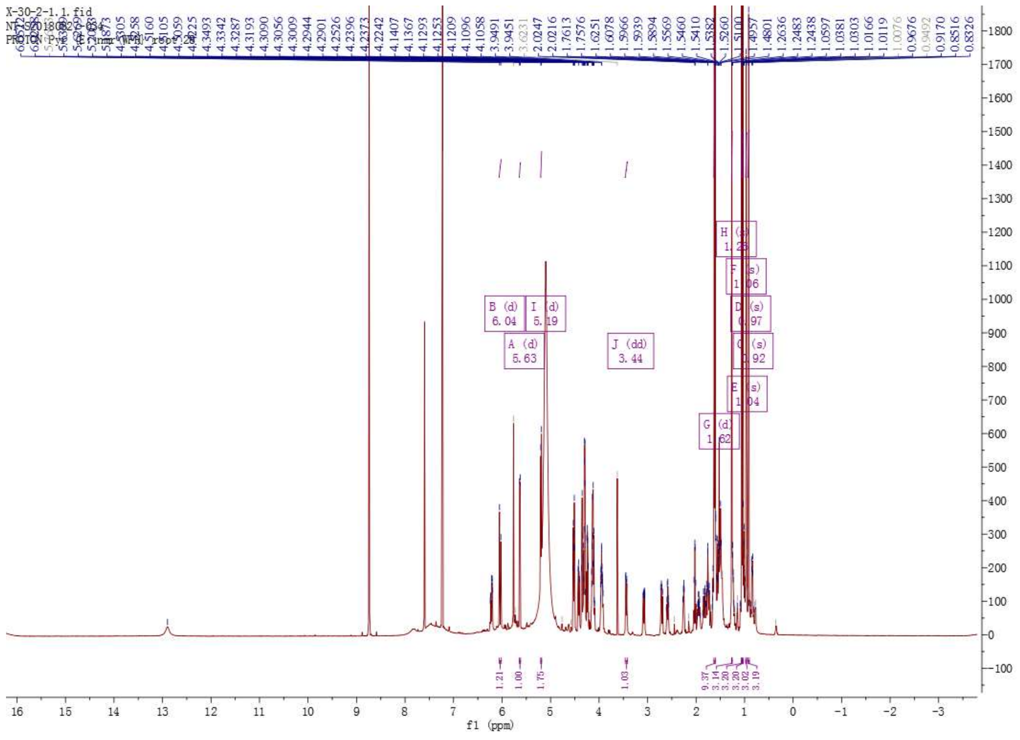

Figure S9. ^{1}H NMR spectrum of compound **2**

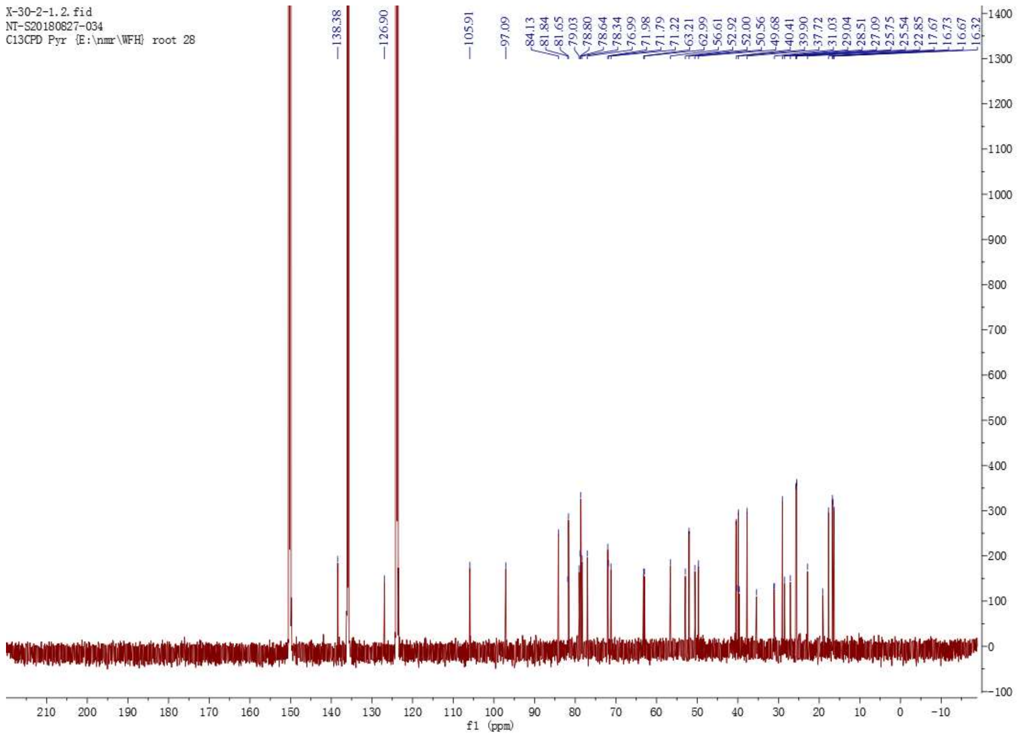

Figure S10. ^{13}C NMR spectrum of compound **2**

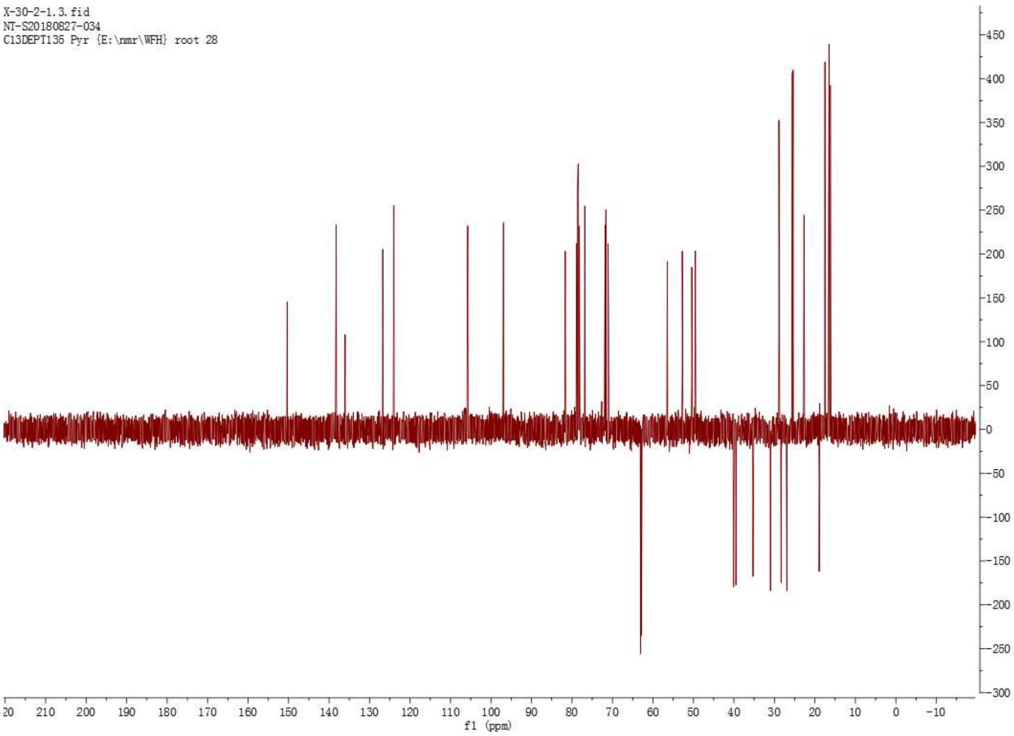

Figure S11. DEPT-135 spectrum of compound **2**

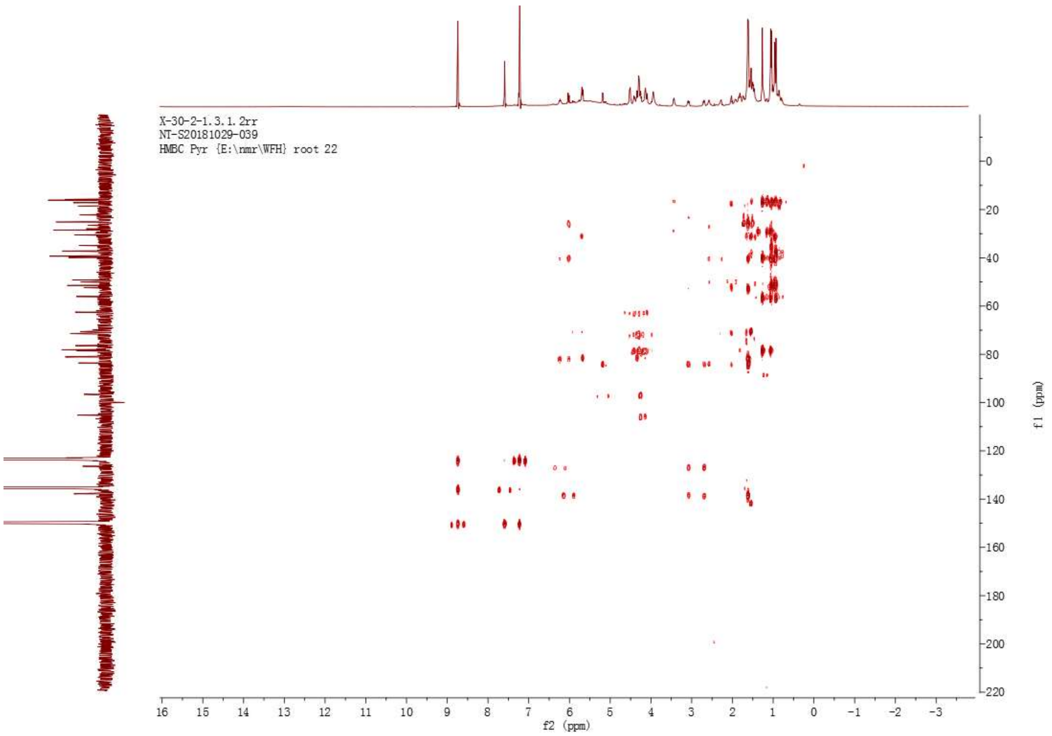

Figure S12. HMBC spectrum of compound **2**

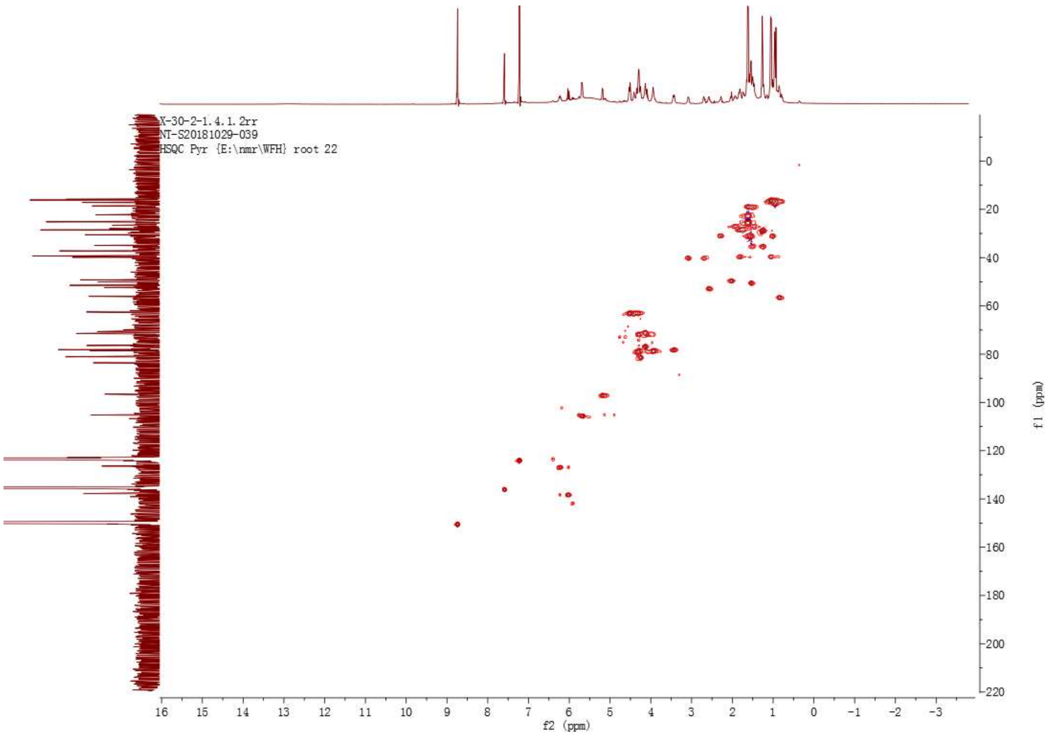

Figure S13. HSQC spectrum of compound **2**

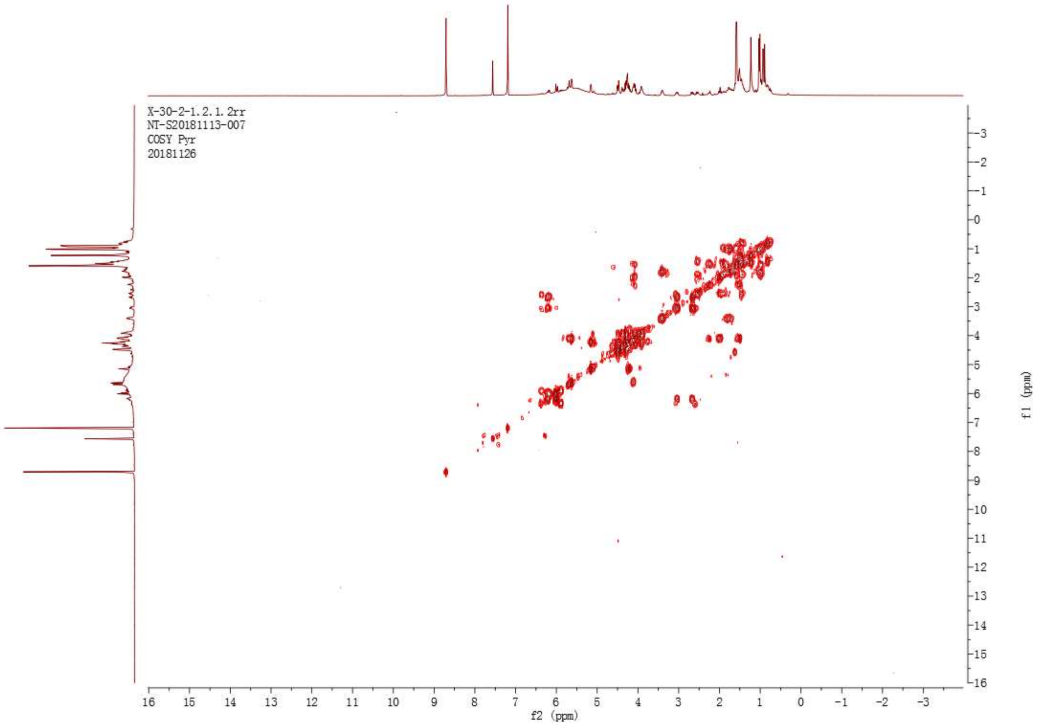

Figure S14. ^{1}H-^{1}H COSY spectrum of compound **2**

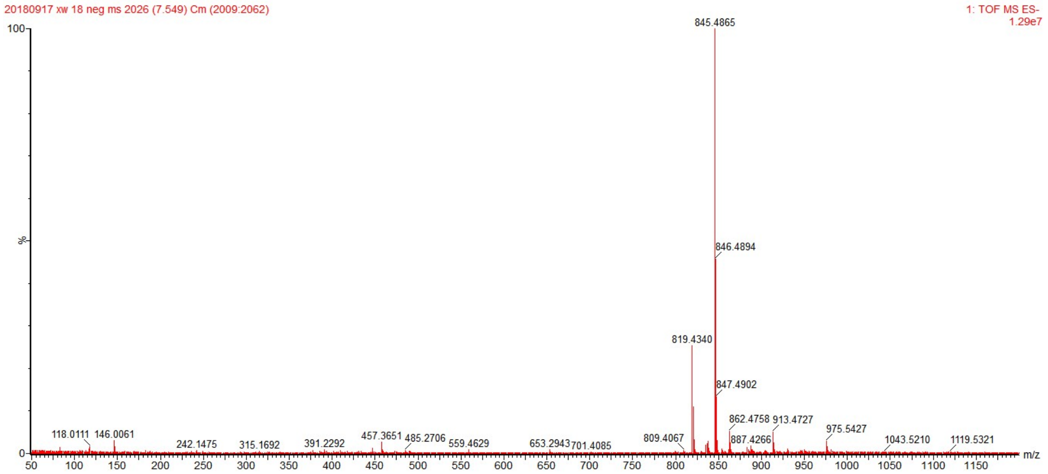

Figure S15. High-resolution electrospray ionization mass spectrum of compound **3**

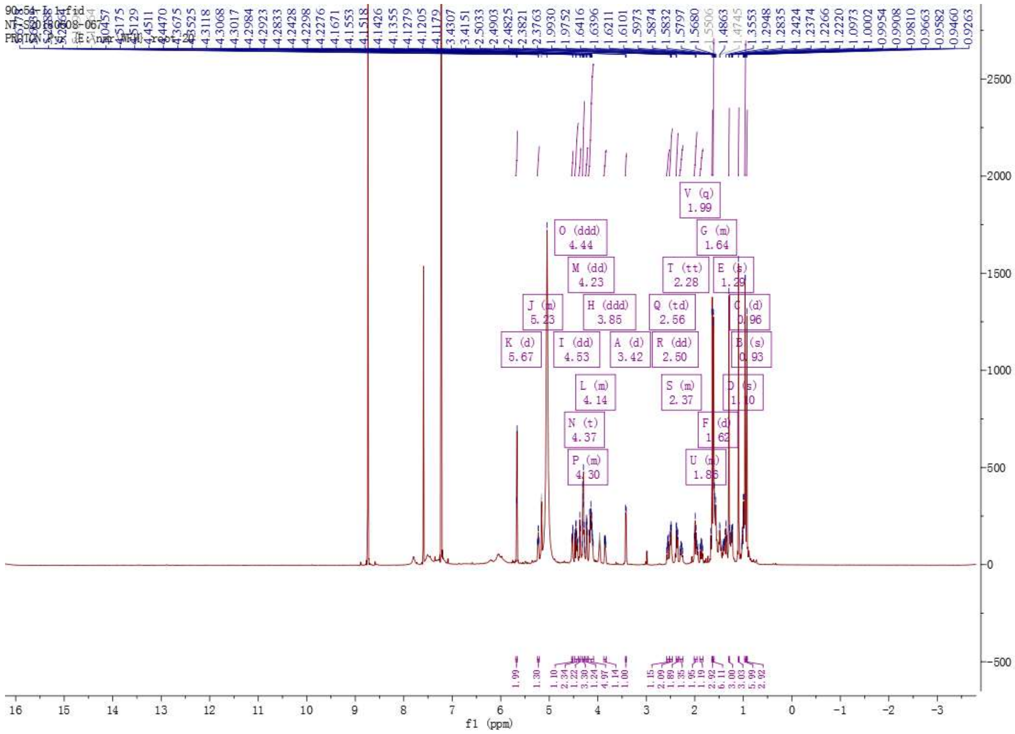

Figure S16. ^{1}H NMR spectrum of compound **3**

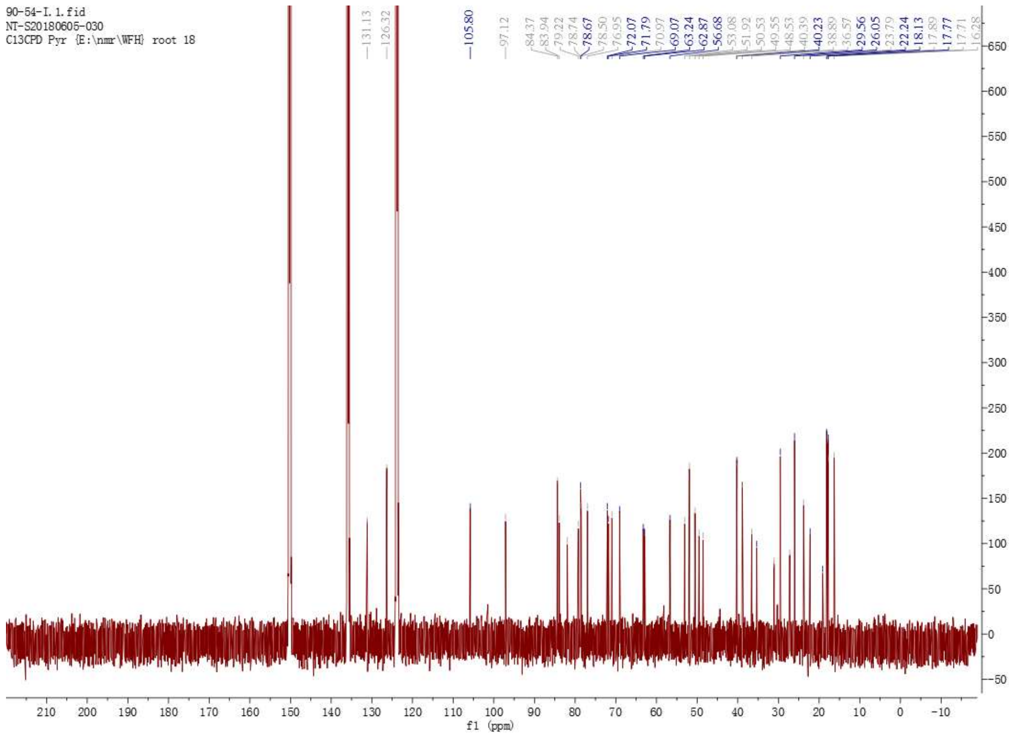

Figure S17. ^{13}C NMR spectrum of compound **3**

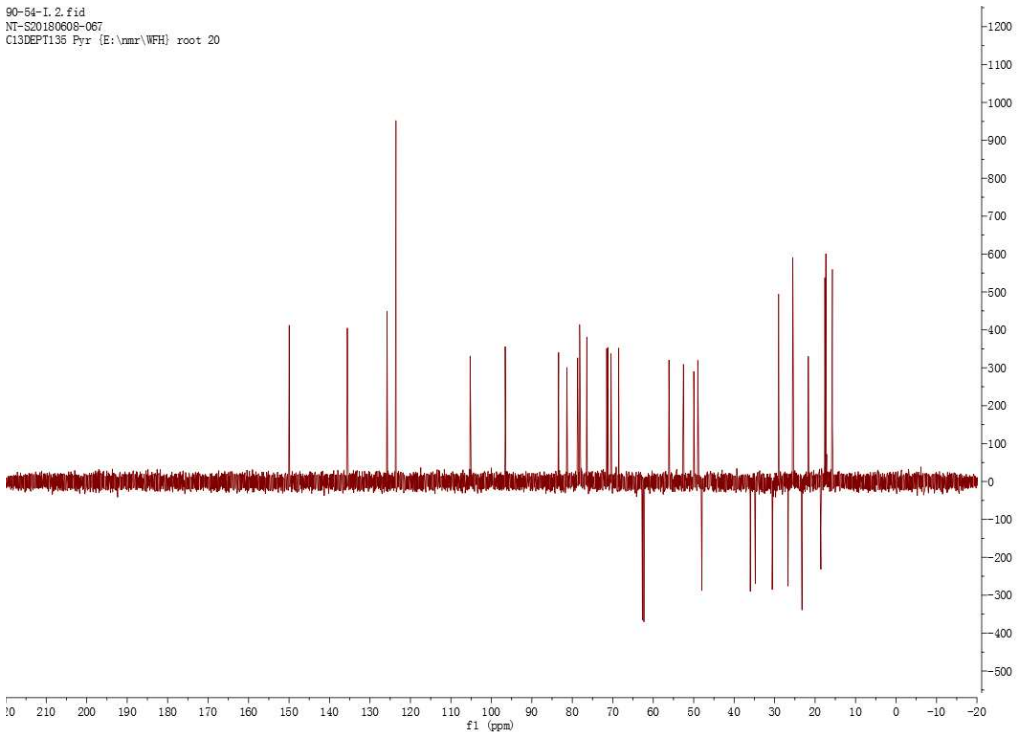

Figure S18. DEPT-135 spectrum of compound **3**

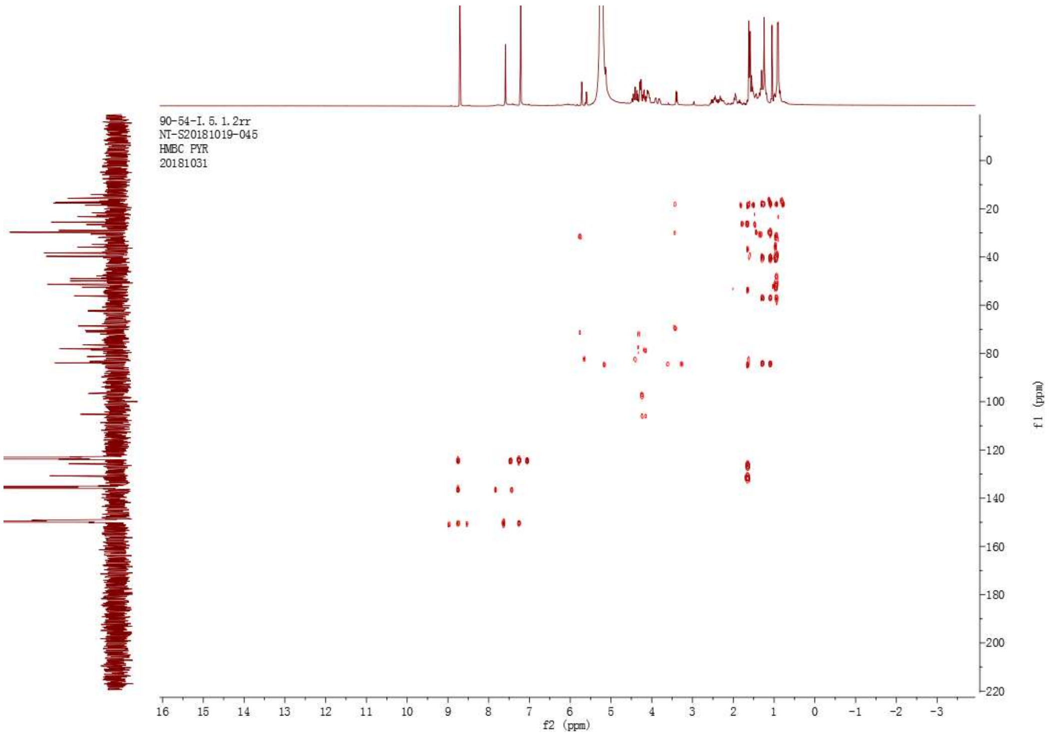

Figure S19. HMBC spectrum of compound **3**

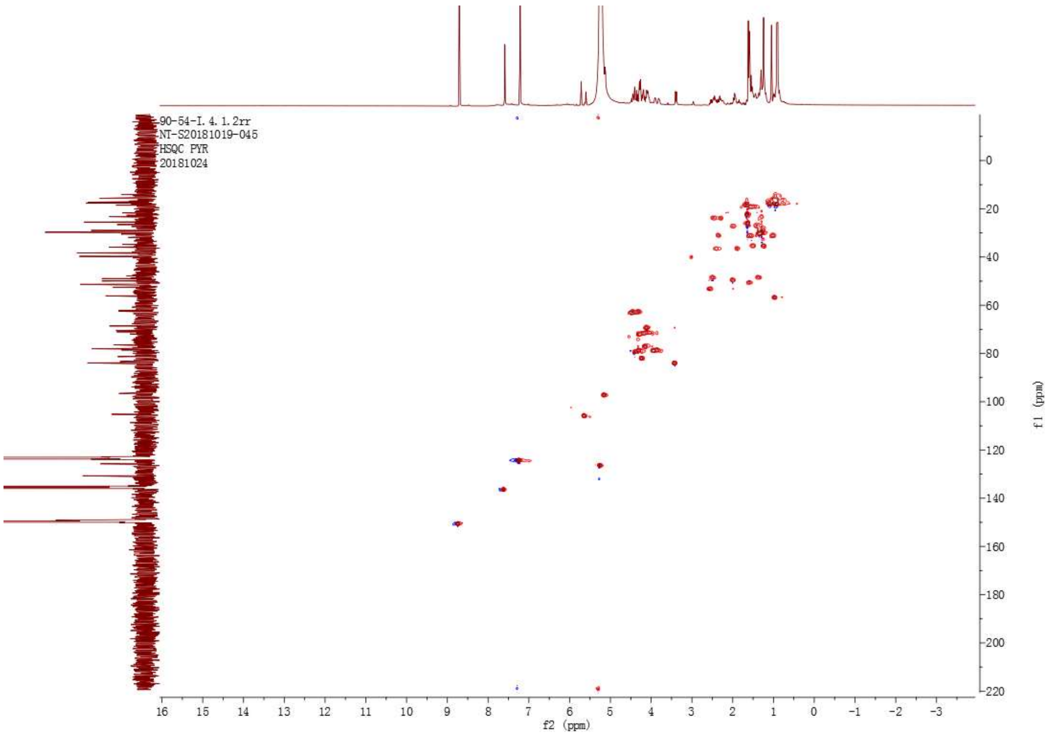

Figure S20. HSQC spectrum of compound **3**

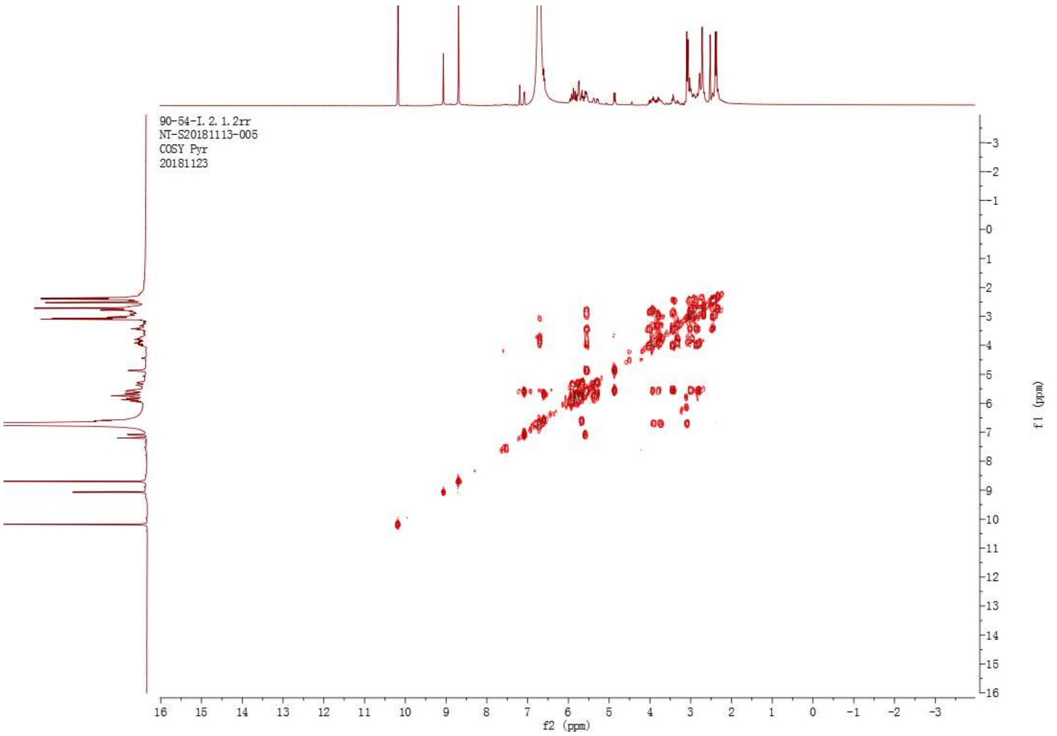

Figure S21. ^{1}H-^{1}H COSY spectrum of compound **3**

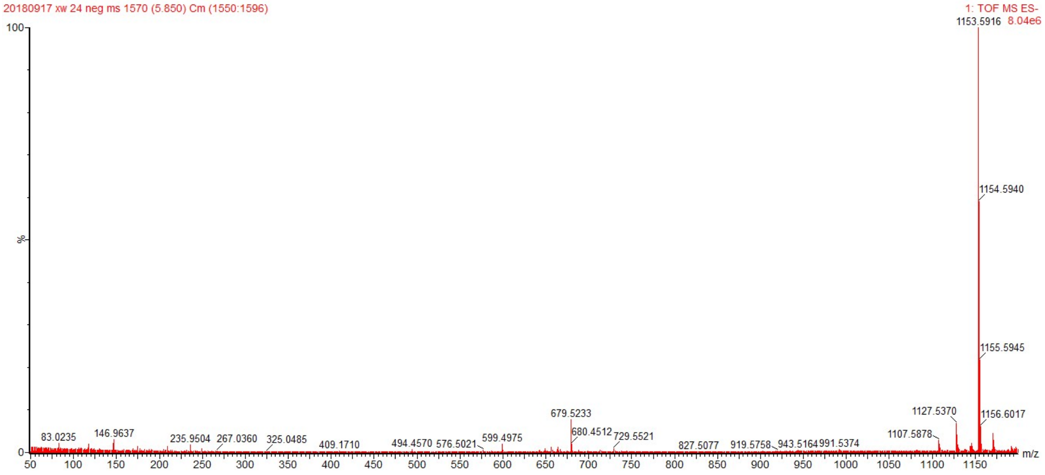

Figure S22. High-resolution electrospray ionization mass spectrum of compound **4**

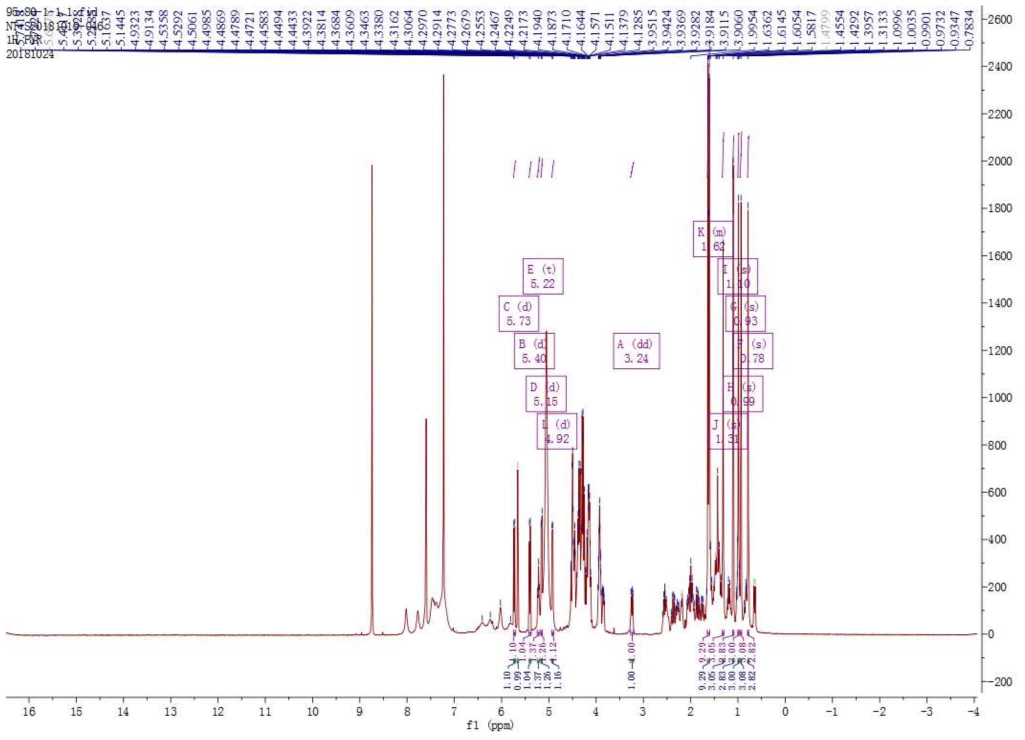

Figure S23. ^{1}H NMR spectrum of compound **4**

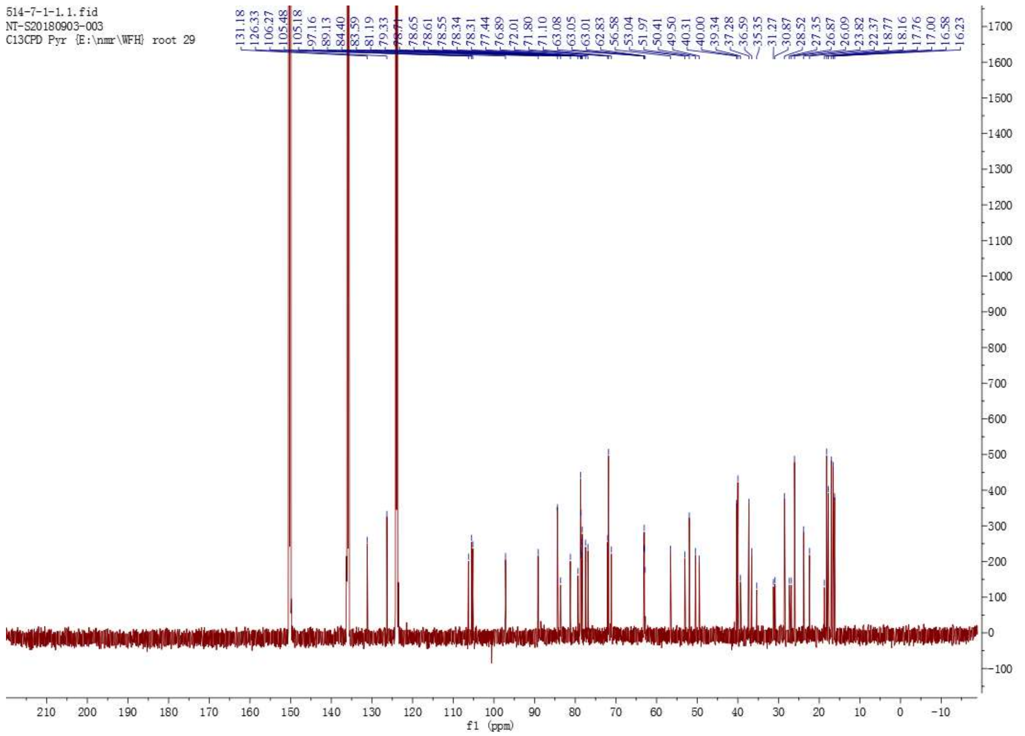

Figure S24. ^{13}C NMR spectrum of compound **4**

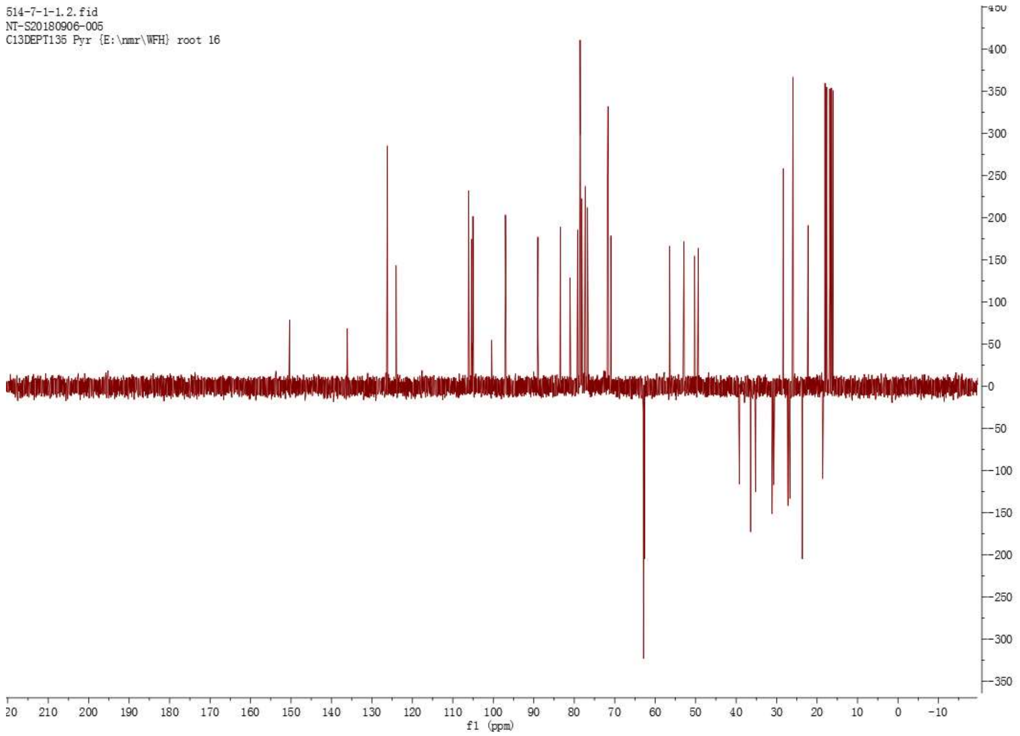

Figure S25. DEPT-135 spectrum of compound **4**

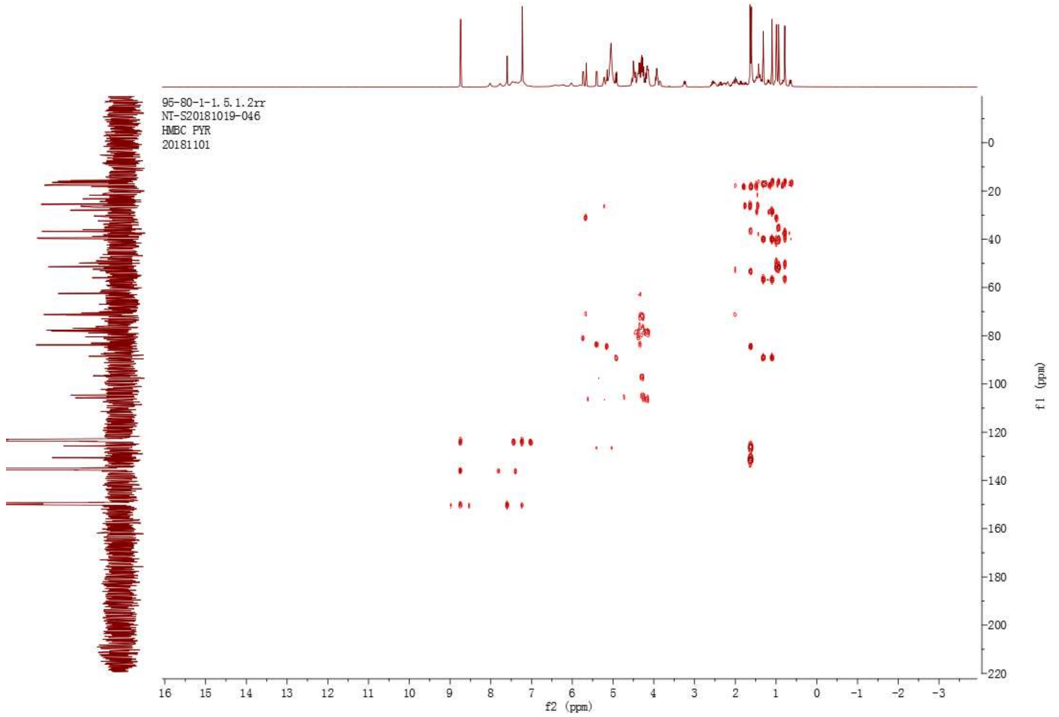

Figure S26. HMBC spectrum of compound **4**

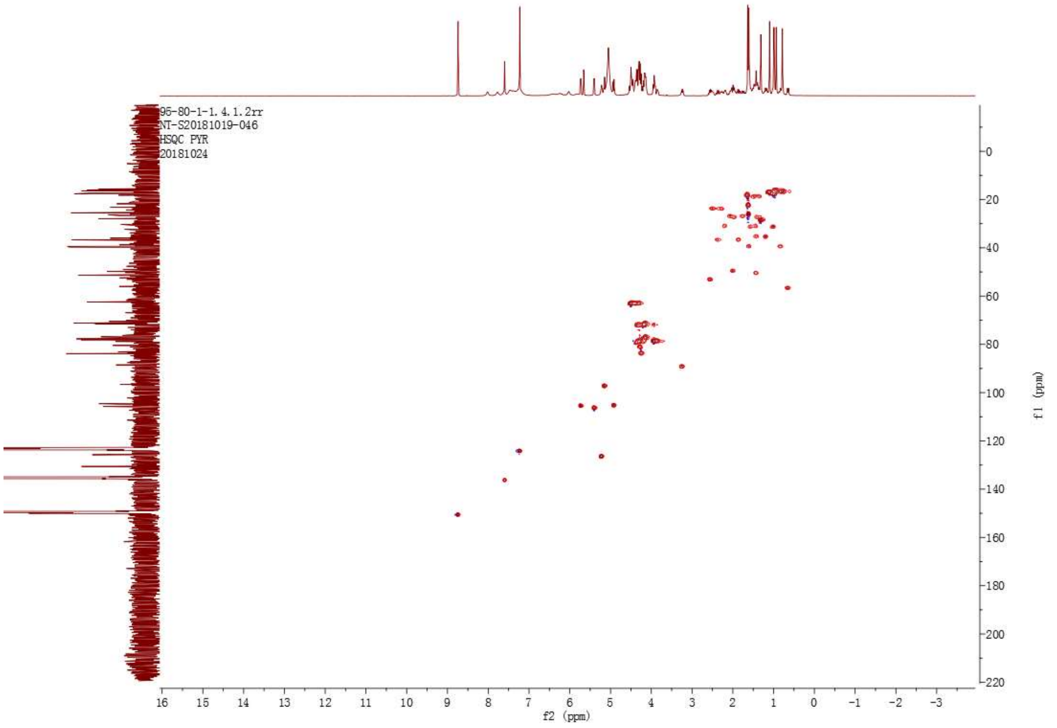

Figure S27. HSQC spectrum of compound **4**

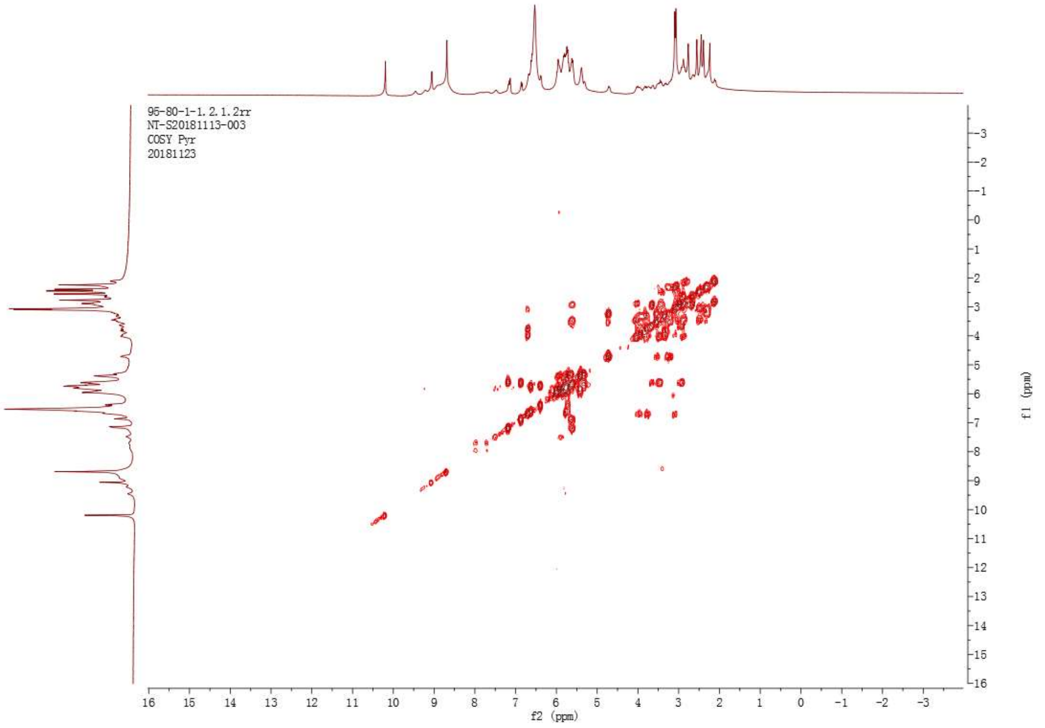

Figure S28. ^{1}H-^{1}H COSY spectrum of compound **4**

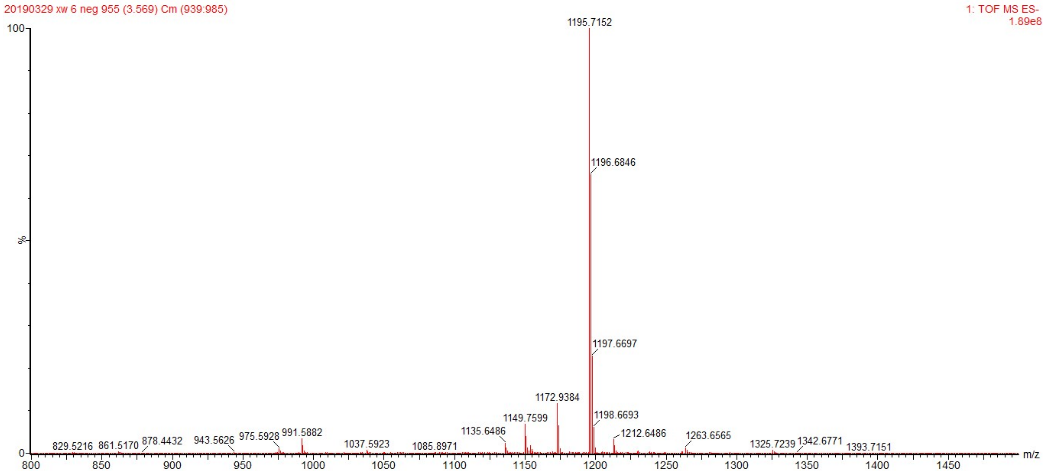

Figure S29. High-resolution electrospray ionization mass spectrum of compound **5**

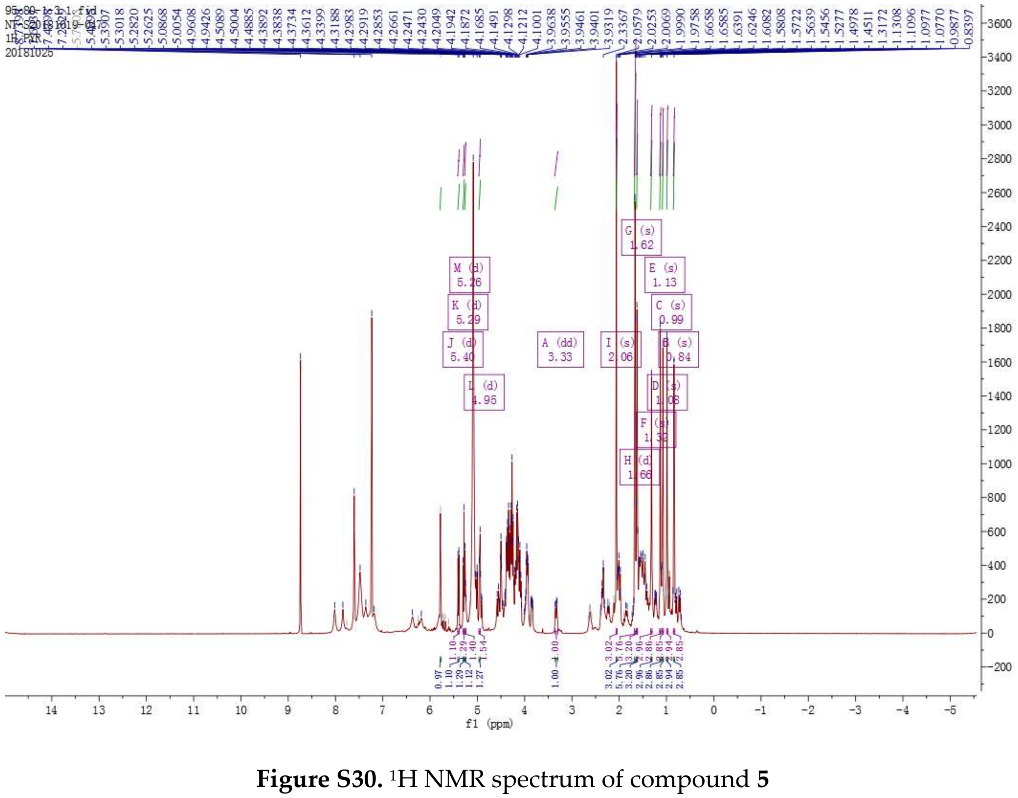

Figure S30. ^{1}H NMR spectrum of compound **5**

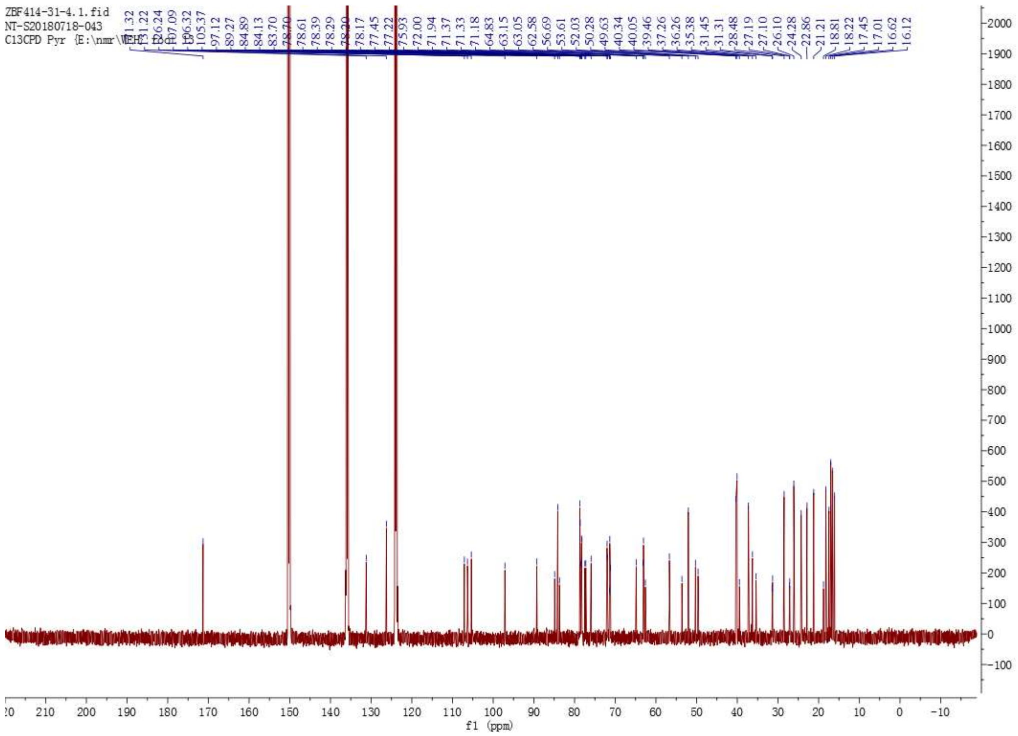

Figure S31. ^{13}C NMR spectrum of compound **5**

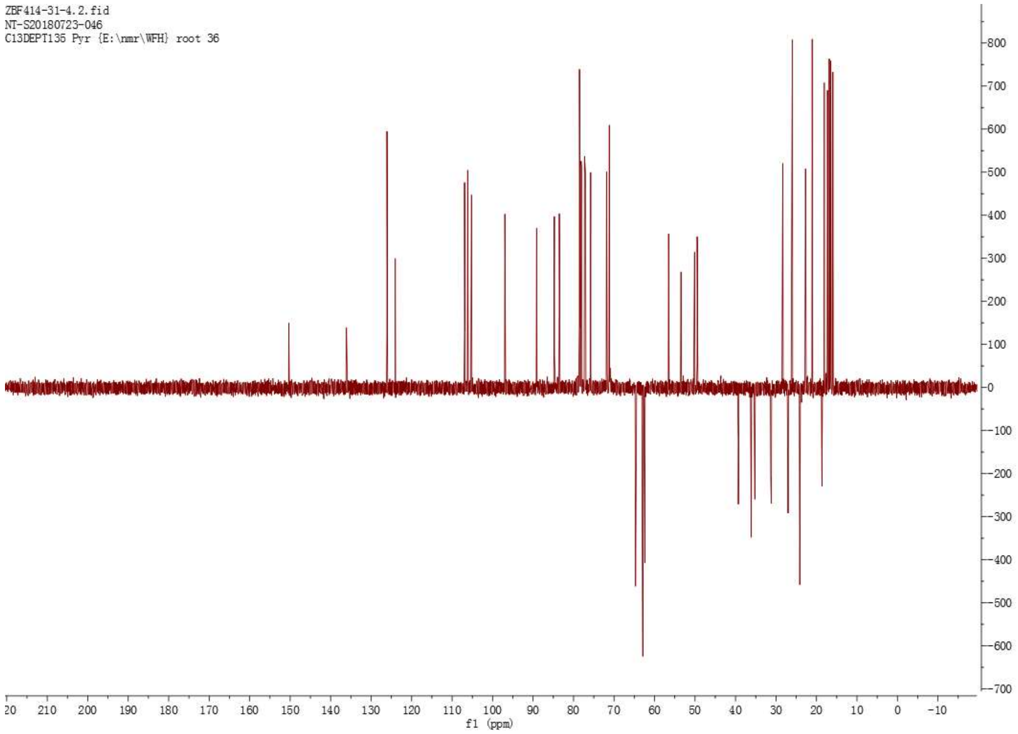

Figure S32 DEPT-135 spectrum of compound **5**

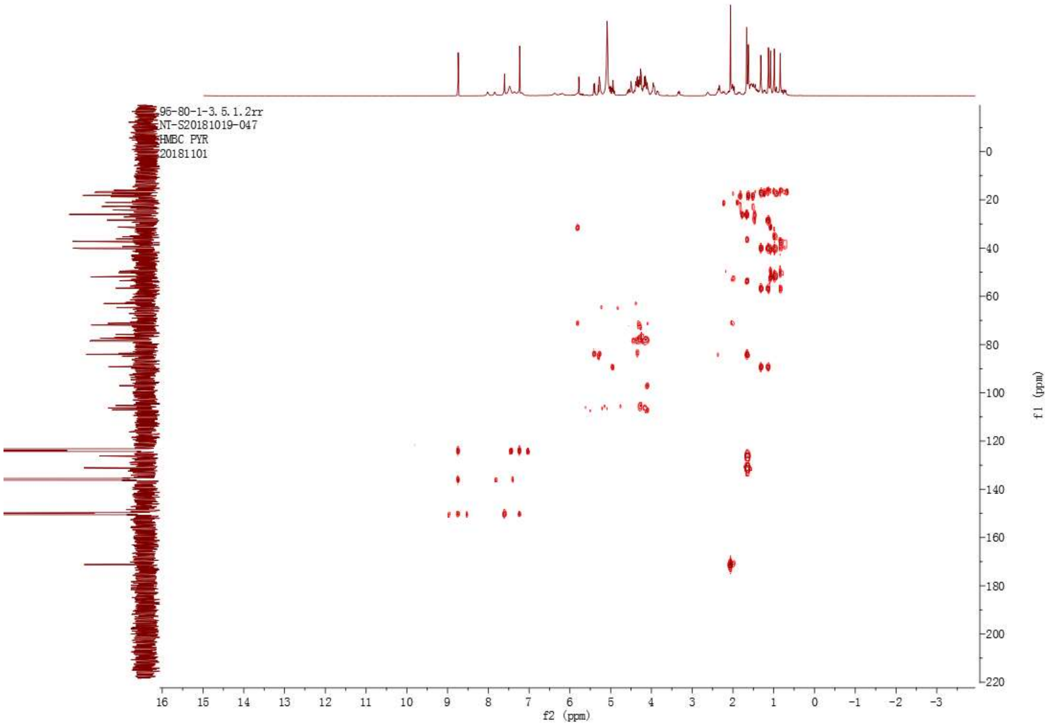

Figure S33. HMBC spectrum of compound **5**

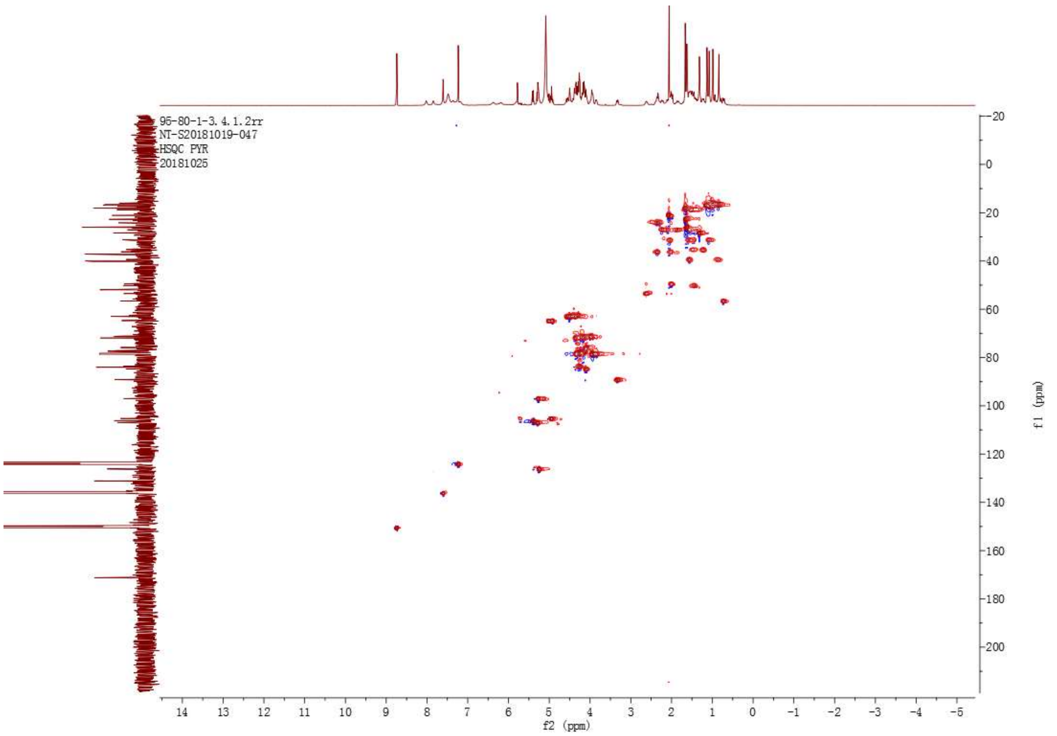

Figure S34. HSQC spectrum of compound **5**

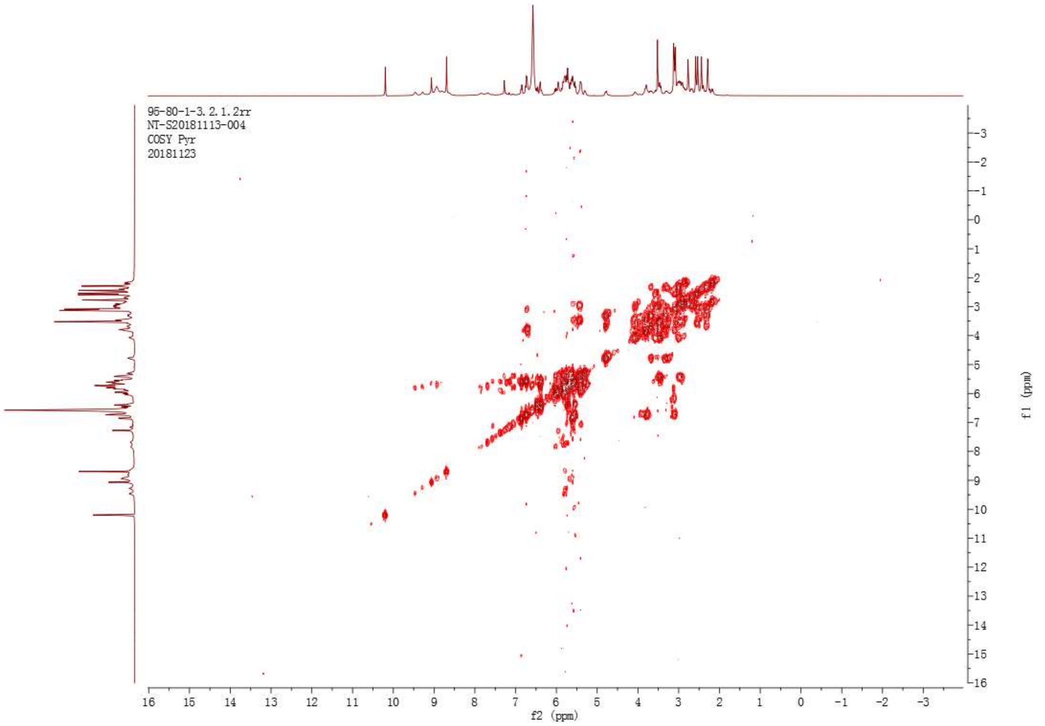

Figure S35. ^{1}H-^{1}H COSY spectrum of compound **5**

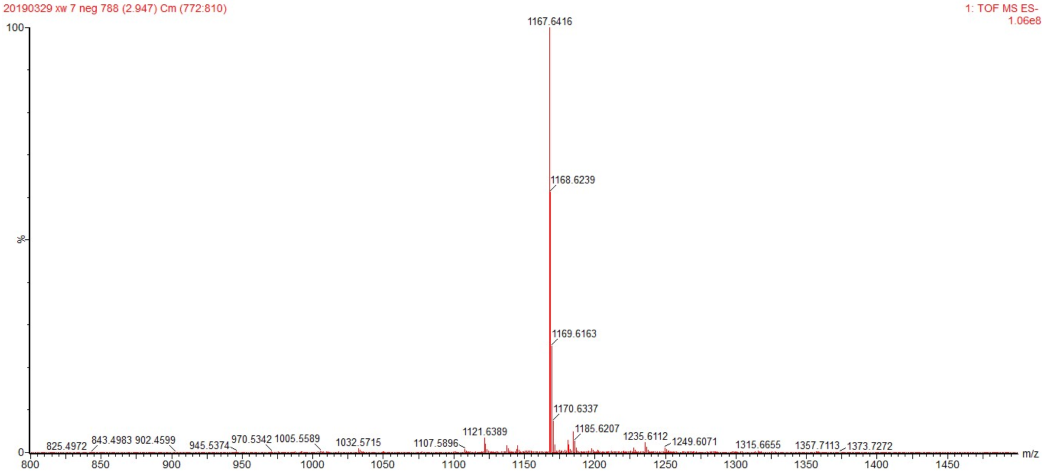

Figure S36. High-resolution electrospray ionization mass spectrum of compound **6**

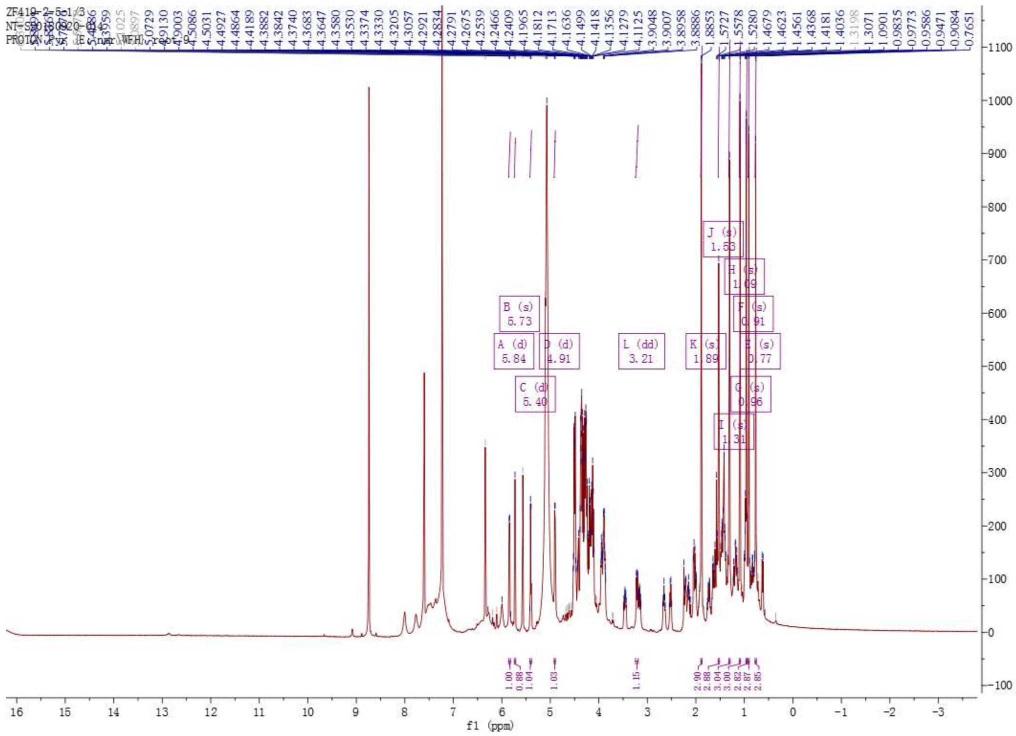

Figure S37. ^{1}H NMR spectrum of compound **6**

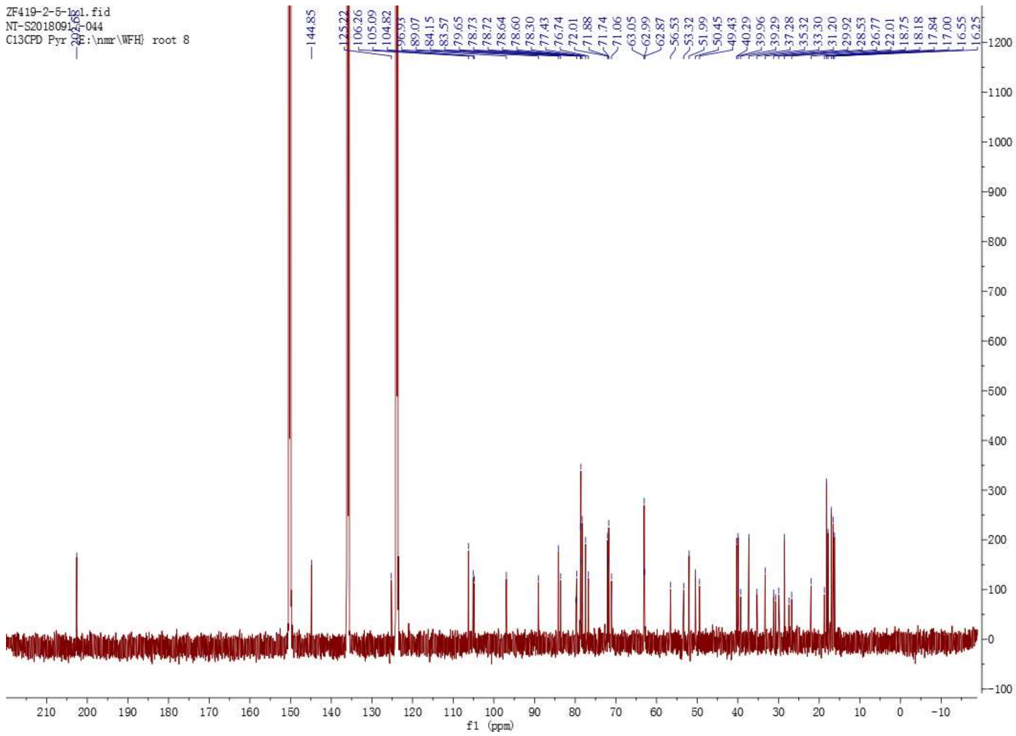

Figure S38. ^{13}C NMR spectrum of compound **6**

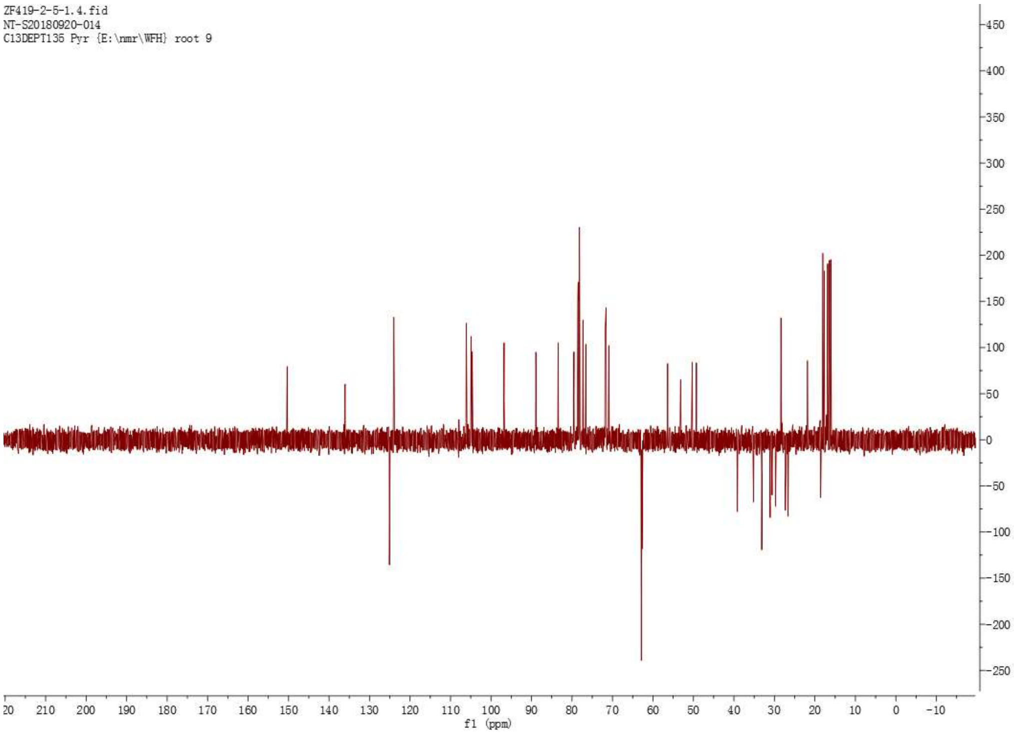

Figure S39. DEPT-135 spectrum of compound **6**

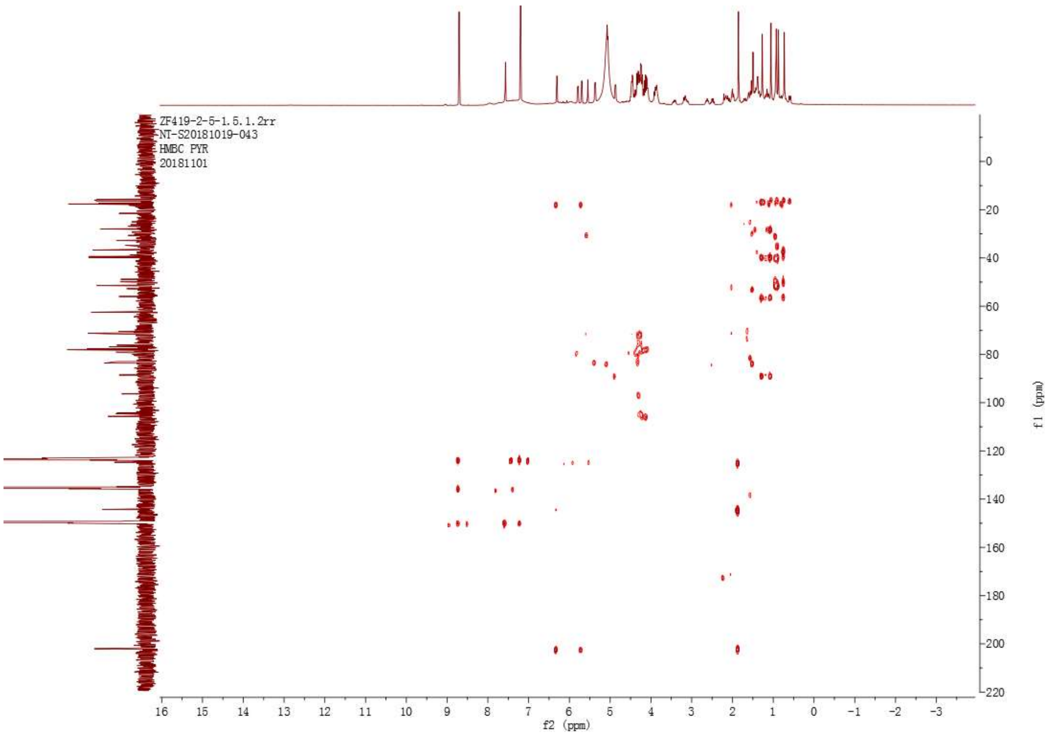

Figure S40. HMBC spectrum of compound **6**

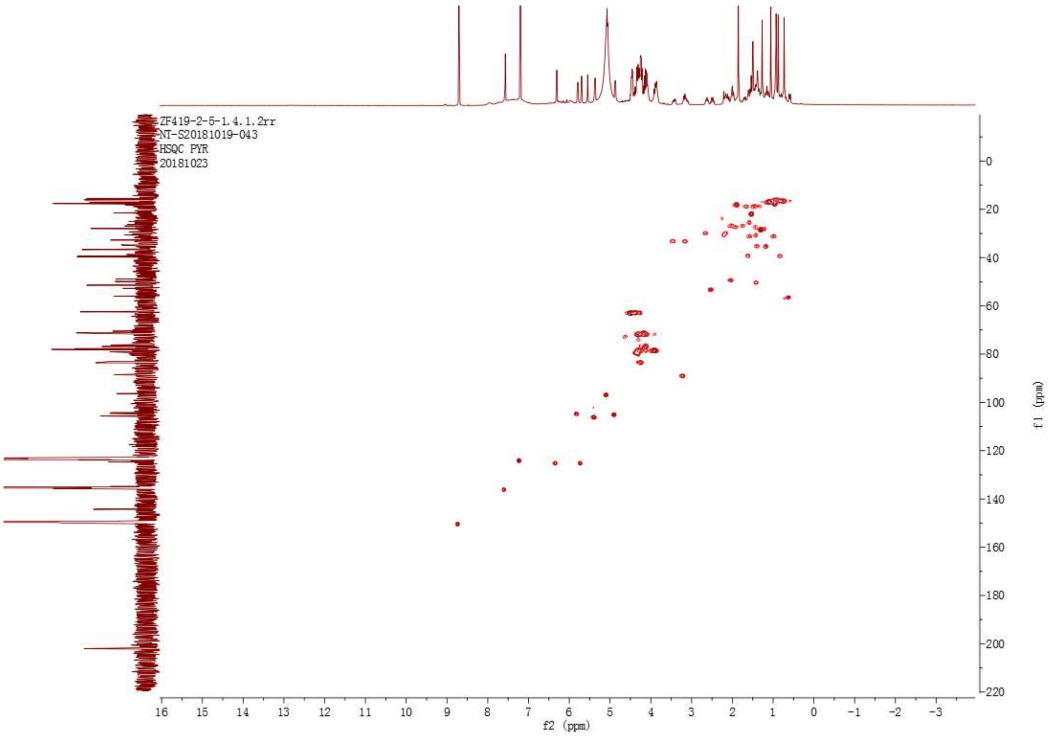

Figure S41. HSQC spectrum of compound **6**

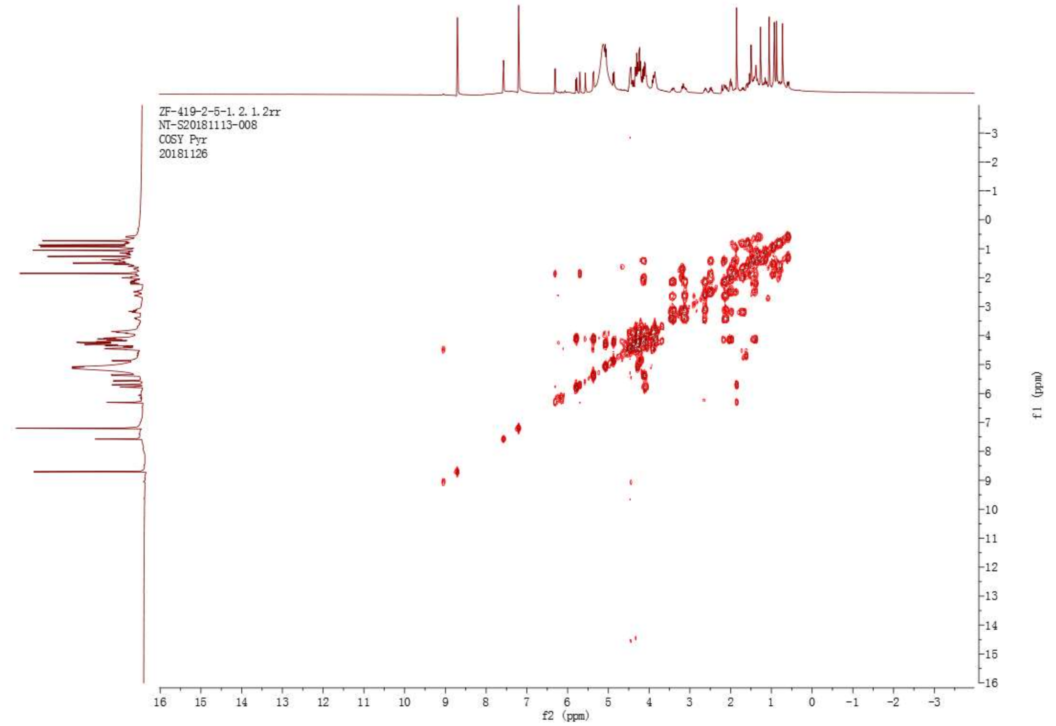

Figure S42. ^{1}H-^{1}H COSY spectrum of compound **6**

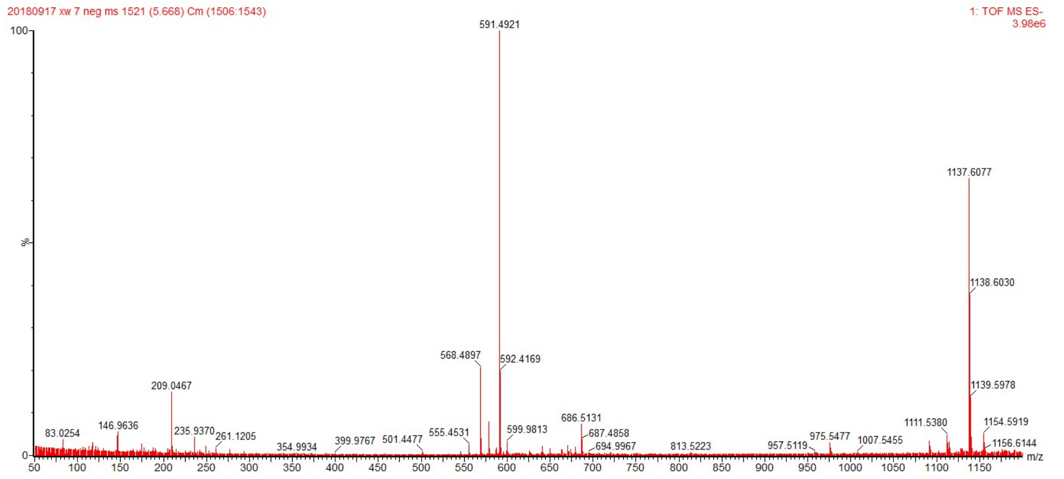

Figure S43. High-resolution electrospray ionization mass spectrum of compound **7**

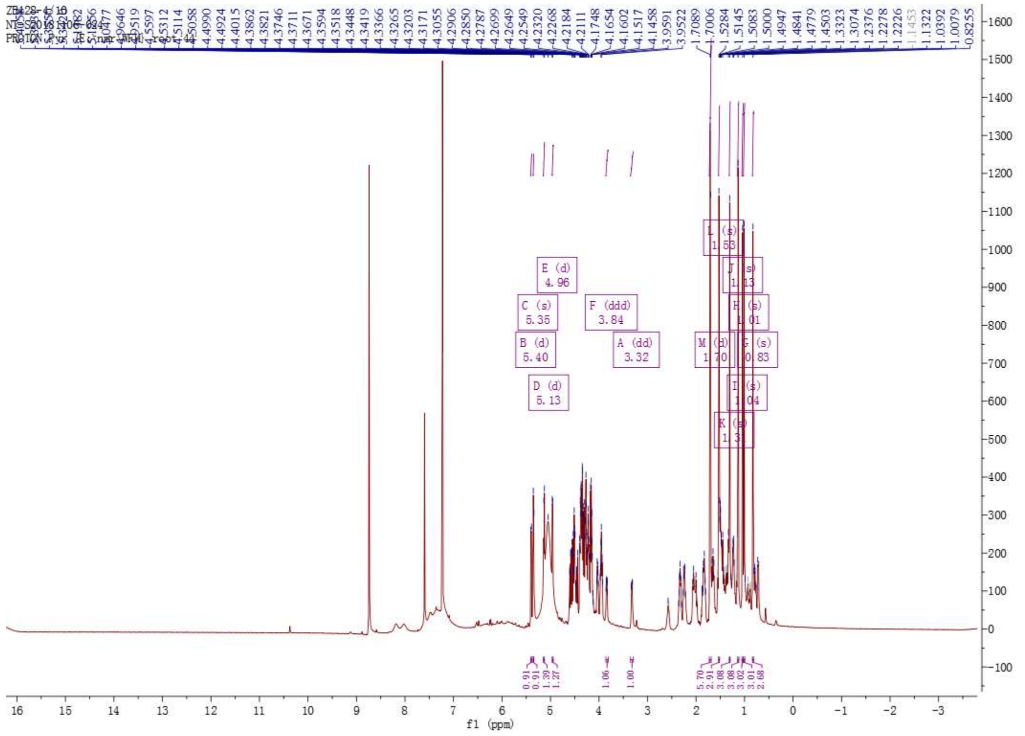

Figure S44. ^{1}H NMR spectrum of compound **7**

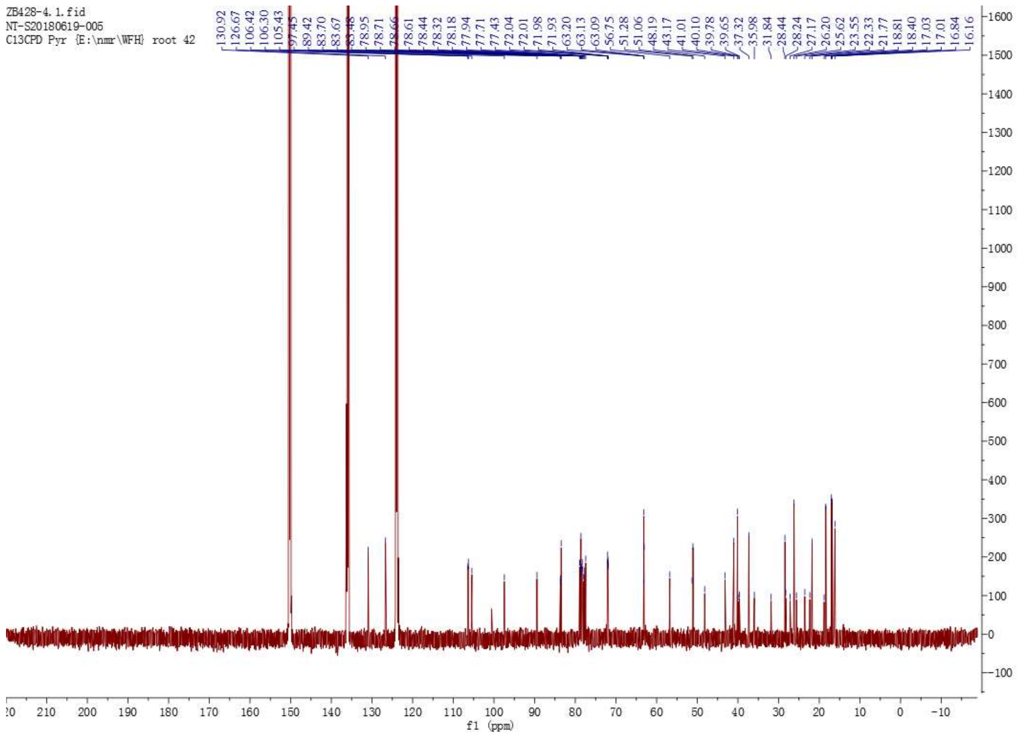

Figure S45. ^{13}C NMR spectrum of compound **7**

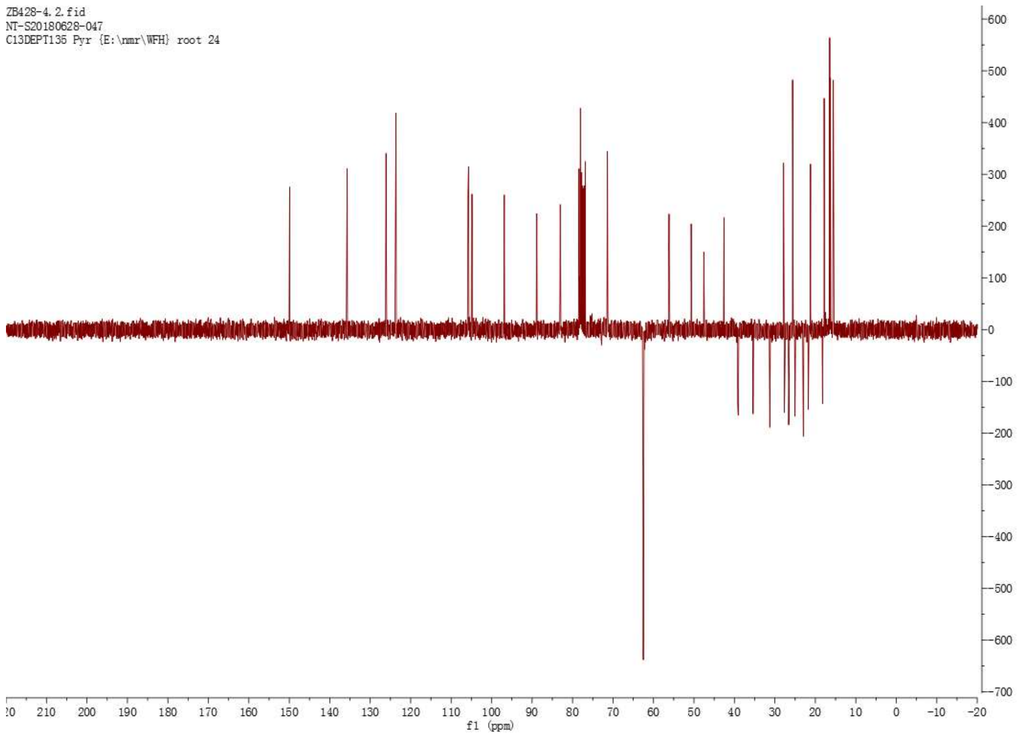

Figure S46. DEPT-135 spectrum of compound **7**

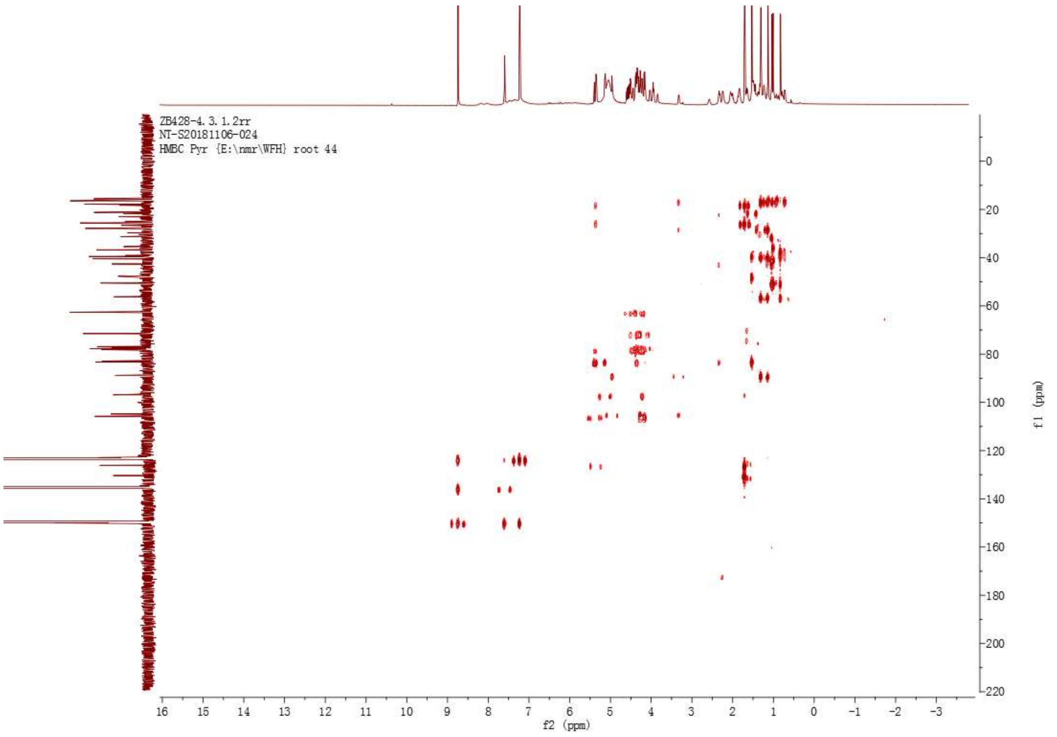

Figure S47. HMBC spectrum of compound **7**

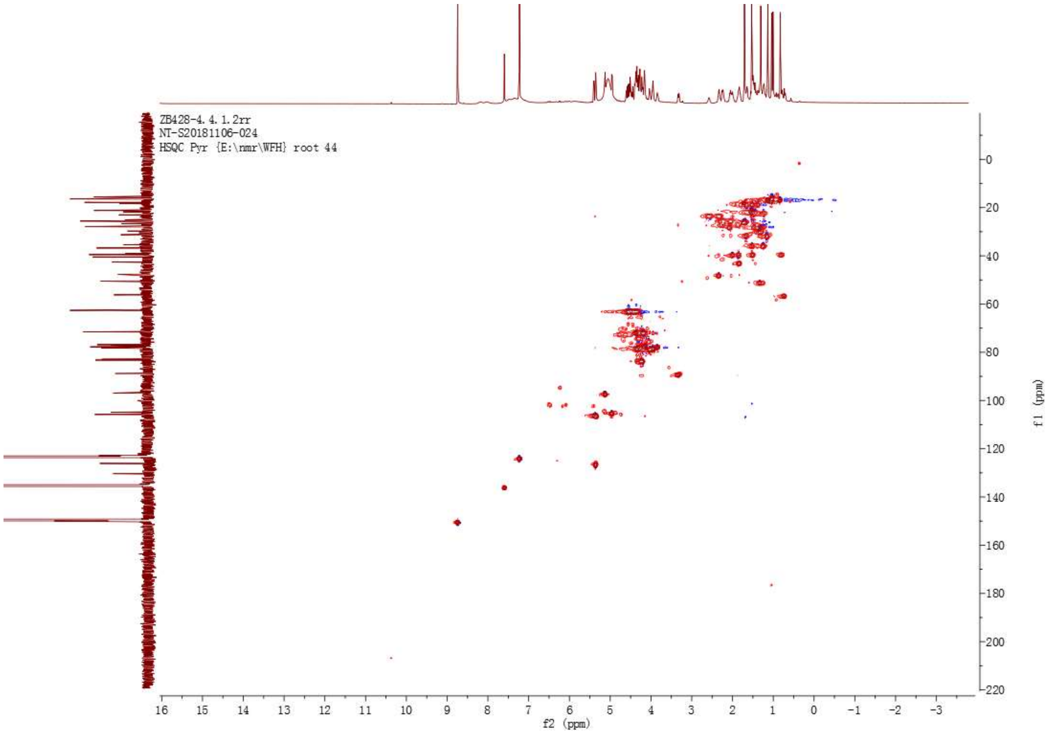

Figure S48. HSQC spectrum of compound **7**

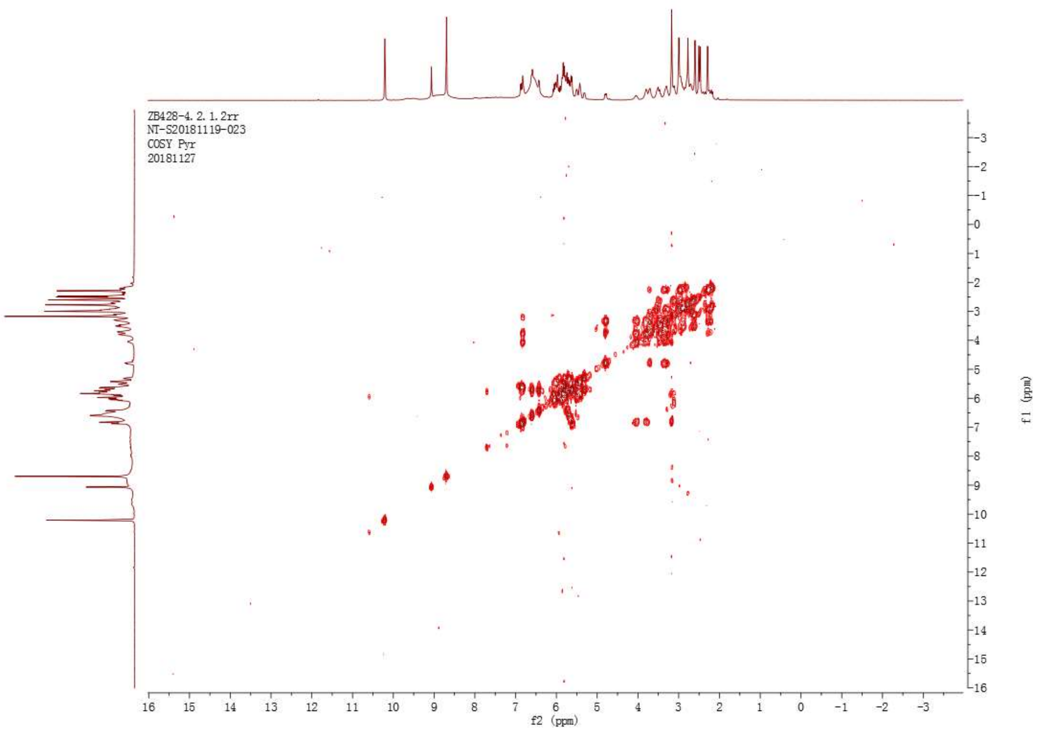

Figure S49. ^{1}H-^{1}H COSY spectrum of compound **7**

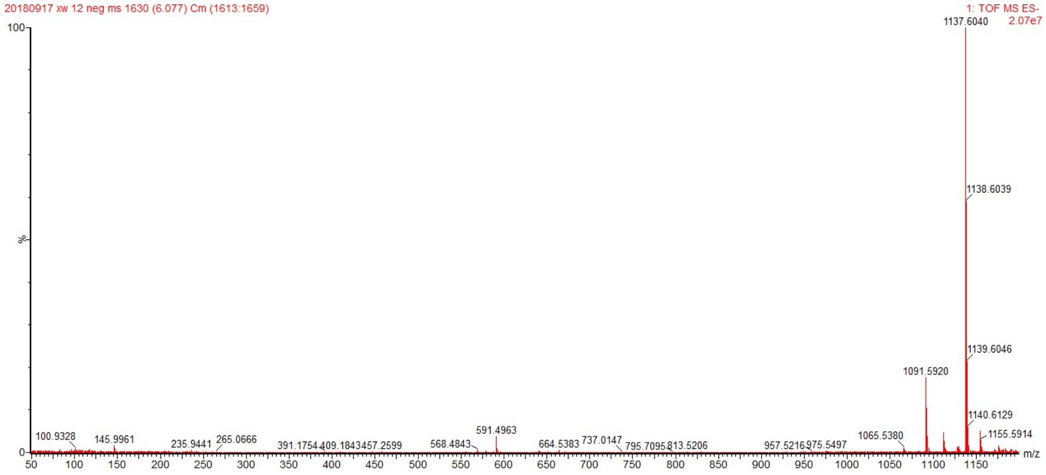

Figure S50. High-resolution electrospray ionization mass spectrum of compound **8**

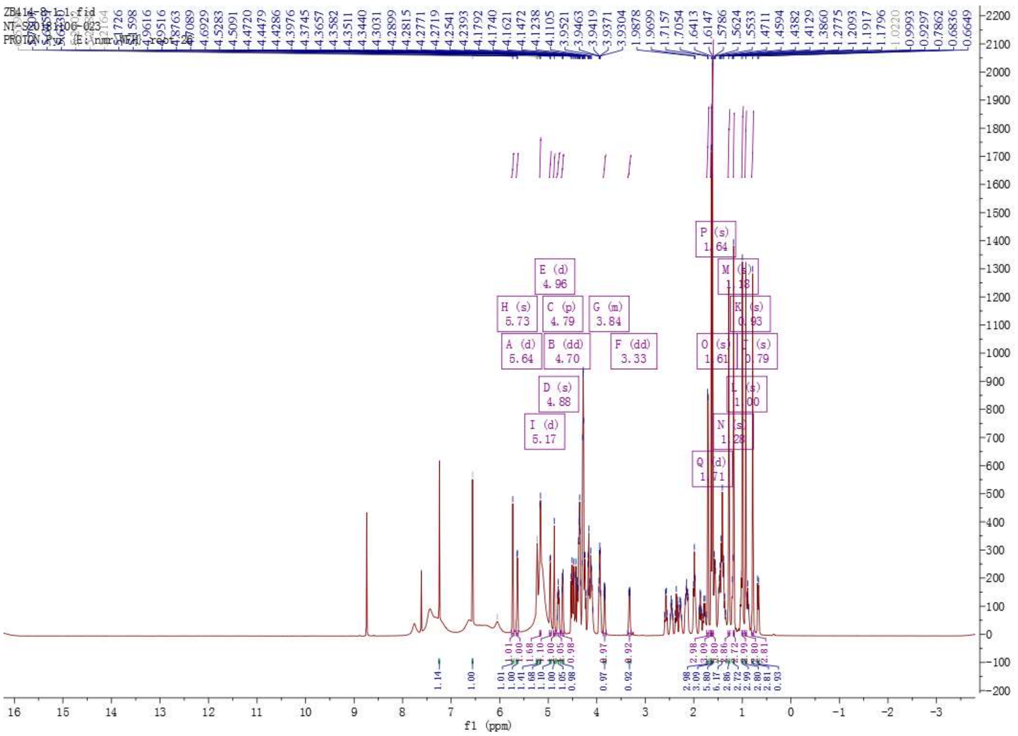

Figure S51. ^{1}H NMR spectrum of compound **8**

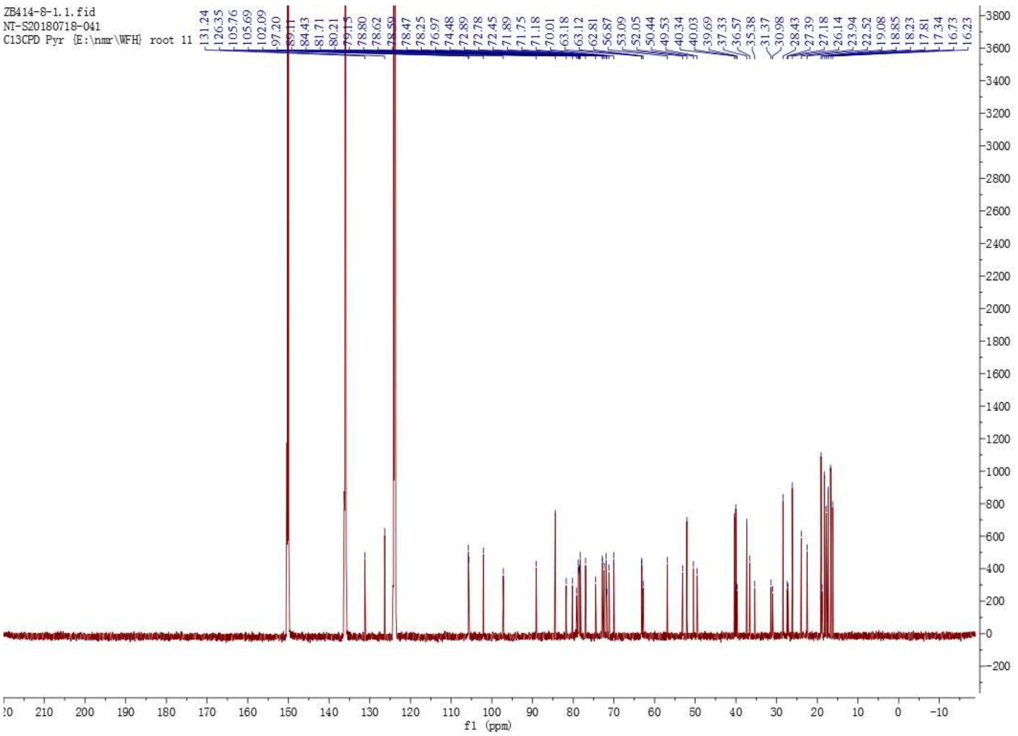

Figure S52. ^{13}C NMR spectrum of compound **8**

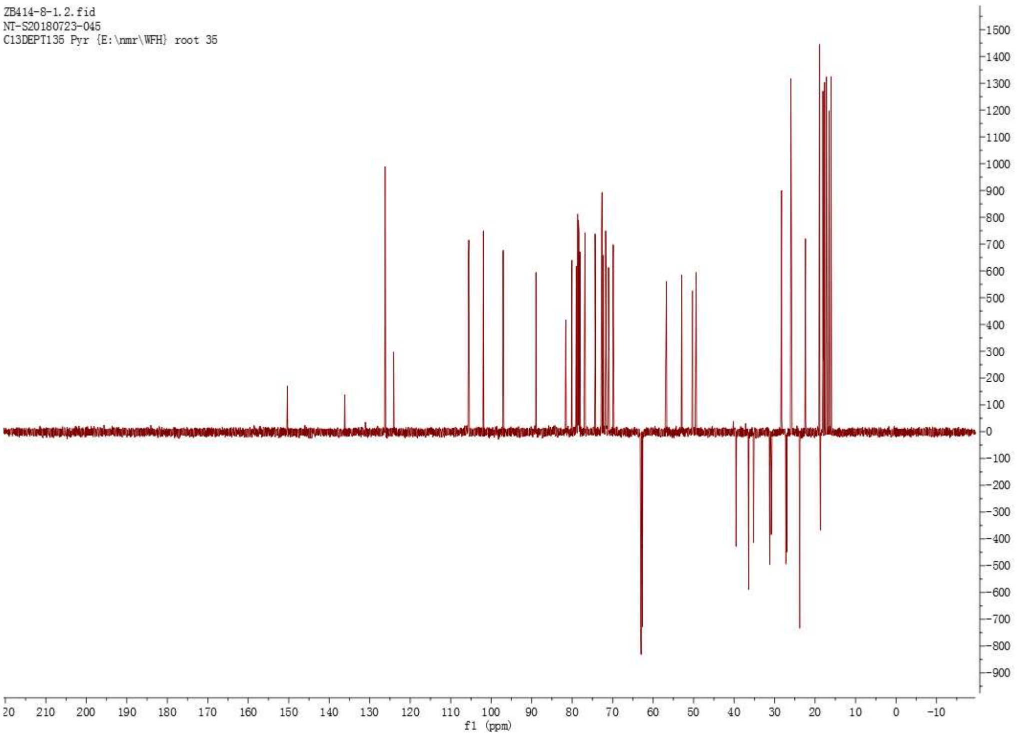

Figure S53. DEPT-135 spectrum of compound **8**

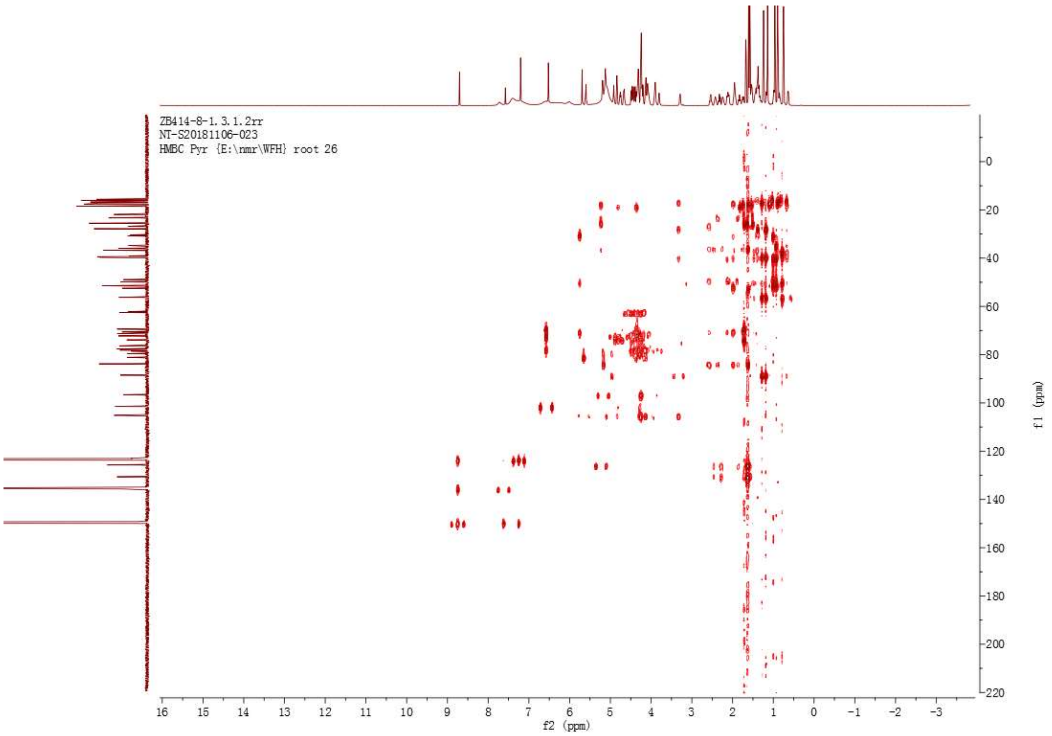

Figure S54. HMBC spectrum of compound **8**

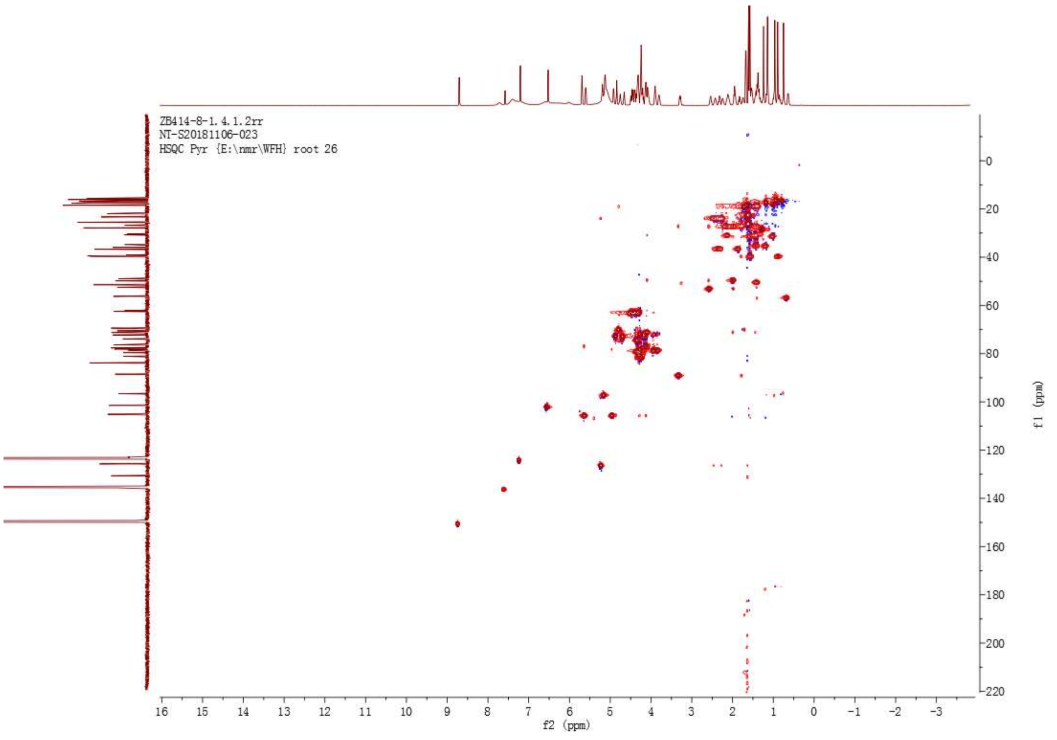

Figure S55. HSQC spectrum of compound **8**

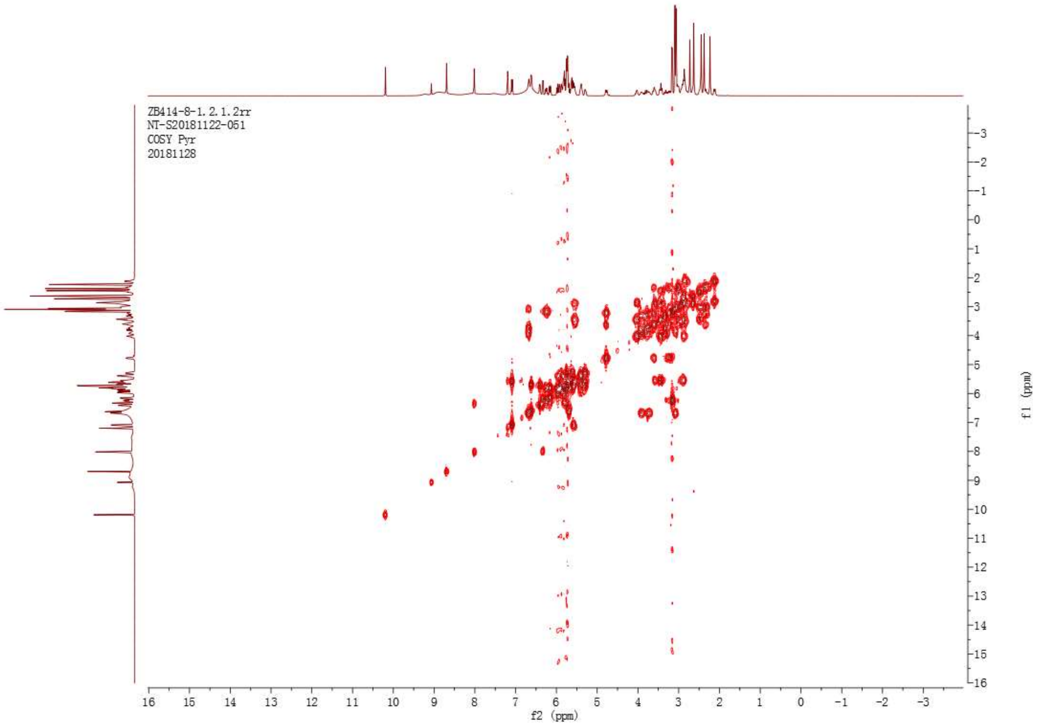

Figure S56. ^{1}H-^{1}H COSY spectrum of compound **8**

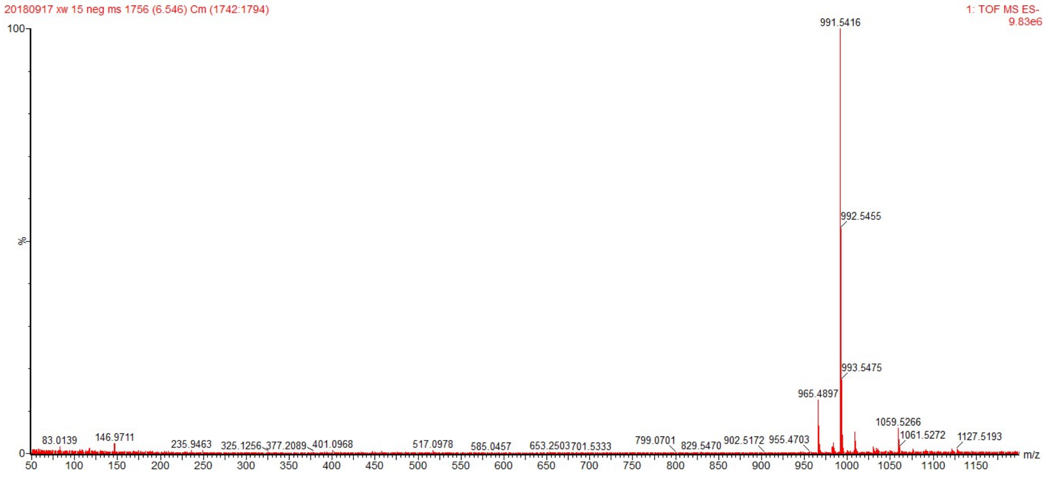

Figure S57. High-resolution electrospray ionization mass spectrum of compound **9**

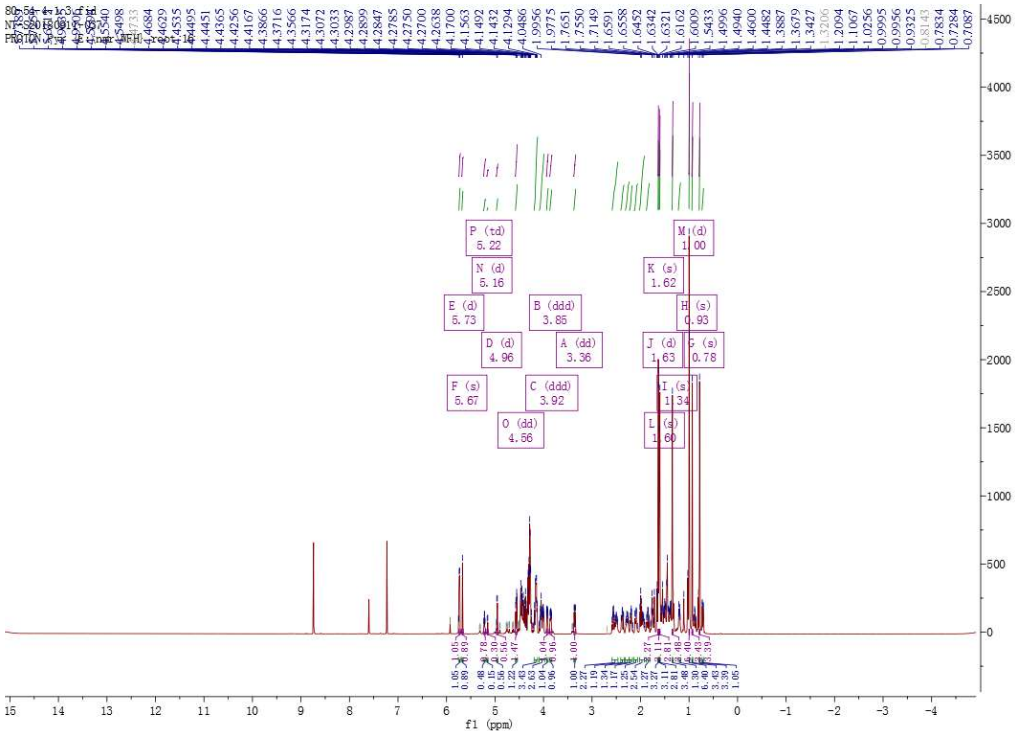

Figure S58. ^{1}H NMR spectrum of compound **9**

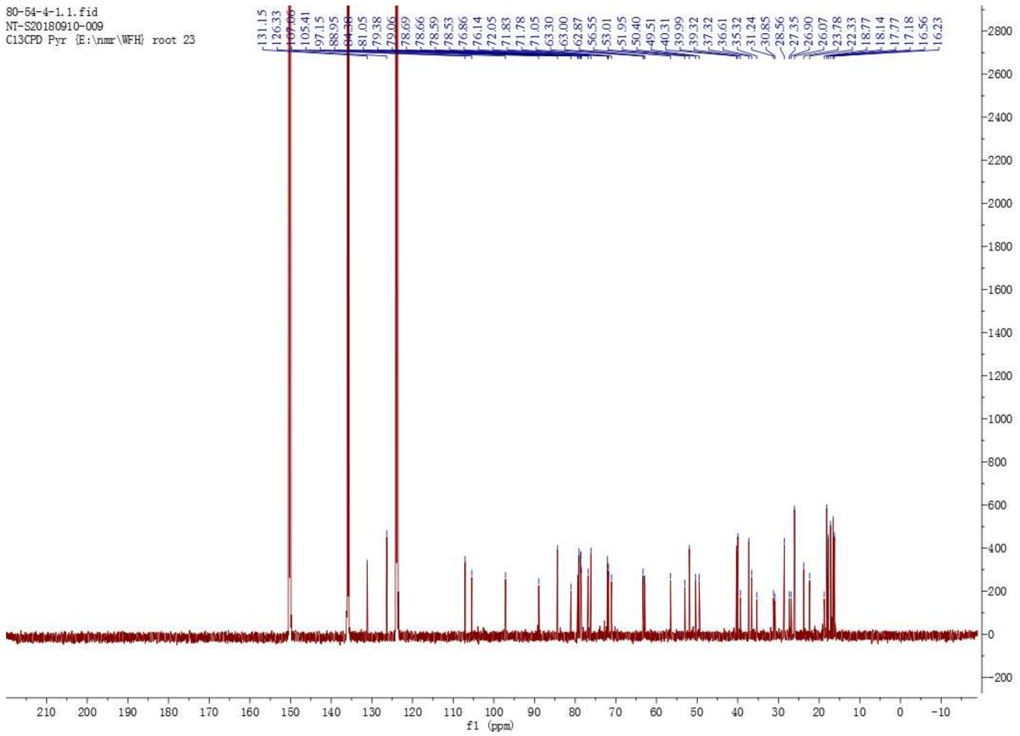

Figure S59. ^{13}C NMR spectrum of compound **9**

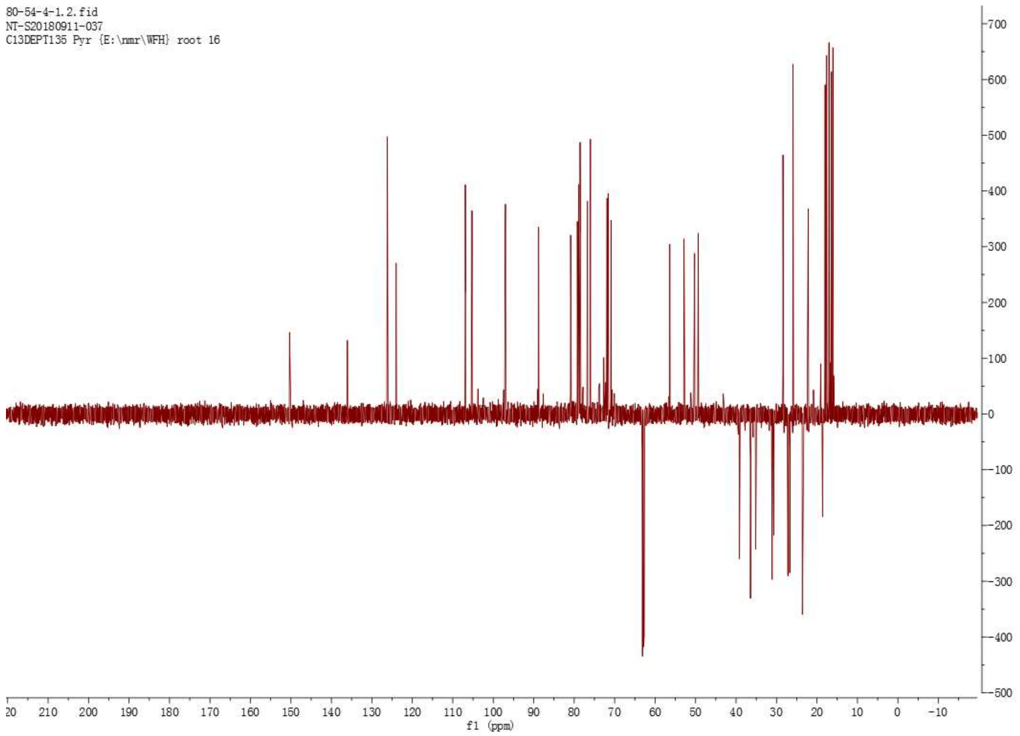

Figure S60. DEPT-135 spectrum of compound **9**

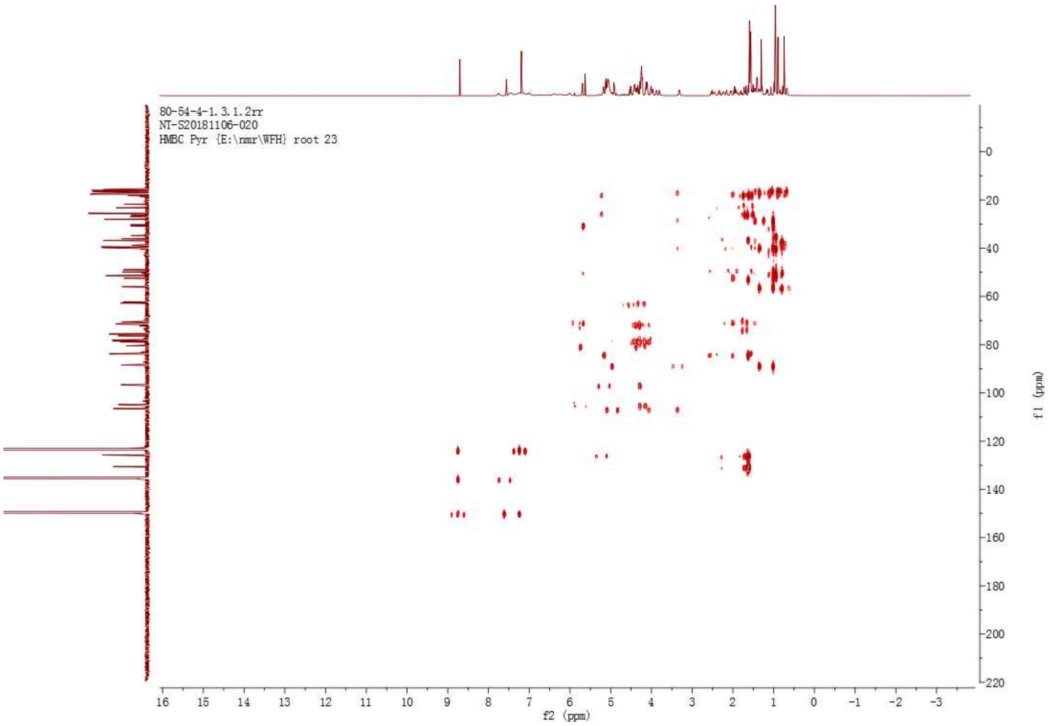

Figure S61. HMBC spectrum of compound **9**

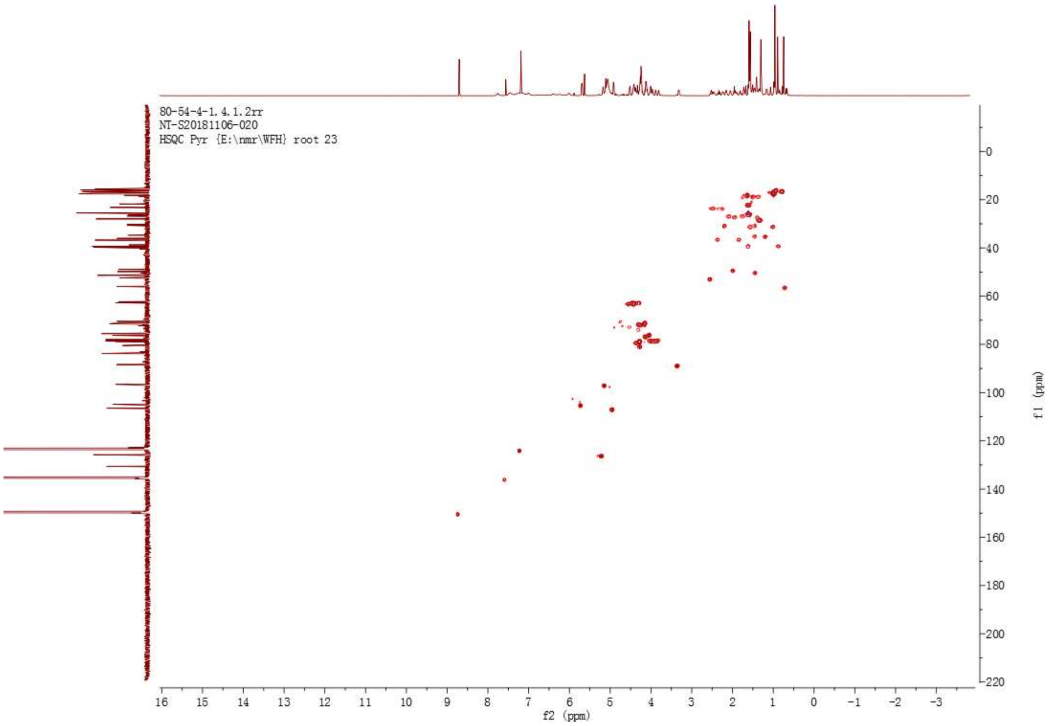

Figure S62. HSQC spectrum of compound **9**

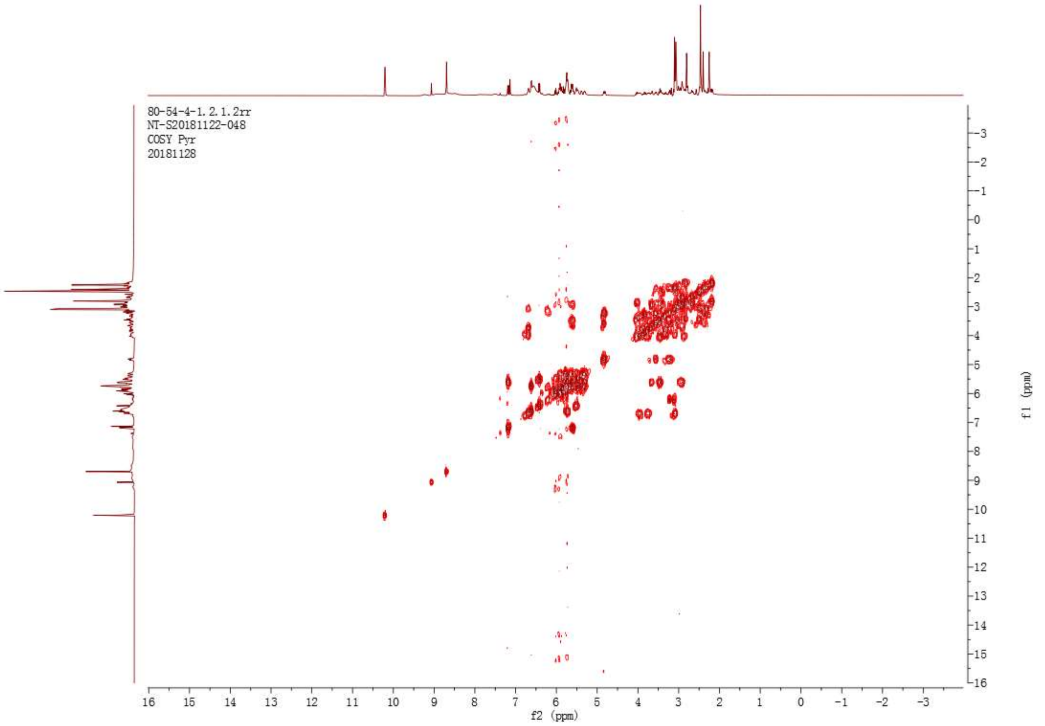

Figure S63. ^{1}H-^{1}H COSY spectrum of compound **9**

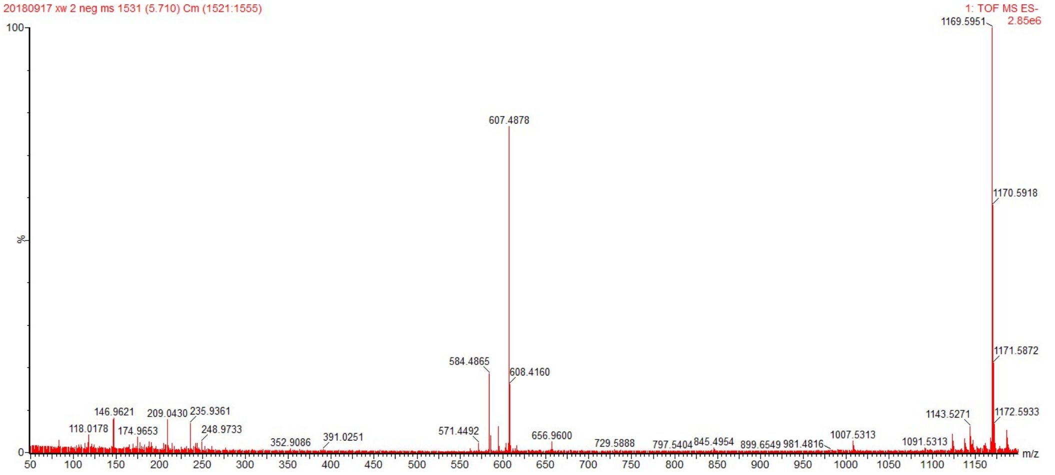

Figure S64. High-resolution electrospray ionization mass spectrum of compound **10**

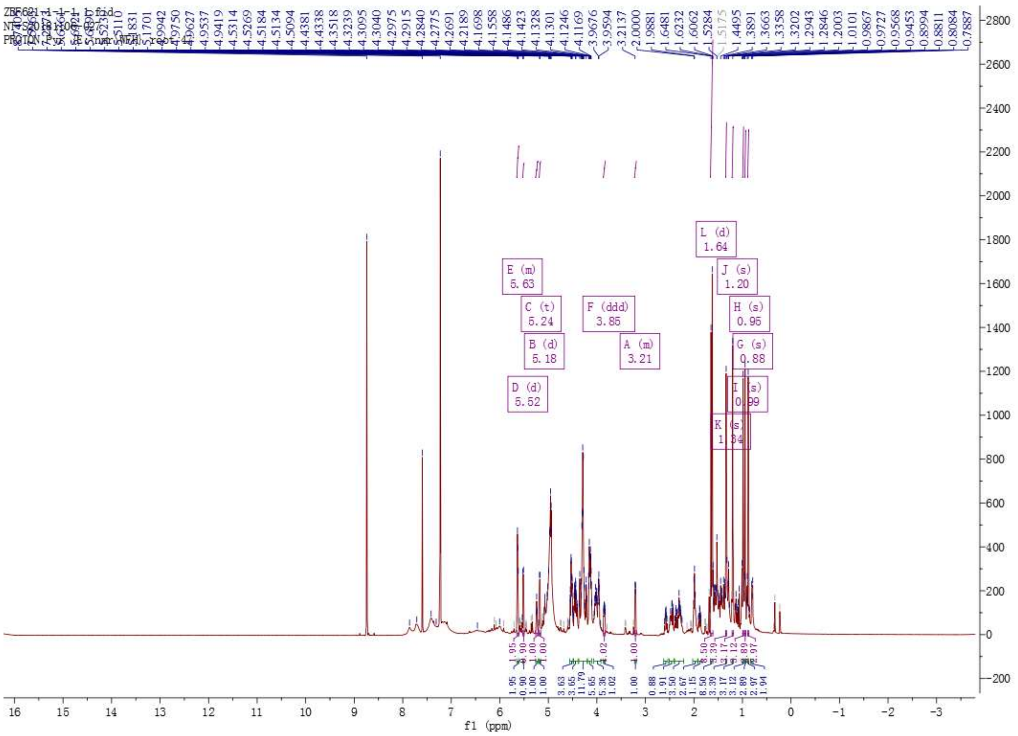

Figure S65. ^{1}H NMR spectrum of compound **10**

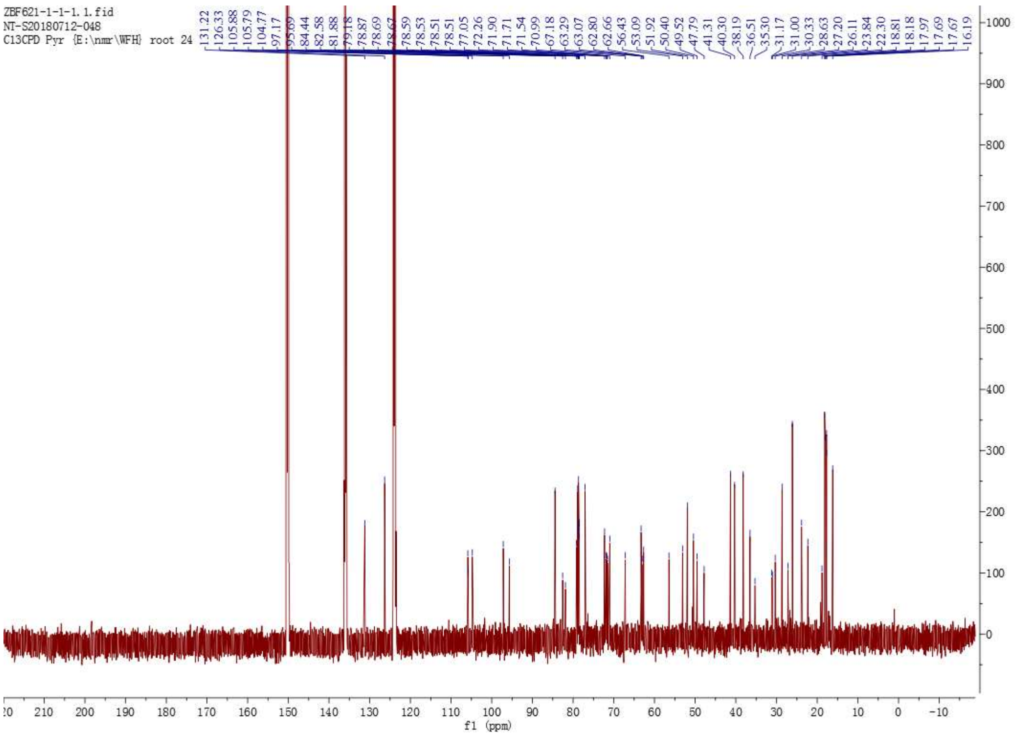

Figure S66. ^{13}C NMR spectrum of compound **10**

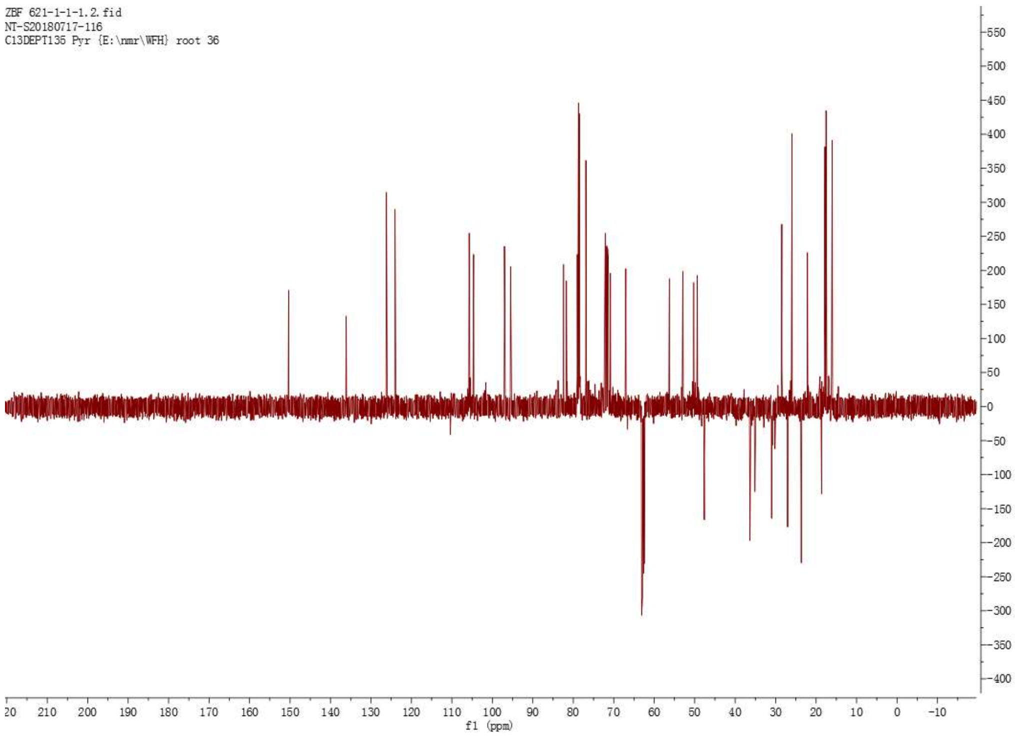

Figure S67. DEPT-135 spectrum of compound **10**

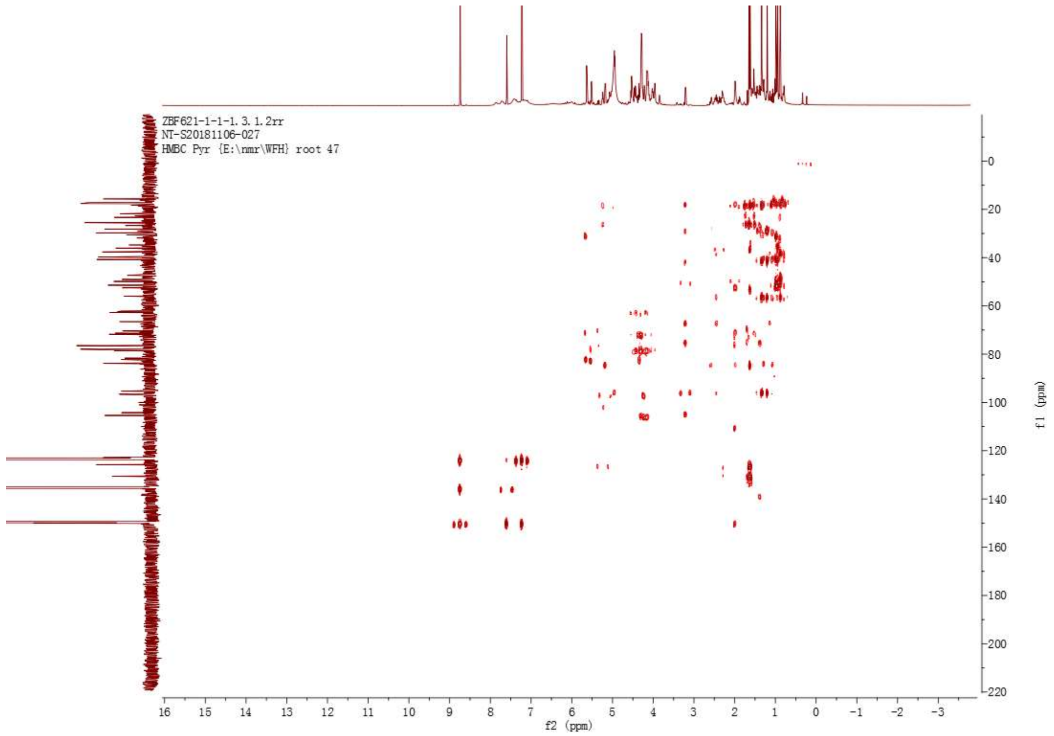

Figure S68. HMBC spectrum of compound **10**

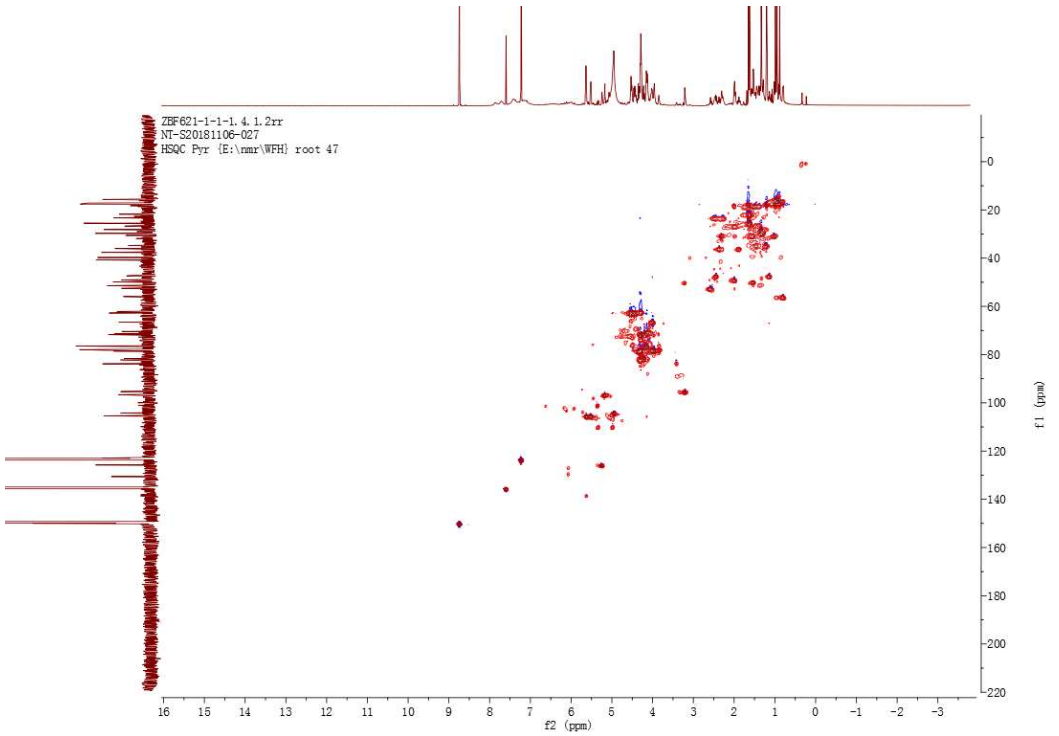

Figure S69. HSQC spectrum of compound **10**

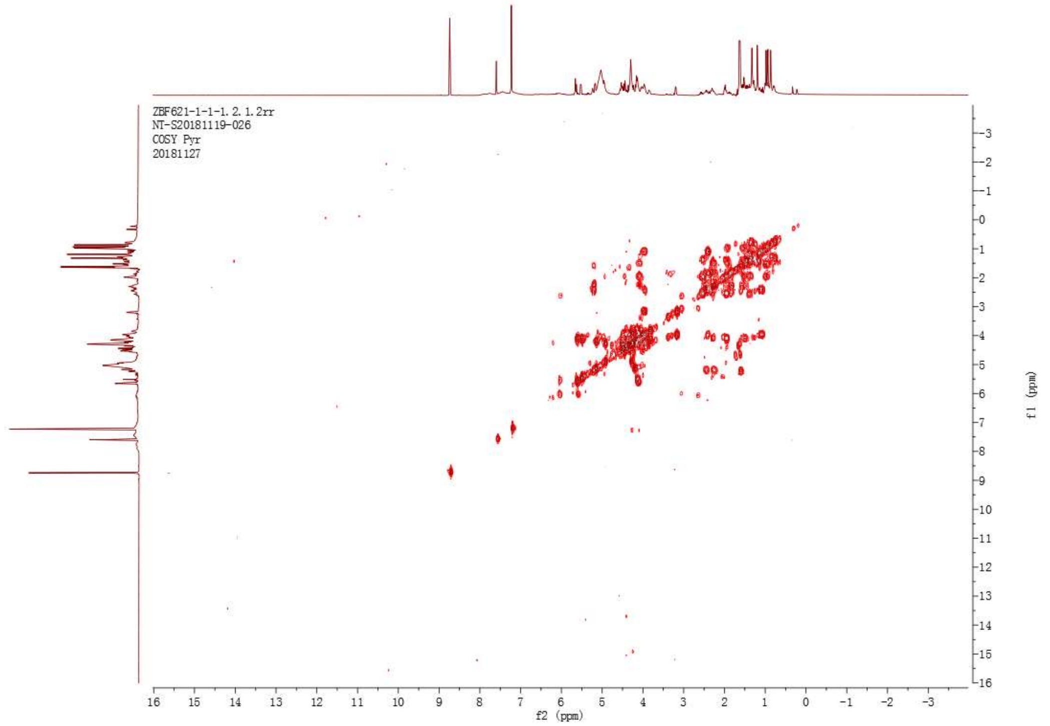

Figure S70. ^{1}H-^{1}H COSY spectrum of compound **10**

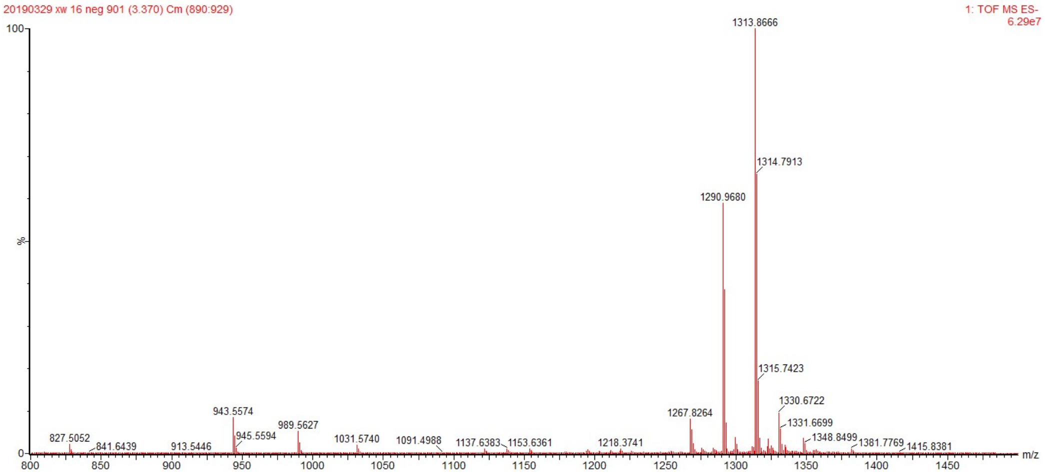

Figure S71. High-resolution electrospray ionization mass spectrum of compound **11**

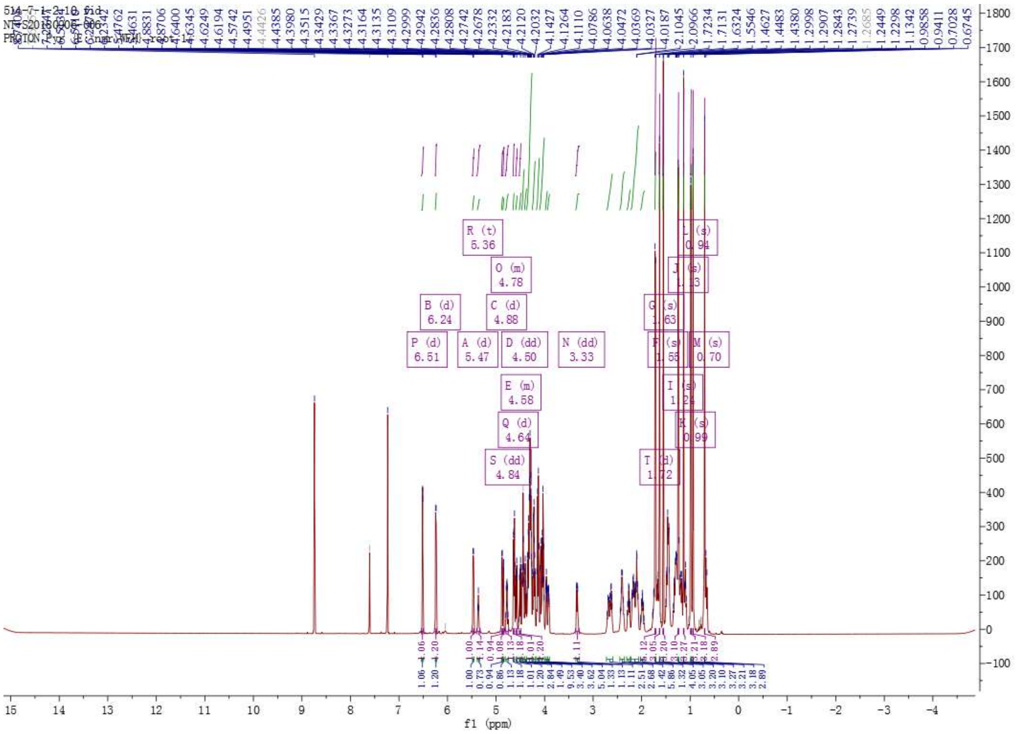

Figure S72. ^{1}H NMR spectrum of compound **11**

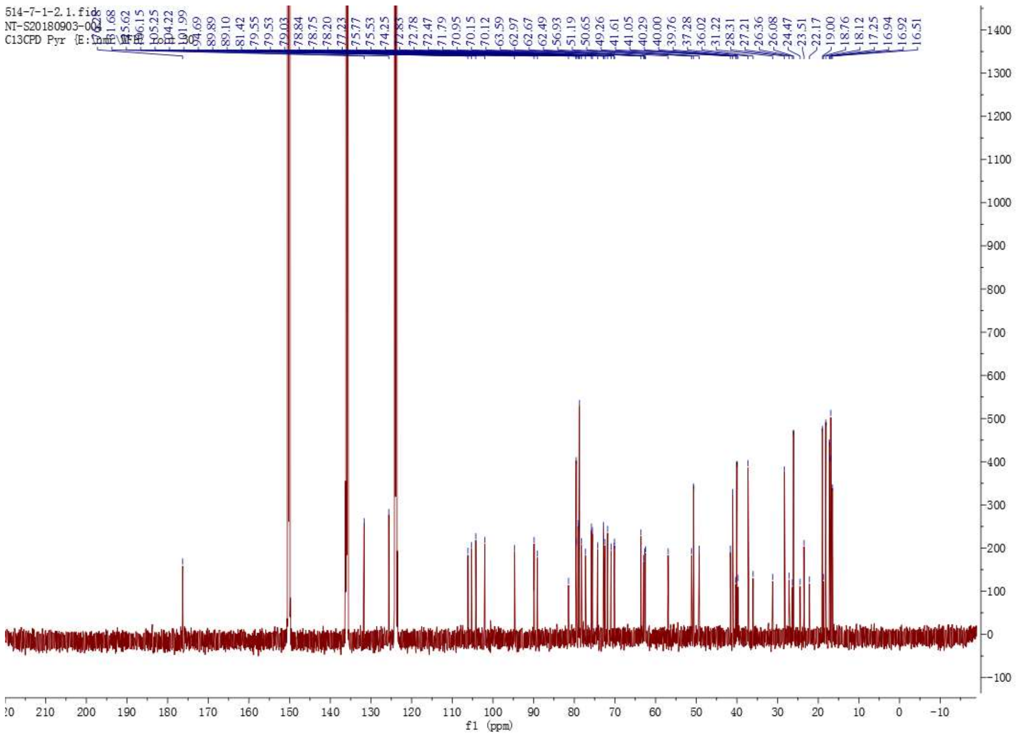

Figure S73. ^{13}C NMR spectrum of compound **11**

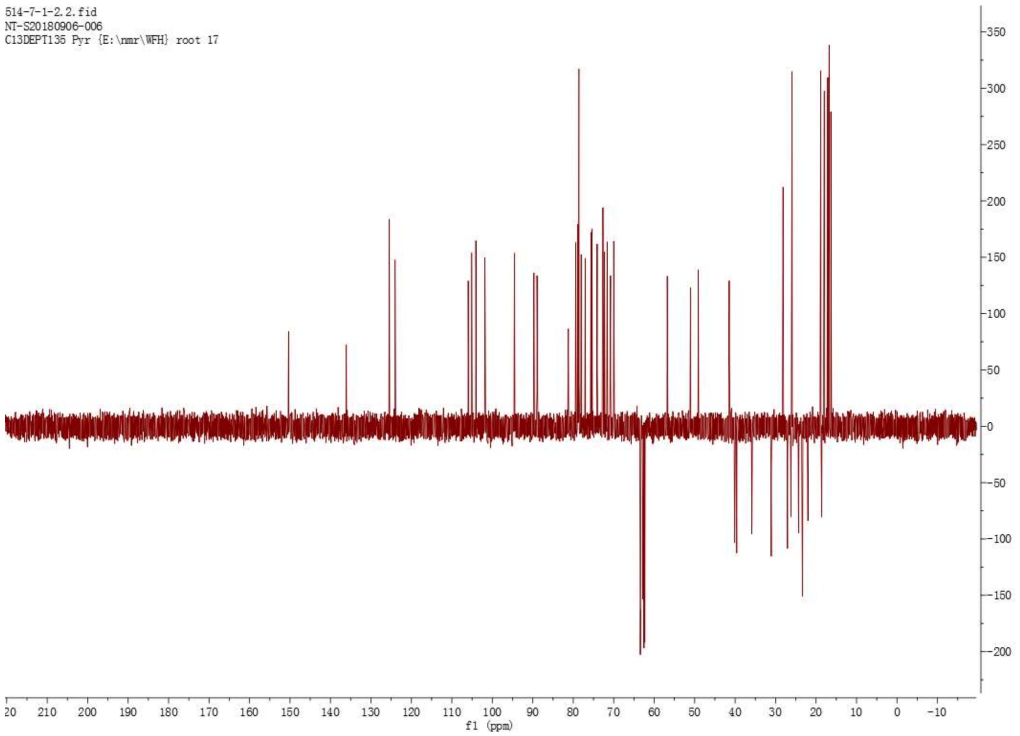

Figure S74. DEPT-135 spectrum of compound **11**
